# Stretching Peptides to Generate Small Molecule β-Strand Mimics

**DOI:** 10.1021/acscentsci.2c01462

**Published:** 2023-03-15

**Authors:** Zoë C. Adams, Anthony P. Silvestri, Sorina Chiorean, Dillon T. Flood, Brian P. Balo, Yifan Shi, Matthew Holcomb, Shawn I. Walsh, Colleen A. Maillie, Gregory K. Pierens, Stefano Forli, K. Johan Rosengren, Philip E. Dawson

**Affiliations:** † Department of Chemistry, The Scripps Research Institute, 10550 North Torrey Pines Road, La Jolla, California 92037, United States; ‡ Department of Integrated Structural and Computational Biology, The Scripps Research Institute, 10550 North Torrey Pines Road, La Jolla, California 92037, United States; § Centre for Advanced Imaging, University of Queensland, Brisbane, Queensland 4072, Australia; ∥ Institute for Molecular Bioscience and School of Biomedical Sciences, University of Queensland, Brisbane, Queensland 4072, Australia; ⊥ Unnatural Products, Inc., 2161 Delaware Ave, Suite A., Santa Cruz, California 95060, United States

## Abstract

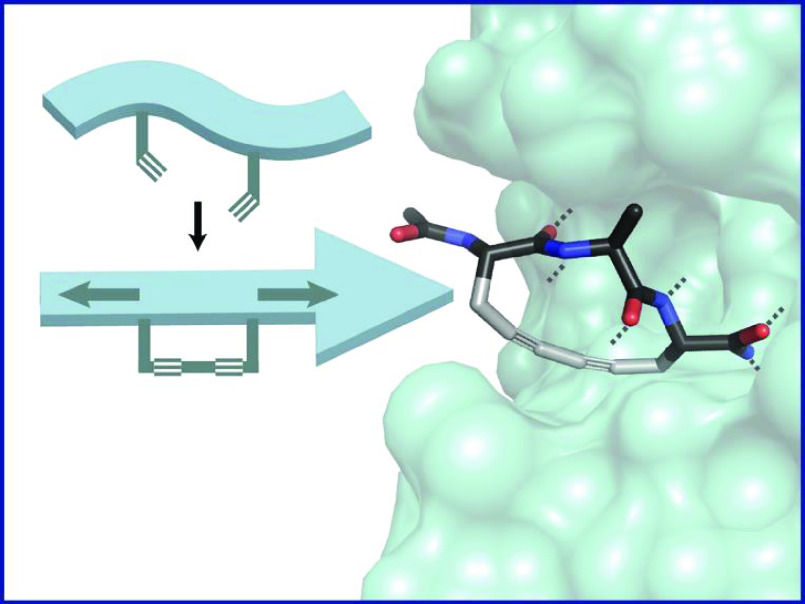

Advances in the modulation of protein–protein interactions (PPIs) enable both characterization of PPI networks that govern diseases and design of therapeutics and probes. The shallow protein surfaces that dominate PPIs are challenging to target using standard methods, and approaches for accessing extended backbone structures are limited. Here, we incorporate a rigid, linear, diyne brace between side chains at the *i* to *i+*2 positions to generate a family of low-molecular-weight, extended-backbone peptide macrocycles. NMR and density functional theory studies show that these stretched peptides adopt stable, rigid conformations in solution and can be tuned to explore extended peptide conformational space. The diyne brace is formed in excellent conversions (>95%) and amenable to high-throughput synthesis. The minimalist structure-inducing tripeptide core (<300 Da) is amenable to further synthetic elaboration. Diyne-braced inhibitors of bacterial type 1 signal peptidase demonstrate the utility of the technique.

## Abstract 2

Enforcing a β-strand mimicking structure in small peptides using an installed diyne rod has promise for developing antibiotics and other functional molecules.

## Introduction

Extended amino acid backbone conformations are an abundant structural motif responsible for mediating a myriad of protein–protein interactions (PPIs). Along with other secondary structures including turns and helices, extended regions present ordered backbone and side chain orientations that contribute to specific recognition of protein targets.^1−3^ Common modes of binding within protein–protein interaction domains include recognition of extended β-strand and type II polyproline (PPII) helical conformations.^4^ Modulation of these interactions requires mimicry of the specific and selective interactions between proteins, often originating from the PPII rich disordered regions present in over half of eukaryotic proteins.^5−7^ The development of designed peptides biased toward an extended β-strand or PPII helical conformation is relevant for solving diverse biological problems from addressing the “undruggable” population of the proteome^8−11^ to modeling or disrupting peptide aggregation.^12^ Chemical modifications of small to medium sized synthetic peptides, including the installation of known β-turn sequences, *N*-amination of the backbone, and macrocyclization via side chain-to-side chain or side chain-to-main chain linkages,^13−21^ have emerged as a powerful technique for accessing extended-backbone peptides. However, current synthetic strategies for mimicking extended structures are limited in their application since they typically require modifications that disrupt backbone hydrogen bonds defining the β-strand. Since we have previously shown that the Glaser coupling is compatible with peptides,^22,23^ we investigated the incorporation of *i*, *i+*2 diyne linkages into peptides to construct rigid, extended-backbone peptide macrocycles. In contrast to existing methods for entropically promoting desired backbone structures through stapling distant side chains together, our method of stretching through proximal side chains with a rigid low molecular weight brace enforces an extended structure by preventing intramolecular backbone hydrogen bonds.

We optimized on-resin Glaser coupling to synthesize a variety of macrocycles incorporated into several peptide scaffolds. Conformational ensembles of a series of diyne macrocycles were determined by NMR and density functional theory (DFT) studies. We found that all selected variations of the diyne-braced macrocycle resulted in diverse extended backbone conformations dictated by both ring size and stereochemistry. Our structural interrogation of this class of compounds provides insights into how these constraints could be used to mimic the backbone structures observed in peptide ligands, inhibitors, and natural products. To demonstrate the practical utility of this novel class of compounds, we chose to target type 1 signal peptidase (SPase), a validated antibiotic target that binds peptide substrates in an extended conformation.^24−26^

**Figure 1:**
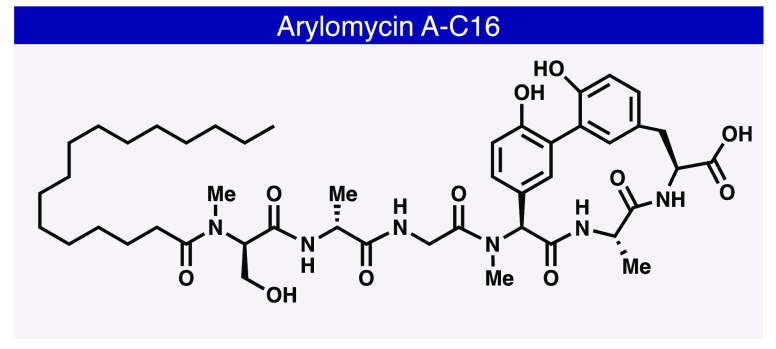
Structure of arylomycin A-C_16_ for reference.

We synthesized an array of *i, i+*2 diyne-braced peptides and incorporated modifications inspired by arylomycin (Figure 1), a natural product that targets SPase. Minimum inhibitory concentration (MIC) studies revealed these “alkynomycin” variants inhibit bacterial growth at low μM despite minimal design optimization. This result implies that the backbone conformation conferred by diyne-bracing alone was sufficient to emulate the activity of arylomycin and highlights the potential for further optimization of diyne macrocycles as antibiotics. Taken together, these studies support peptide stretching with diyne linkages as a valuable addition to the toolbox for peptide mimicry, with broad application to molecular targets that bind peptides with extended backbone structures.

## Results and Discussion

### Synthetic Optimization of Diyne Macrocycles

Although prior efforts have established the inter- and intramolecular use of the Glaser coupling in peptides,^22,23,27−30^ we were interested in developing a robust method to access *i, i+*2 peptide macrocycles quickly and efficiently (Table 1A). We previously reported on- and off-resin Glaser coupling for bioconjugation and stabilization of α-helix secondary structures.^22,23^ Přibylka et al. reported a strategy for stretching peptides via in solution formation of a diyne rod installed through perturbative peptoid linkages to the backbone.^29^ Since *i, i+*2 macrocycles have only 13–17 atoms, we anticipated a high level of strain and distorted bond angles, and thus predicted synthetic challenges in forming the diyne bond. However, this anticipated strain is also a key design feature to limit the degrees of freedom in the peptide backbone. To facilitate the synthesis of diyne peptide analogs, we optimized Glaser coupling conditions for peptides on polystyrene beads.

**Table 1:**
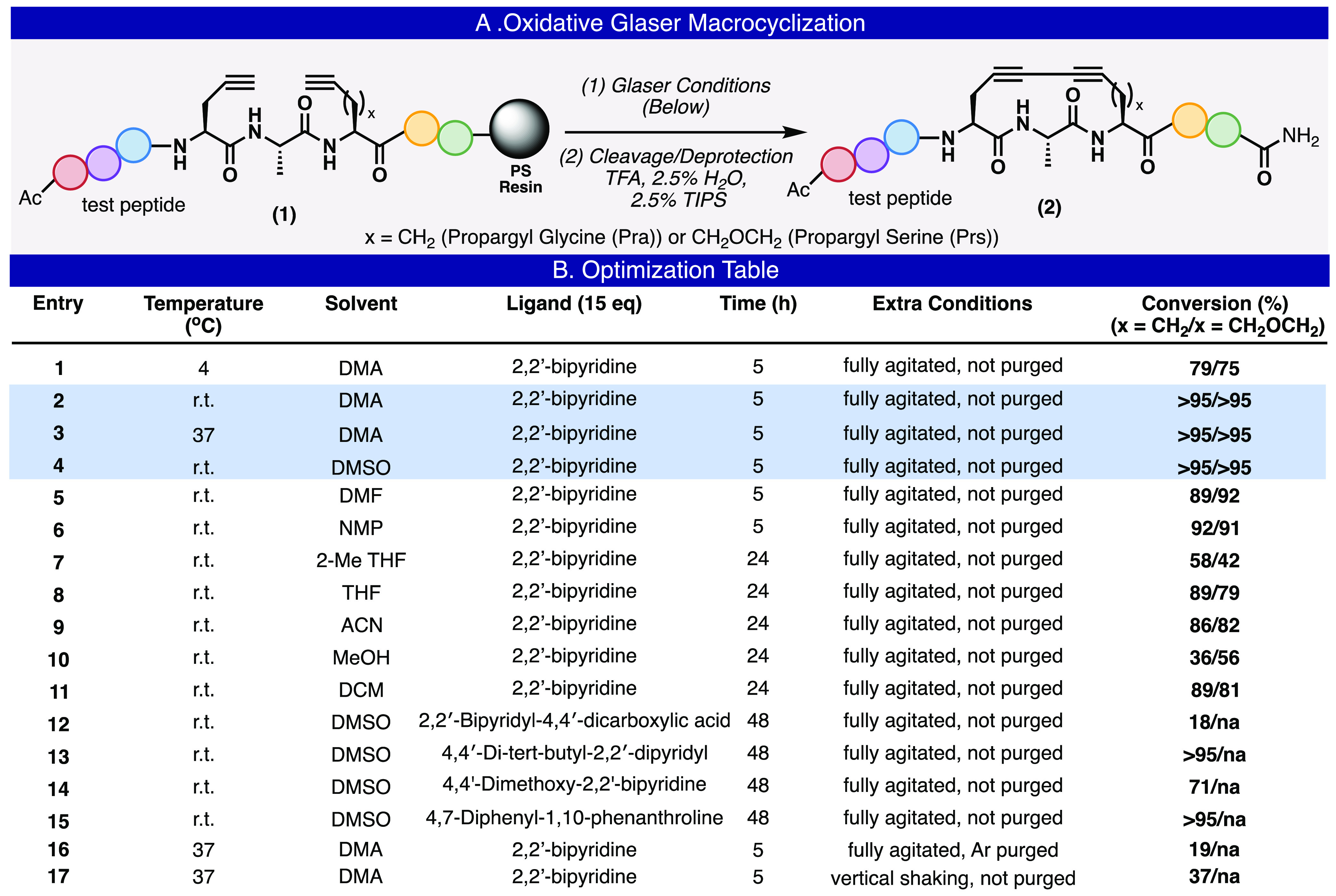
Optimization of Glaser Coupling Conditions^a^

a All conditions include 10 equiv CuCl, 15 equiv specified ligand, and 20 equiv DIPEA. Selected optimal conditions highlighted.

Screening bipyridine ligands with an excess of CuCl and *N,N*-diisopropylethylamine in DMF yielded several candidates that efficiently promoted Cu-mediated diyne macrocycle formation on polystyrene resin (Table 1B, entries 4, 12–15). The most economical ligand, 2,2′-bipyridine, produced a cyclized product with >95% conversion. Using this ligand, we screened the Glaser coupling with a series of solvents on two compounds containing propargylglycine (Pra) and propargylserine (Prs) residues, Pra-Ala-Pra and Pra-Ala-Prs (Table 1B, entries 2, 4–11). Although multiple solvents were acceptable, DMA was selected as an economical and readily available solvent. Varying the reaction temperature between 4 and 37 °C revealed that low temperature slows the reaction (Table 1B, entries 1–3). Critically, saturation of the reaction mixture in air was shown to be key for the reaction, likely to facilitate reoxidization of the Cu catalyst by O_2_. The small surface area provided by a vertical shaking orientation limited conversion to the macrocyclic product compared to the large surface area provided by a horizontal orientation (Table 1B, entry 17). To test this theory, we flushed the reaction vessel with argon to displace the atmosphere in the headspace and found that, indeed, conversion to the macrocyclic product was significantly reduced (Table 1B, entry 16). Optimized conditions yielded >95% conversion to the macrocyclic product in 5 h in DMA with 2,2′-bipyridine ligand at 37 °C with full agitation of the solution on both test peptide constructs (Table 1B, entry 3). Notably, despite the anticipated strain induced by the *i, i+*2 diyne, we were able to achieve similar conversions to those previously reported for larger macrocycles (>95% conversion, 3 days)^23^ in just five hours.

### Structural Characterization of Diyne Macrocyles

**Figure 2:**
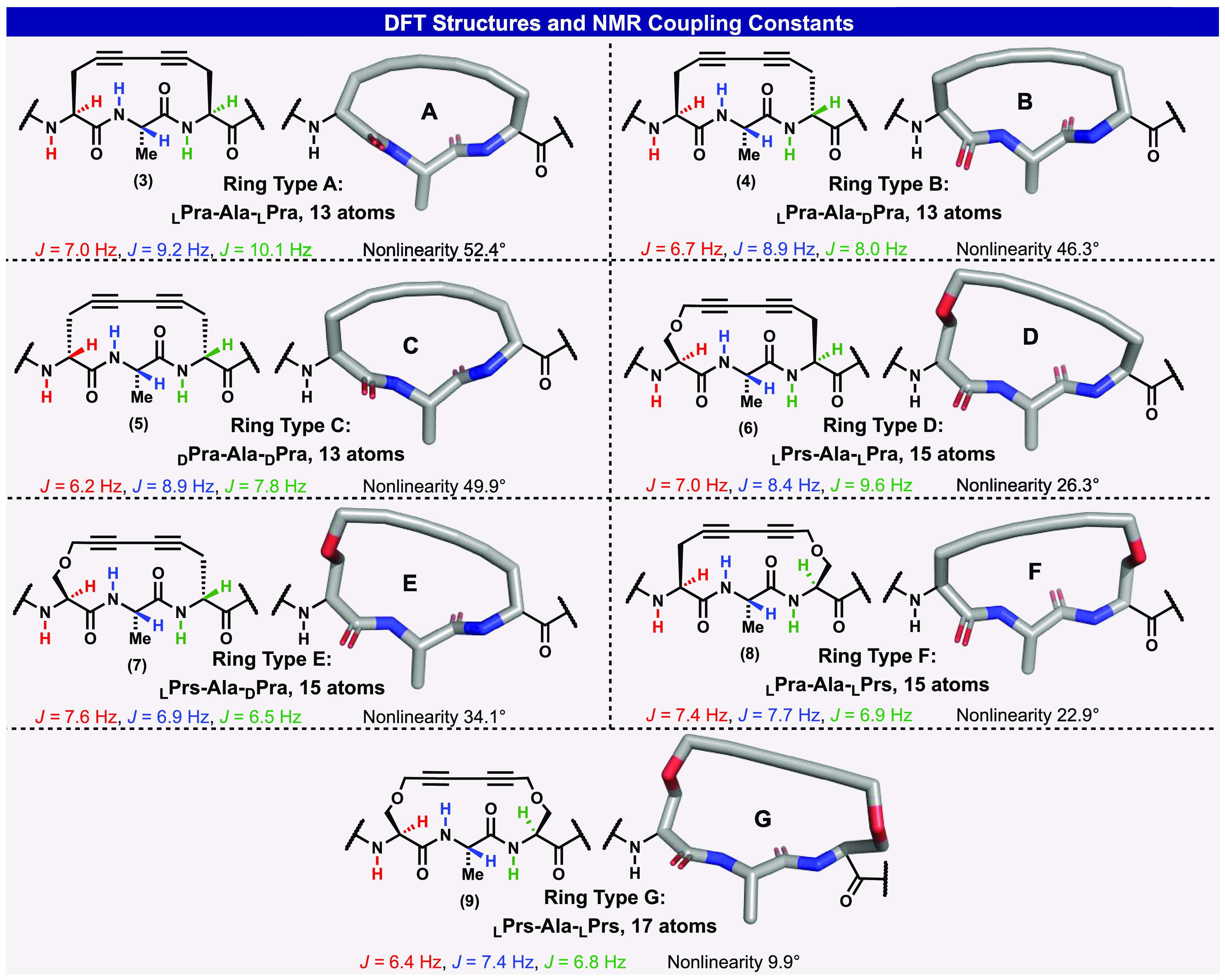
Density functional theory (DFT) calculations resulted in minimized energy structures for compounds containing 13-, 15-, or 17-membered rings. Notably, the backbone of these rigid macrocycles is preorganized into an extended β-strand conformation. NMR ^3^*J*_NHCHα_ coupling constant values support these DFT structures.

To study the impact of the diyne macrocycle on the structural characteristics of the peptide, a series of compounds were synthesized with varied macrocycle size and stereochemistry (Figure 2). The seven analogues contain pairs of l- and d-propargylglycine and l-propargylserine to form rings of sizes 13 atoms, 15 atoms, and 17 atoms. These macrocycles were selected to obtain a variety of extended backbone conformations that could be used to mimic natural peptide ligands.

Structural optimization of the library of macrocycles via DFT resulted in predictions of the lowest energy conformer for each ring type, shown in Figure 2. Notably, the diyne bond is perturbed from linearity, particularly in the smallest ring library members. The degree of nonlinearity was measured by the sum of the bond angle perturbation from the standard 180 deg for each of the four carbons spanning the diyne. This effect is well correlated to ring size; macrocycles with a ring size of 13 members were bent 40–50 deg out of linearity, while 15- and 17-membered macrocycles were bent by 20–30 and 10 deg, respectively (Table S1). Notably, the creation of a macrocycle with this level of bond distortion would likely be impossible without the binuclear copper transition state that organizes the alkynes and facilitates the formation of the diyne bond.

The DFT structures analyzed correlate well with experimental NMR data, supporting that these calculations are a good representation of the physical molecules. Coupling constants were calculated for the amide and alpha protons (^3^*J*_NHCHα_) of amino acid residues within the macrocycle (propargyl-containing residues 2 and 4, and Ala3) for compounds containing each of the macrocycles in the library. According to the Karplus relation, coupling constant values typical of β-strands (^3^*J*_NHCHα_ 8–10 Hz) are distinct from those of α-helices (^3^*J*_NHCHα_ < 6 Hz) and other protein secondary structures.^14,31^ The coupling constants measured for all library members are consistent with various extended conformations (^3^*J*_NHCHα_ > 6 Hz), with coupling constants for 13-membered rings generally higher than 15-membered rings. Compound **A**, the tightest macrocycle with natural stereochemistry, exhibited the highest coupling constants. Interestingly, ring size is demonstrated to be the best predictor of extended character, regardless of the stereochemistry of the ring-forming amino acids.

The structural characteristics of the diyne motif complement structural requirements of extended peptide structures. The distance between residues *i, i+*2 in a β-strand is 7.0 Å, while the length of hexa-2,4-diyne, analogous to the rod installed in these peptide-based molecules, is 6.7 Å. From the DFT calculations of our library of molecules, the distance between the α-carbons of the macrocycle residues is between 6.6 and 7.0 Å (Table S2). Interestingly, the distance between the *i, i+*2 residue α-carbons for 15- and 17-membered macrocycles is consistently 7.0 Å, perfectly in tune with the expected distance for β-strand residues. Meanwhile, in 13-membered rings, this distance is less than 7.0 Å due to the pronounced pucker of the alanine residue.

**Figure 3:**
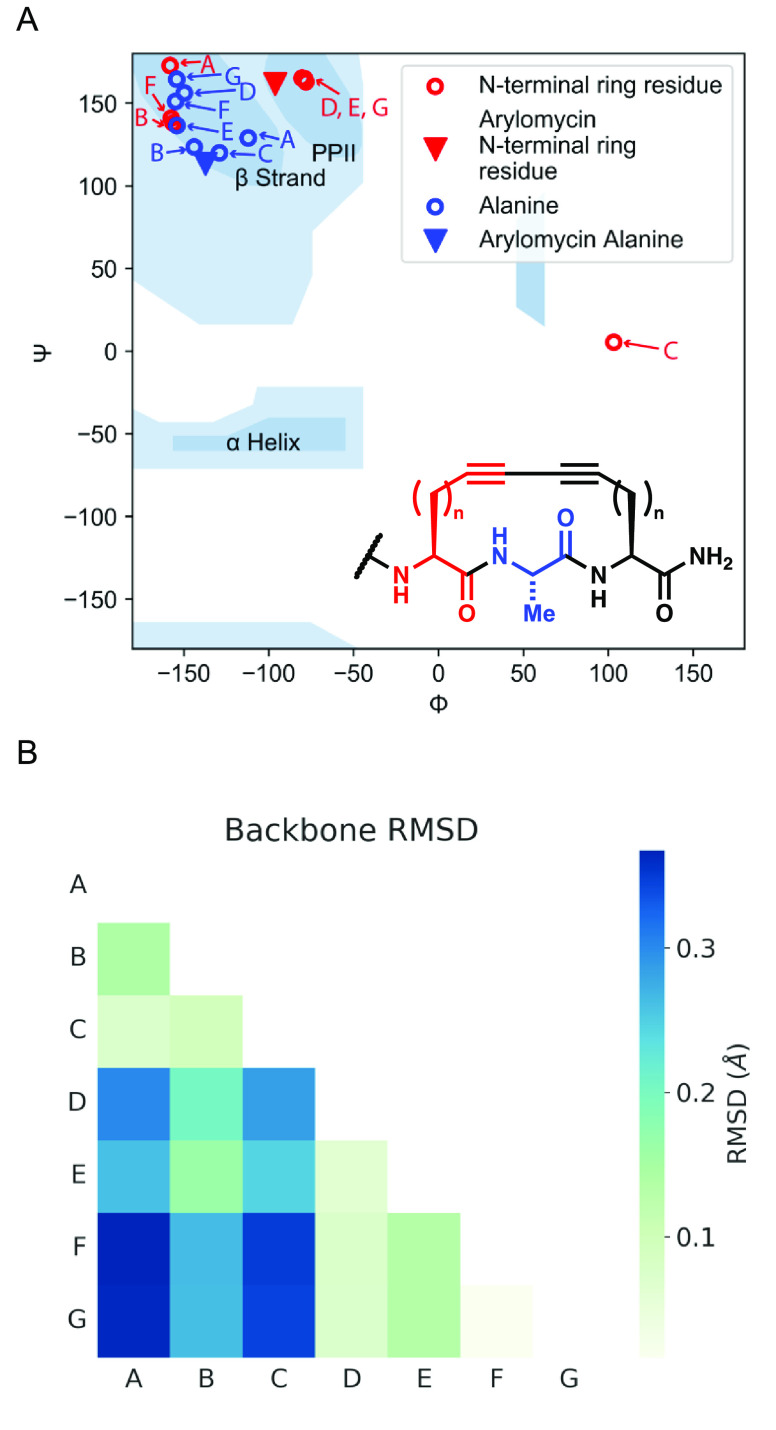
A. Dihedral angles for DFT calculated structures imply a β-strand extended backbone structure. Dihedral angles for arylomycin BAL4850C macrocycle residues depicted by triangles for comparison. B. Pairwise comparison of backbone structure with varied ring type.

All library members exhibited strong evidence of extended backbone conformation. Plotting the dihedral angles of the most abundant DFT calculated structures on a Ramachandran plot shows that the dihedral angles of the restricted residues are clustered in a region that coincides with the most energetically favorable β-strand and PPII helix geometries (Figure 3A, Table S3).^32^ This limited set of seven macrocycles provides substantial coverage of naturally occurring β-strand and PPII helix backbone structures, suggesting that fine-tuning of angles compatible with a broad scope of targets could be obtained by further elaboration of the macrocycle. To further study the effect of these varied rings on the backbone structure, we computed the root-mean-square difference (RMSD) of the DFT macrocycle structures relative to one another (Figure 3B). RMSD values among 13-membered macrocycles were low, supporting that the backbone conformation of these macrocycles is very similar. Interestingly, RMSD values were also low between all 15- and 17-membered macrocycles, implying that larger ring sizes confer a similar distension. These observations are consistent with our prior conclusion that ring size and extended character are correlated. Although ring size is the main predictor of similar backbone conformation, the α-carbon chirality of the ring-forming residues also affects the backbone structure. For example, the RMSD between compounds **B** and **E**, which have the same ring stereochemistry, is comparatively low despite being 13- and 15-membered macrocycles, respectively. Manipulation of both ring size and stereochemistry can be leveraged to design superior structural matches for different target ligands.

Encouraged by the demonstration that diyne-macrocycle compounds have substantial extended-backbone character, determined by both ring size and stereochemistry, we sought to exhibit this principle by applying our platform to a protein target with translational context.

### Application of Diyne Macrocycles

Peptide hydrolases are a large family of enzymes that bind peptide extended conformations. Bacterial type 1 signal peptidase (SPase) is a highly conserved membrane-bound Ser-Lys dyad protease and a validated antibacterial target.^25,26^ SPase uses PPIs to recognize and cleave the N-terminal signal sequence of preproteins translocated across the cytoplasmic membrane.^33−35^ Discovered in 2002,^33,34^ the arylomycins are bacteria-derived lipopeptide latent antibiotics and naturally occurring examples of ligands evolved to fit in the binding pocket of an enzyme that binds extended peptide structures. Arylomycins contain a peptide sequence bridged by a defining biaryl macrocycle that forces the peptide backbone into an extended conformation. Inspired by these natural backbone-stretchers, we used structural clues from the arylomycins to design a set of diyne-braced peptides for SPase inhibition, a class of compounds we affectionately term “alkynomycins.”

The arylomycin family was not initially pursued, despite activity against both Gram-positive and Gram-negative bacteria, due to a narrow spectrum of activity and no activity against ESKAPE pathogens.^33,36^ Renewed interest in these compounds^37^ showed that broad-spectrum activity against bacteria previously resistant to arylomycins can be reinstated via simple chemical derivatization of the lipid tail that renders the resistance-conferring mutation in SPase irrelevant,^38^ leading to recent syntheses of numerous novel potent analogues.^26,37,39−41^

**Figure 4:**
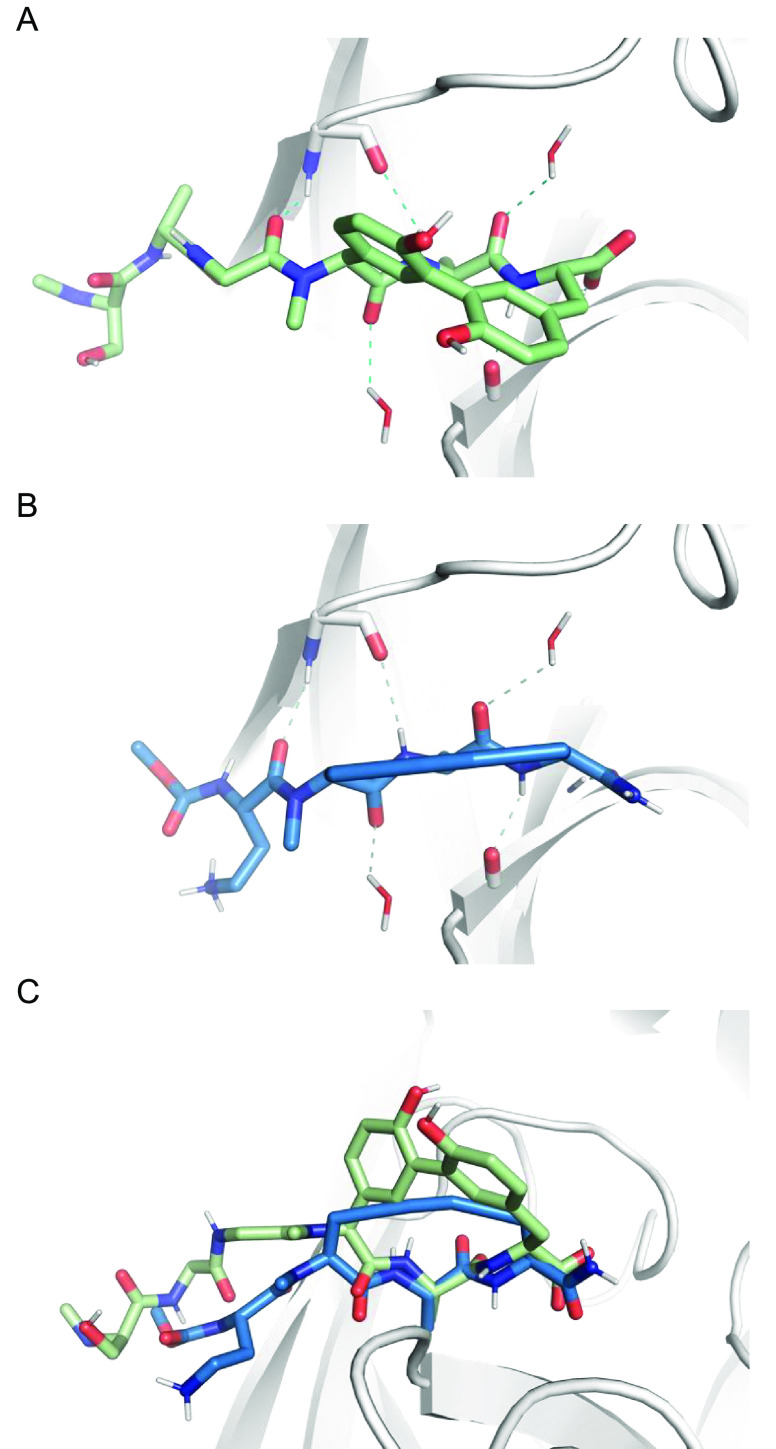
A. Arylomycin A_2_ cocrystallized with *E. coli* type 1 signal peptidase (PDB 1T7D). B. Alkynomycin compound **A** docked in *E. coli* type 1 signal peptidase. C. Superposition of arylomycin A_2_ and alkynomycin compound **A**.

Structural analyses of SPase in complex with arylomycin confirm that the arylomycins bind the SPase class by adopting an extended peptide backbone (Figure 4A).^24^ Since the biaryl moiety is solvent exposed without productive interactions with the highly conserved catalytic pocket, its primary structural role is to preorganize the peptidic SPase-binding motif.^42,43^ A comparison of the dihedral angles in DFT calculated structures of our library of macrocycles with those of arylomycin compound BAL4850C showed good correlation (Figure 3A). Dihedral angles of the two residues constrained by the diyne or arylomycin macrocycle are consistent with a β-strand conformation. Similarly, NMR coupling constants for arylomycin compounds^39^ are comparable to those of the alkynomycins, particularly for the 13-membered diyne-braced rings. Indeed, the puckered backbone conformation of the 13-membered diyne-braced macrocycles is structurally analogous to the native arylomycin backbone.

Using our library of diyne-braced macrocycles and inspiration from the arylomycin class of compounds, a preliminary set of analogues was generated for evaluation in a MIC assay against a panel of Gram-positive and Gram-negative bacteria (Table 2, Table S4). An *N*-methyl was installed on the N-terminal ring residue, as this group has previously been shown to be required for arylomycin activity.^40^ In accordance with previous synthetic optimization, the ring was followed by diaminobutyric acid instead of lysine.^26^ The N-terminus was functionalized with synthetically accessible and commercially available dodecyl- or hexadecyl-carbamate tails selected based on prior arylomycin studies.^44^

**Table 2:**
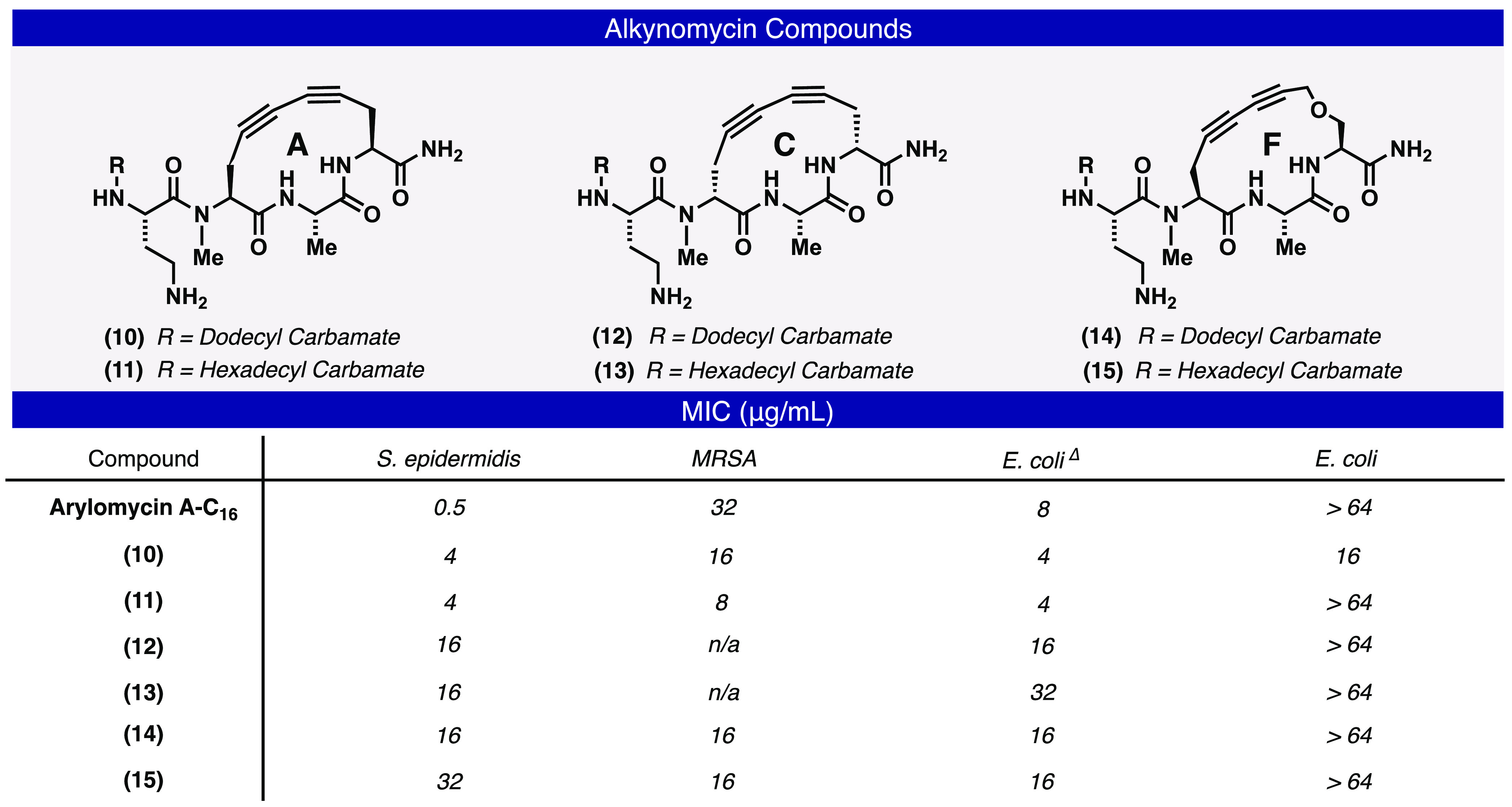
Minimum Inhibitory Concentration of Alkynomycins against Gram-Positive and Gram-Negative Bacteria^a^

a MICs given in μg/mL; performed in triplicate. Strains are *S. epidermidis* RP26a, MRSA USA 300, *E. coli*^Δ^ (*E. coli* BAS901–permeabilized), and *E. coli* MG1655. Previously reported arylomycin A-C_16_ MIC values^38^ provided for reference (structure in Figure 1).

The activities of the most efficacious compounds are summarized in Table 2. Compounds with ring types **A**, **C**, and **F**, having 13-, 13-, and 15-membered macrocycles, respectively, had the highest activity of the alkynomycins tested. Strikingly, without any further optimization, the two alkynomycin compounds with ring type **A** performed comparably (within a factor of 2) to previously reported arylomycin A-C_16_^38^ against MRSA and both permeabilized and nonpermeabilized *E. coli* strains. Despite being less potent than arylomycin A-C_16_ for *S. epidermidis,* both alkynomycins with ring type **A** were still highly potent, and compound **10** even had activity against nonpermeabilized *E. coli.* Consistent with the structure activity relationship of arylomycin, *N-*methylation and macrocyclization were both essential for activity of the alkynomycins, as removal of either of these features ablated activity (Table S5). This dependence of activity on key elements of the arylomycin backbone suggests that the alkynomycins and arylomycins share a common mechanism of action. To further investigate this commonality, a flexible macrocycle docking protocol^45^ was applied to dock a methylcarbamate analogue with ring type **A** against a structure of *E. coli* type 1 signal peptidase cocrystallized with arylomycin A_2_.^24^ Briefly, the macrocyclic ring was broken at the diyne linkage to give a linear peptide. Pseudo atoms with an attractive potential to the opposing carbon were positioned at the end of each resulting terminal alkyne, allowing AutoDock-GPU to sample the conformational states of the macrocycle during docking while guaranteeing ring closure. The results show that the diyne-stretched backbone of alkynomycin **A** docks with a binding mode strikingly similar to the biaryl-stretched backbone of arylomycin A_2_ (Figure 4). The docked amide backbone conformation induced by ring type **A** very closely reproduces the hydrogen bond network of the bound arylomycin, including interactions with bridging waters. Analogous to arylomycin A_2_, the macrocyclic diyne linkage of the bound conformation of alkynomycin **A** is projected out of the pocket, toward solvent, without significant interactions. This further suggests that the role of the macrocyclization is to preorganize the hydrogen bonding moieties of the peptide backbone.

Taken together, these results support that the alkynomycin **A** scaffold can be considered a first-generation lead compound that should benefit from the rich structure-function data that has been collected for the arylomycin scaffolds. The alkynomycin compounds tested mimic the structure of arylomycin A-C_16_ most closely, while studies have already shown that modifications at the C-terminus, the N-terminal lipid, and *N*-methylation sites drastically improve potency. Incorporation of these known modifications, or screening for further modifications at these sites, has the potential to refine the activity of alkynomycin antimicrobials.

Although alkynomycins with ring types **A** and **C** share the same ring size, compounds with ring type **A** had significantly greater potency. Interestingly, alkynomycin **B**, which also has the same ring size as **A** and **C** and significant extended backbone structure suggested by NMR, was inactive (>64 μg/mL). Similarly, despite having the same 15-membered ring size, compounds with ring types **D** and **E** were far less active than ring type **F** compounds, as was the 17-membered macrocycle **G**. These results emphasize the possibility of creating unique ligands that match the requirements of distinctive binding landscapes by varying the identity of the macrocycle. Screening larger libraries of molecules of this class has the potential to uncover precisely tailored fits for a broad array of targets.

**Figure 5:**
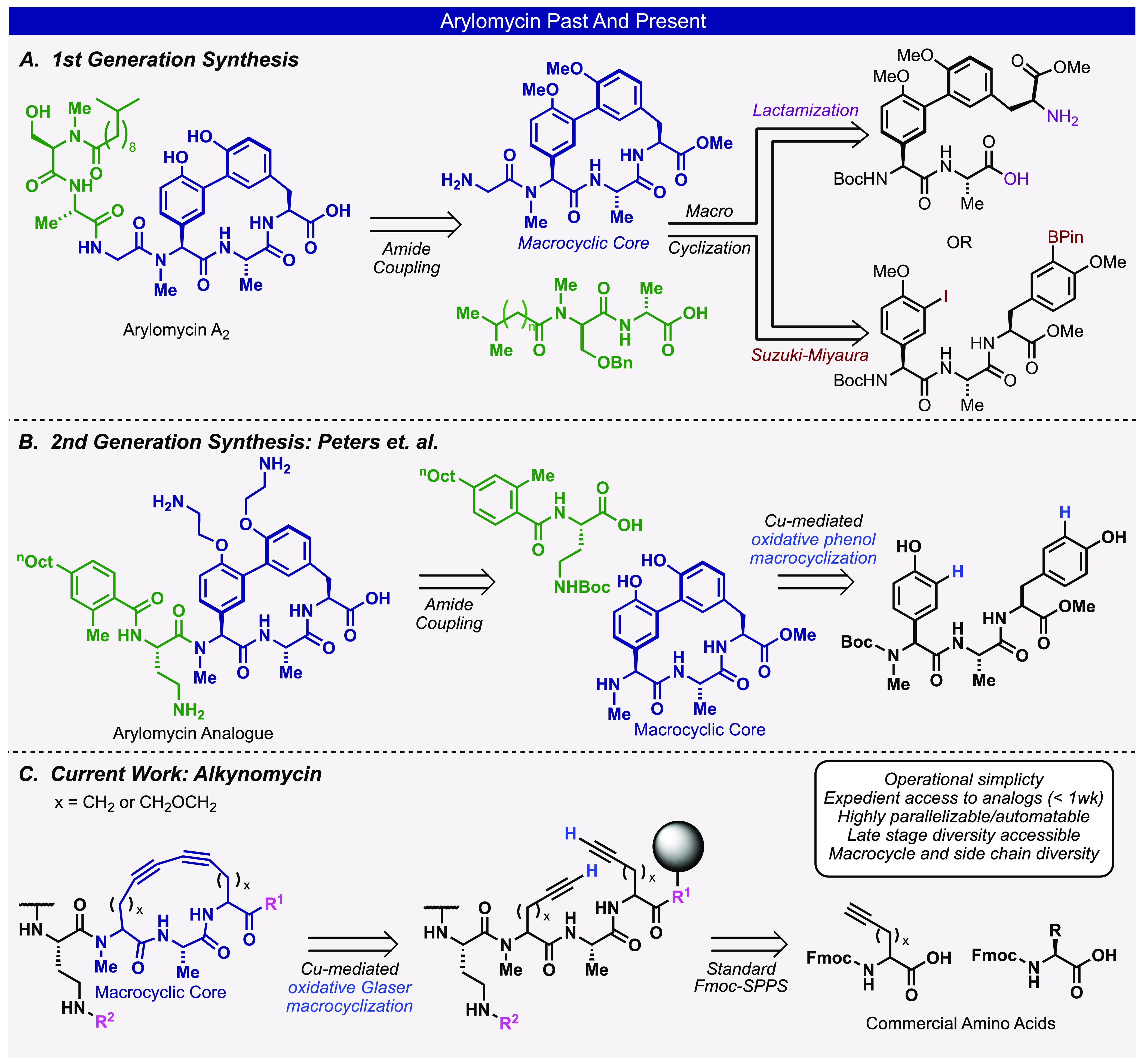
A. First generation synthesis of arylomycin A_2_ via Suzuki-Miyaura macrocylization. B. Second generation synthesis enabling optimization of potent arylomycin analogues via oxidative phenol macrocyclization. C. Current work: synthesis of alkynomycins via on-resin backbone construction and oxidative Glaser macrocyclization.

Access to low molecular weight diyne-braced β-strand mimics with designed fit for target proteins is promising for the development of therapeutics to target the many diseases mediated by PPIs. Synthetic access to diverse analogues and large quantities of natural products is often limited by availability of starting materials, low yields, and difficult chemical steps. Active pursuit of the arylomycins has established viable synthetic routes, Figure 5.^26,37,39−41^ In contrast, diyne-bracing eliminates the synthetically challenging biaryl ring while maintaining the SPase-recognized extended backbone conformation. The solid phase peptide synthesis (SPPS)-compatible route to alkynomycins can enable rapid generation of diverse and efficacious analogues with minimal purification steps.

Antibiotic resistance poses a serious global health risk and contributes to an increasing number of mortalities each year.^46−49^ With developments of new classes of antibiotics stalled in the last decades, the need for new strategies to target bacteria and stave off infection has become pressing. The arylomycins are not the sole example of Nature using peptide stretching for antibiotics. Darobactin, a recently discovered bismacrocyclic heptapeptide antibiotic, is stretched by two intersecting macrocycles into a β-strand structure. This allows it to bind along the exposed face of a β-sheet in its protein target,^50,51^ highlighting a distinct binding modality for stretched peptides. Applying inspiration from the mechanism of action of macrocyclic natural product antibiotics has the potential to uncover new classes of stretched-backbone peptide antibiotics. The present strategy for simplifying synthetic routes to stretched backbone peptides using diyne bracing should be broadly applicable in the development and high-throughput screening of novel efficacious antimicrobials.

## Conclusion

Here we report the efficient on-resin synthesis of a new class of biomimetic compounds characterized by rigid diyne ring systems. The establishment of this method facilitates access to low molecular weight (<300 Da) mimics of β-strand and PPII helix motifs abundant in natural protein-peptide ligand complexes, with minimal chemical perturbations to the peptide sequence. Both theoretical DFT calculations and experimental NMR data indicate significant extended backbone character in these compounds, controlled by ring size and stereochemistry. Using this synthetic control to access different extended structures can enable the creation of molecules with designed fit for the protein of interest. Since this method can be performed on-resin, we foresee this chemistry enabling the efficient generation of chemically diverse libraries. The alkynomycins, a new class of *i, i+2* diyne-braced antimicrobial compounds, illustrate the promise of diyne-braced peptides for new opportunities to modulate PPIs.

Implementation of our diyne peptide-bracing strategy exemplifies its utility in protease inhibitor design to address challenges in human health. Since a significant proportion of the proteome contains extended regions, this strategy for accessing low molecular weight peptide-based extended structures holds great promise for addressing previously “undruggable” targets. Additionally, the diyne bond is inherently Raman sensitive^52^ and can be reacted to form further functionalized analogues.^53,54^ We anticipate the broad application of this method since it is both compatible with standard SPPS and drug discovery methods and consistent with inspiration from natural products like arylomycin and darobactin, where the mechanism of action is driven by the binding conformation of the peptide backbone.^33,34,51^ Stretching peptides via diyne-bracing promises to open doors to enable the modulation of previously difficult-to-target interactions between proteins.

## Supporting Information Available

Materials, methods, structural, biological, and analytical data (PDF)
