# Supplementary material for: Stretching Peptides to Generate Small Molecule β-Strand Mimics
Source: ACS Cent Sci. 2023 Mar 15;9(4):648–56. doi: 10.1021/acscentsci.2c01462 (PMC10141592; doi:10.1021/acscentsci.2c01462)
Supplement: Supplementary file 1 — oc2c01462_si_001.pdf [file oc2c01462_si_001.pdf]

Supporting Information

Stretching peptides to generate small molecule β-strand mimics

*Zoë C. Adams,‡ Anthony P. Silvestri,‡ Sorina Chiorean, Dillon T. Flood, Brian P. Balo, Yifan Shi, Matthew Holcomb, Shawn I. Walsh, Colleen A. Maillie, Gregory K. Pierens, Stefano Forli, K. Johan Rosengren, and Philip E. Dawson**

‡ Co-first author
* Corresponding author (dawson@scripps.edu)

Table of Contents

Materials ***2***

General Methods ***2***

Synthetic Methods **2**

General Solid-Phase Peptide Synthesis (SPPS) Procedure 2
N-Methylation 2
On-Resin Glaser Coupling 2
Chloroformate Coupling 2
Cleavage and Purification 2
Safety Statement 2

Analytical and Purification Conditions **2**

Nuclear Magnetic Resonance Spectroscopy **3**

Density Functional Theorem Modeling **4**

Minimum Inhibitory Concentration Assay **4**

Molecular Docking **4**

Supporting Figures ***5***

Analytical Peptide Data ***8***

Glaser Coupling Optimization **8**

Diyne-Braced Model Peptides **39**

Alkynomycin Compounds **53**

References ***82***

Materials

All reagents were obtained directly from commercial suppliers and used without further purification. N,N-dimethylformamide (DMF), dichloromethane (DCM), N,N-diisopropylethylamine (DIPEA), trifluoroacetic acid (TFA), N,N-dimethylacetamide (DMA), dimethyl sulfoxide (DMSO), sodium borohydride ($NaBH_4$), and Rink-Amide Polystyrene Resin (loading capacity 0.64 mmol/g) from Millipore-Sigma; 2,2'Bipyridine, diethyl ether, acetonitrile (MeCN), acetic anhydride, 4-methylpiperidine, 1,8-Diazabicyclo[5.4.0]undec-7-ene (DBU), ethyl trifluoroacetate (EtTFA), diisopropyl azodicarboxylate (DIAD), and 4-dimethylaminopyridine (DMAP) from Fisher Scientific; Methanol (MeOH, dry over molecular sieves), dodecyl chloroformate, and hexadecyl chloroformate from VWR Intl; HATU (1-[Bis(dimethylamino)methylene]-1H-1,2,3-triazolo[4,5-b]pyridinium 3-oxide hexafluorophosphate) and triisopropylsilane (TIPS) from Oakwood Products Inc.; Fmoc-Arg(Pbf)-OH, Fmoc-Ala-OH, Fmoc-Tyr(tBu)-OH, and Fmoc-Leu-OH from Bachem. Fmoc-Pra-OH, Fmoc-Prs-OH, and triphenylphosphine (PPh_3) from CombiBlocks; Fmoc-Dab(Boc)-OH from ChemScene LLC; Copper (I) chloride from Strem Chemicals, Inc.; Ninhydrin test kit from Anaspec Inc.; DMSO-d_6 and D_2O from Cambridge Isotope Labs. Water was purified using a Millipore Milli-Q Q-Gard 2 water purification system to a resistivity of 18.2 MΩ*cm.

General Methods

Synthetic Methods

General Solid-Phase Peptide Synthesis (SPPS) Procedure All peptides were chain assembled on Rink Amide polystyrene resin (0.64 mmol/g) or Rink-Amide TentaGel resin (0.2 mmol/g, Rapp Polymere GmbH) by individual hand couplings. All standard amino acid couplings were carried out with the equivalent ratio of [5]:[5]:[7.5] of [Fmoc-protected amino acid]:[0.4 M HATU in DMF]:[DIPEA] for 20 min following standard SPPS protocol with N-terminal Fmoc-protection. Propargyl containing amino acids were coupled by hand using the equivalent ratio of [2.5]:[2.5]:[3.8] of these components for 90 min, and followed by a qualitative ninhydrin test to ensure complete coupling.

N-Methylation After peptide assembly of the macrocycle ring, *N*-methylation of residue 3 was performed on-resin. Resin was treated twice with [1]:[1] DBU:EtTFA in DMF for 30 min, and then checked with a qualitative ninhydrin test to ensure complete protection of the N-terminus. An equivalent ratio of [5]:[10] [PPh_3]:[MeOH] in DCM was added to resin in a closed tube with stopcock. 5 equivalents DIAD was added last, and the tube was vented, then shaken for 1 hr. This procedure was repeated until a test cleavage showed good conversion to the methylated N-terminus. The protecting group was cleaved with 20 equivalents of $NaBH_4$ in 1:1 MeOH:DCM. The amino acid directly following this step was coupled twice by standard SPPS protocol.

On-Resin Glaser Coupling To a 15 mL Falcon tube was added CuCl (white powder, stored under Ar, 100 mg, 1 mmol, 10 equiv.), 2,2'-bipyridine ligand (165 mg, 1.5 mmol, 15 equiv.), and 0.1 mmol of washed Fmoc-deprotected peptide-loaded resin, prior to the addition of any alkyl tails. The mixture was briefly vortexed to mix the Cu and ligand. To this tube was added 5 mL DMA, followed by 175 µL DIPEA (1 mmol, 10 equiv.). The tube was capped then parafilmed and placed horizontally in a secondary container to reduce settling of compounds to the bottom of the tube and improve interaction of the solution with O_2 in the headspace. The tube was incubated at 37 C with shaking at 180 rpm for 5 hr. Following the stapling reaction, the resin was washed with DMF and 20% piperidine and subjected to either continued synthesis (i.e. coupling of alkyl tail) or standard TFA cleavage conditions for SPPS procedure.

Chloroformate Coupling Alkyl chloroformate tails were appended by adding DMAP (1 equiv.) to a mixture of resin and chloroformate (5 equiv.) in 1:1 DCM:DMF (2 mL total), stirring for 1 hr, and subsequent flow washing with DMF. Coupling completion was ensured by a qualitative ninhydrin test.

Cleavage and Purification Peptides were cleaved from resin within 48 hr of the formation of the diyne brace to prevent undesired copper-catalyzed modifications observed to occur on-resin. Peptides proceeded to standard cleavage from resin using [2.5]:[2.5]:[95] TIPS:H2O:conc. TFA at 45 C for 4 hr. The TFA was reduced via rotovap then added to an excess of 30% MeCN and lyophilized. The crude peptide thus obtained was purified via preparative reverse-phase HPLC using optimized gradients as detailed in the analytical peptide data.

Safety Statement No unexpected or unusually high safety hazards were encountered.

Analytical and Purification Conditions

Crude peptides were purified on preparative HPLC (Waters Autopurify prep LC with diode array and QDa mass spec) using either the generic gradient or an optimized gradient as specified. Peptides were characterized by analytical LC/MS on a Waters Acquity I-Class UPLC with diode array and time of flight mass spec (Waters G2-XS).

Waters Generic Preparative HPLC Mobile Phase and Gradient:
Buffer A: H_2O (0.1% TFA)
Buffer B: MeCN

Initial:	10% B
1.0 minute:	10% B
9.0 minutes:	25% B
9.5 minutes:	95% B
12.0 minutes:	10% B

Waters Analytical LC/MS Mobile Phase and Gradient:
Buffer A: H_2O (0.1% Formic Acid)
Buffer B: MeCN

Initial:	5% B
0.4 minutes:	5% B
5.0 minutes:	99% B
5.4 minutes:	5% B

Purity and identity of peptides was confirmed using a Waters I-Class LC with diode array and G2-XS time of flight (TOF) mass spectrometer with the analytical gradient listed above.

Nuclear Magnetic Resonance Spectroscopy

Two main approaches were taken when preparing the braced peptide products for NMR analyses. Braced peptides not containing a lipid tail (0.8 – 1.4 mg) were dissolved in 450 µL H_2O and 50 µL D_2O and analyzed in 5 mm tubes on a 700 MHz Bruker Avance III spectrometer equipped with a cryoprobe. Alkynomycin analogues (1.8 – 2.2 mg) were dissolved in 150 µL DMSO-d_6 and analyzed on a 600 MHz Bruker spectrometer equipped with either a 5 mm CPQCI or CPDCH cryoprobe in 3 mm tubes. As all compounds were purified as TFA salts, pH of solutions were adjusted to within the range 4.6 – 5.3 and scans were obtained at 298 K. Water suppression was achieved by excitation sculpting during proton spectral acquisition.

^{1}H homonuclear data included 2D total correlation spectroscopy (TOCSY) with a mixing time of 80 ms, rotating-frame Overhouser effect spectroscopy (ROESY) with a mixing time of 100 ms, and nuclear Overhauser effect spectroscopy (NOESY) with a mixing time of 300 ms. The homonuclear data were recorded with a sweep width of 10 or 12 ppm with 4k data points in the direct and 512 increments in the indirect dimension. Heteronuclear single quantum coherence (HSQC) data were also recorded at natural abundance. The ^{1}H-^{13}C HSQC spectra were recorded with 2k data points over a sweep width of 10 or 12 ppm in the direct dimension, and 280 increments over a sweep width of 80 ppm, covering the aliphatic carbon region, in the indirect dimension. The ^{1}H-^{15}N HSQC spectra were recorded with 2k data points over a sweep width of 10 ppm in the direct dimension, and 128 increments over a sweep width of 32 ppm in the indirect dimension for the group of peptides.

All data were collected using Topspin 4.0.6 (Bruker), processed with MestReNova 14.2.1 (Maestrelab Research). Water solvated samples were referenced to internal DSS at 0.0 ppm and DMSO samples were referenced to residual solvent peak at 2.50 ppm.

Density Functional Theorem Modeling

A Monte Carlo conformational search undertaken using the OPLSe3 forcefield and simulated water solvent (GB/SA) using Macromodel v12.[1] The selected conformers (<3 kcal/mol of the global minimum) were optimized by density functional theory (DFT) calculations at the B3LYP/6-31G(d,p) level with PCM implicit solvent model for water using Gaussian software G16W.[2] A single point energy of the optimized conformers was calculated using M062X/6-31+g(d,p) with PCM implicit solvent model for water and duplicate conformers and conformers with >3 kcal/mol of the global minimum were removed. Finally, single point free energy of the optimized conformers was calculated using M062X/6-31+G(d,p) with PCM implicit solvent model for water were used to scale the calculated NMR parameters relative to their Boltzmann population and the vibrational frequencies where checked for a true minimum, i.e. no negative frequencies and this energy was used to calculated the Boltzmann populations for each compound.

Cutdown Compound	# of conformers from MacroModel (<3kcal/mol)	# of unique conformers after Gaussian optimization (<3kcal/mol)	# of conformers to account for >90% of population
A	16	5	3
B	23	8	2
C	17	11	3
D	48	8	5
E	76	23	8
F	22	6	3
G	12	11	6

Minimum Inhibitory Concentration Assay

MICs were performed in accordance with the broth microdilution protocol from the Clinical and Standards Laboratory Institute.[3] Alkynomycins were dissolved at 10 mg/mL in DMSO and two-fold serial dilutions were made across a 96-well plate containing Muller-Hinton (MH) broth. One plate was used for each indicator organism, and each well reached a final inoculum of 5×10^5 colony forming units per mL. Using OD_{600} readings normalized to a negative control, MICs were recorded as the lowest concentration at which growth was inhibited after a 24 hr incubation at 37 C. Controls were included on each plate to ensure peptide and media sterility. MICs are reported in **Table 2** and **Tables S4, S5** as an average of at least three independent trials.

Indicator organisms were stored at -80 C as glycerol stocks. Each organism was streaked onto tryptic soy agar and grown for 24 hr at 37 C. Single colonies were picked and used to inoculate MH broth. Bacteria culture was diluted to reach an OD_{600} of 0.015 prior to being used for MIC assays.

Molecular Docking

The co-crystallized ligand was removed from the structure (1t7d),[4] with crystallographic waters retained. The structure was prepared for docking using AutoDockTools. Grid maps were prepared using AutoGrid v4.2.6 with 30 Å in each dimension and 0.375 Å spacing. The ligand was prepared with terminal alkynes. "Glue" atoms (G0 type) appropriate for macrocyclic docking were placed 1.5 Å from the end of each of alkyne, and the terminal carbon of the alkynes were typed CG0. Docking was performed using AutoDock-GPU v1.5.3. 20 independent genetic algorithm runs were performed, with an AutoStop triggered by an energy standard deviation cutoff at 0.15 kcal/mol. The best scored pose was used for structural analysis.

Supporting Figures

Table S1. Bond angles and calculation of nonlinearity.

Molecule	Bond Angle 1	Bond Angle 2	Bond Angle 3	Bond Angle 4	Difference From 180 over 4 bonds
**A**	165.7	167.9	169.0	165.0	52.4
**B**	165.6	170.7	169.6	167.8	46.3
**C**	162.3	170.8	168.6	168.4	49.9
**D**	170.1	176.4	175.6	171.6	26.3
**E**	166.3	175.1	173.2	171.3	34.1
**F**	172.8	175.9	177.0	171.4	22.9
**G**	175.2	179.1	179.3	176.5	9.9

For reference, bond angles labeled on diyne of ring type A

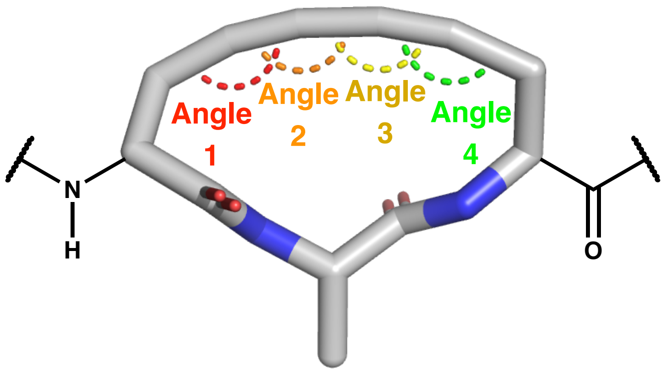

Table S2. Distance between residues measured by α- and β-carbon distances.

Molecule	Distance between α-carbons	Distance between β-carbons
**A**	6.6	6.4
**B**	6.7	6.4
**C**	6.6	6.4
**D**	7.0	7.0
**E**	7.0	6.5
**F**	7.0	7.1
**G**	7.0	8.0

For reference, Structure of Arylomycin A-C16:

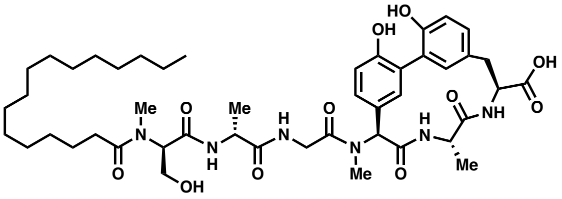

Table S3. Dihedral angles of DFT calculated structures

Compound	Phi N	Phi M	Phi C	Psi N	Psi M	Psi C
**A**	-158.1	-112.1	-124	172.8	129	15.2
**B**	-156.3	-143.9	-71.2	138	123.5	-31.3
**C**	103.2	-129	-70.5	5.4	120	-31.2
**D**	-78.8	-149.7	-130.8	164.8	156.2	20.5
**E**	-78.1	-154.1	-71.9	163	136.5	-31.5
**F**	-157.4	-154.9	-163.5	141	151.1	170
**G**	-80.4	-154.2	-75.9	165.2	164.5	-24.3
**Arylomycin**	-96.3	-137.3	-82.5	161.7	113.4	-38.1

Note: Arylomycin refers to angles from crystal structure of Arylomycin BAL4850C. Angles are denoted as N (N-terminal residue), M (middle residue), or C (C-terminal residue).

Table S4: Minimum inhibitory concentration of alkynomycins against gram-positive and gram-negative bacteria.

Alkynomycin Compounds

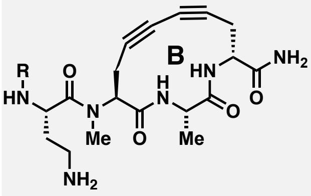

(S1) *R = Dodecyl Carbamate*
(S2) *R = Hexadecyl Carbamate*

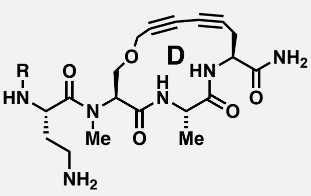

(S3) *R = Dodecyl Carbamate*
(S4) *R = Hexadecyl Carbamate*

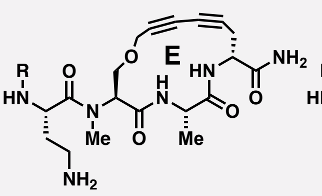

(S5) *R = Dodecyl Carbamate*
(S6) *R = Hexadecyl Carbamate*

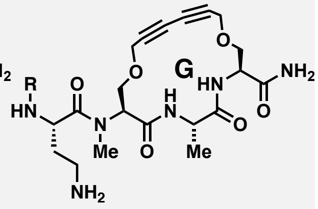

(S7) *R = Dodecyl Carbamate*
(S8) *R = Hexadecyl Carbamate*

MIC (µg/mL)

Compound	*S. epidermidis*	*E. coli* $^{\Delta}$	*E. coli*
**Arylomycin A-C_{16}**	*0.5*	*8*	*> 64*
**(S1)**	*> 64*	*> 64*	*> 64*
**(S2)**	*> 64*	*> 64*	*> 64*
**(S3)**	*> 64*	*> 64*	*> 64*
**(S4)**	*64*	*64*	*> 64*
**(S5)**	*> 64*	*> 64*	*> 64*
**(S6)**	*64*	*64*	*> 64*
**(S7)**	*> 64*	*> 64*	*> 64*
**(S8)**	*64*	*64*	*> 64*

MICs given in *u*g/mL; performed in triplicate. Strains are *S. epidermidis* RP26a, MRSA USA 300, *E. coli* BAS901 (perm.), and *E. coli* MG1655

Table S5: Minimum inhibitory concentration of alkynomycins missing the *N*-methyl or Glaser brace against gram-positive and gram-negative bacteria.

N-Methyl and Glaser Brace are Required for Activity in Alkynomycin Compounds

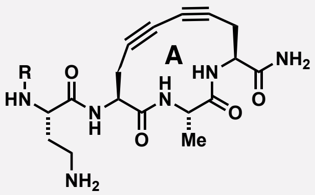

(S9) *R = Dodecyl Carbamate*
- *N*-methyl

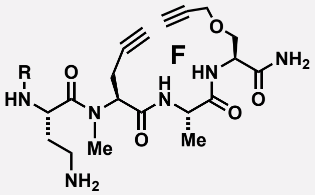

(S10) *R = Dodecyl Carbamate*
- Glaser Brace

MIC (µg/mL)

Compound	*S. epidermidis*	*E. coli* $^{\Delta}$	*E. coli*
**(S9)**	*> 64*	*> 64*	*> 64*
**(S10)**	*> 64*	*> 64*	*> 64*

MICs given in *u*g/mL; performed in triplicate. Strains are *S. epidermidis* RP26a, MRSA USA 300, *E. coli* BAS901 (perm.), and *E. coli* MG1655

Analytical Peptide Data

Glaser Coupling Optimization

<u>Compounds 1, 2: Test Peptides</u>

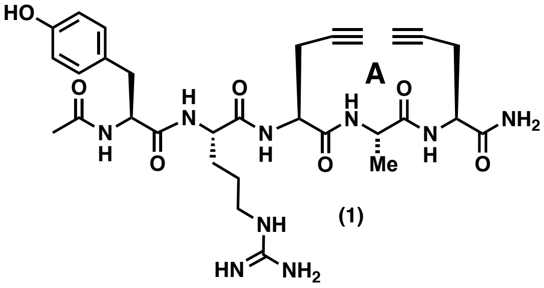

(1)

Chemical Formula: $C_{30}H_{41}N_9O_7$

Molecular Weight: 639.71

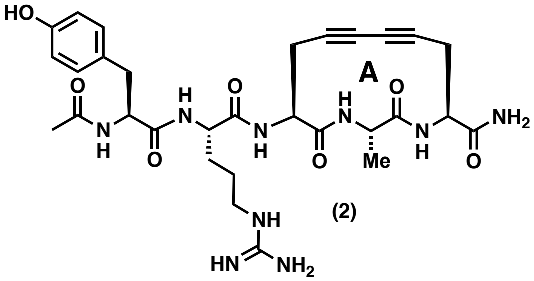

(2)

Chemical Formula: $C_{30}H_{39}N_9O_7$

Molecular Weight: 637.70

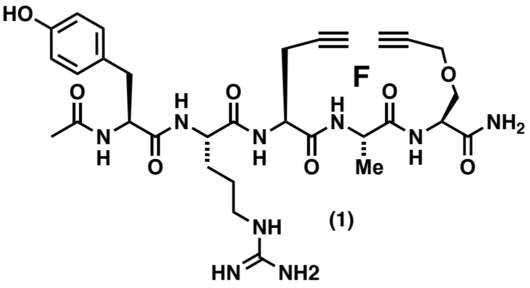

(1)

Chemical Formula: $C_{31}H_{43}N_9O_8$

Molecular Weight: 669.74

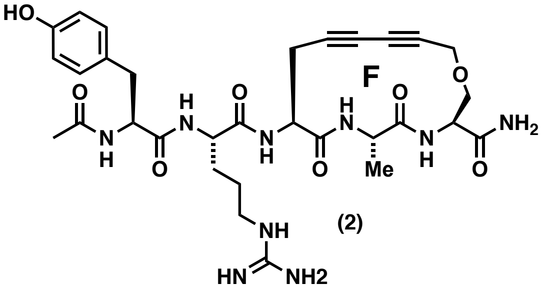

(2)

Chemical Formula: $C_{31}H_{41}N_9O_8$

Molecular Weight: 667.72

Glaser Coupling Conditions: Unbraced Pra-Ala-Pra

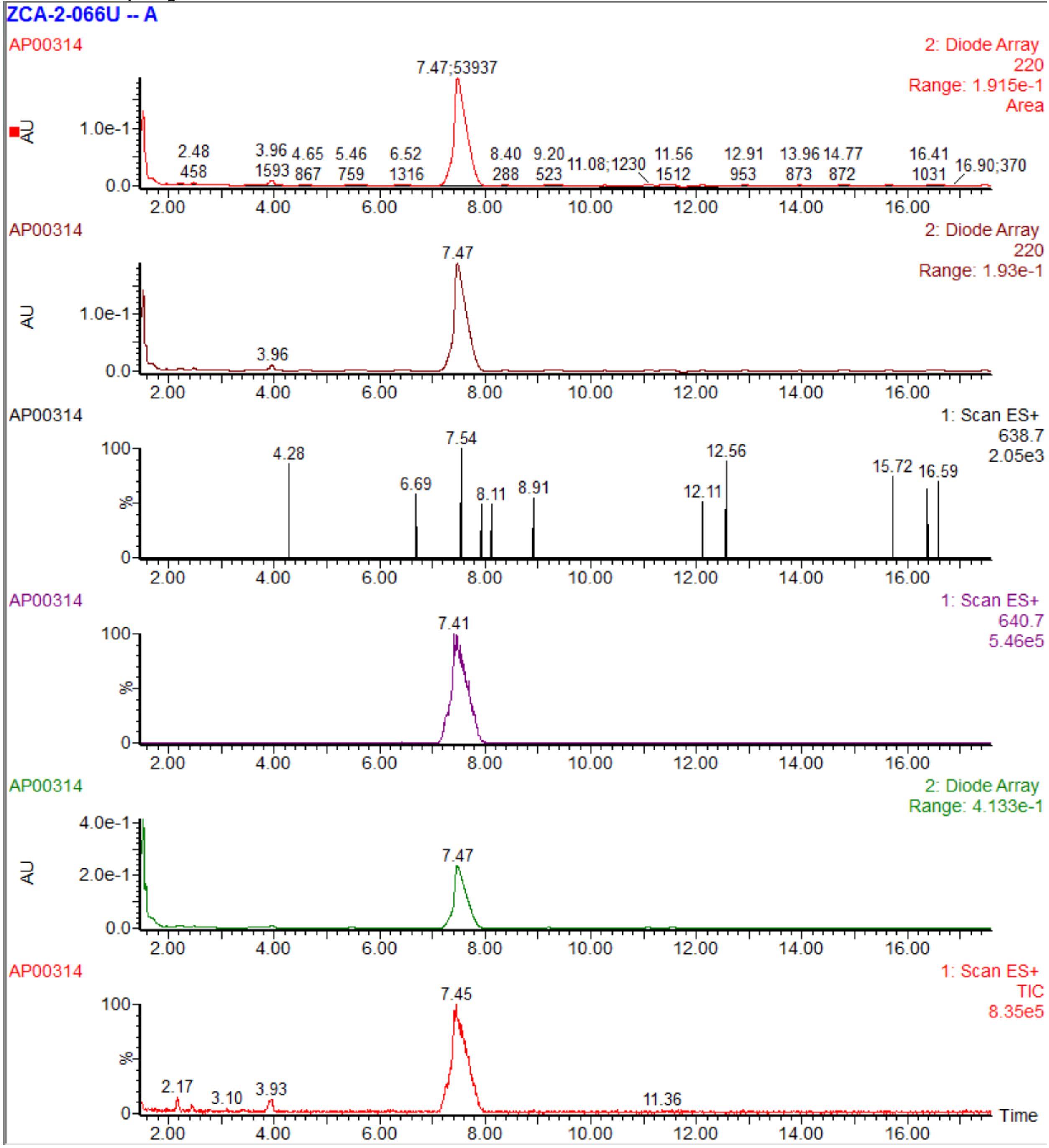

Glaser Coupling Conditions: Unbraced Pra-Ala-Prs

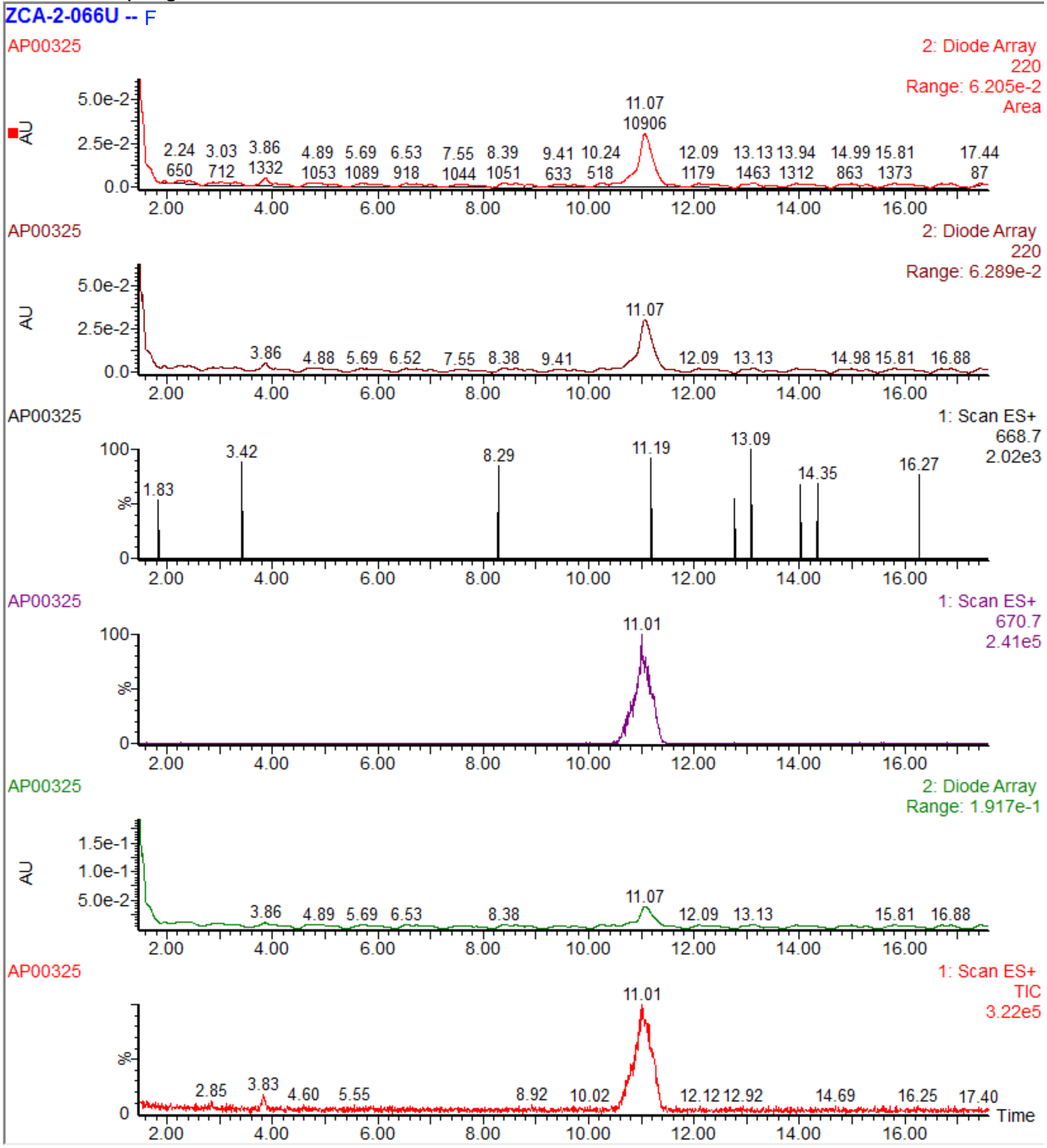

Glaser Coupling Conditions: Pra-Ala-Pra, DMSO (anhydrous), 5 h

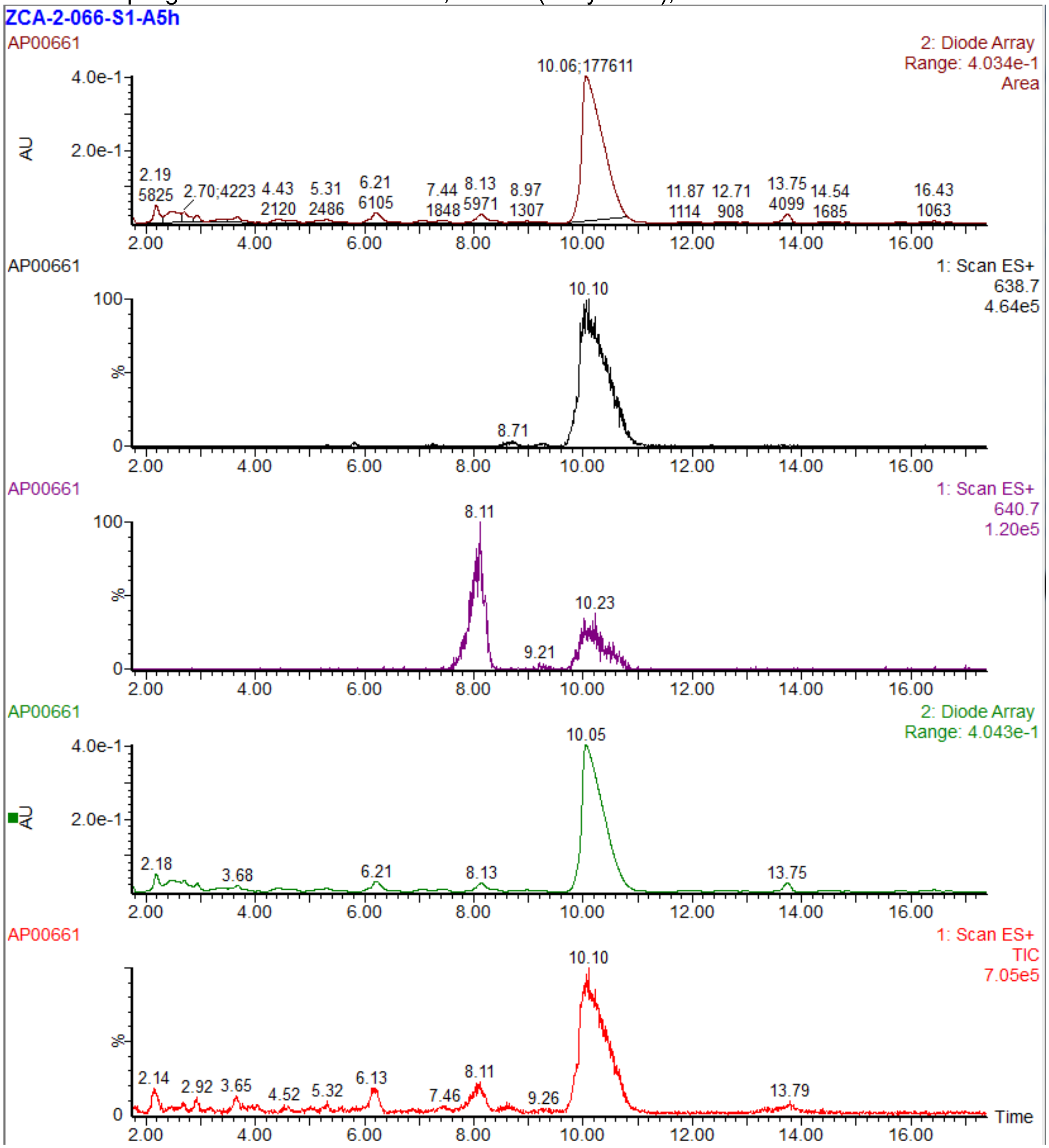

Glaser Coupling Conditions: Pra-Ala-Prs, DMSO (anhydrous), 5 h

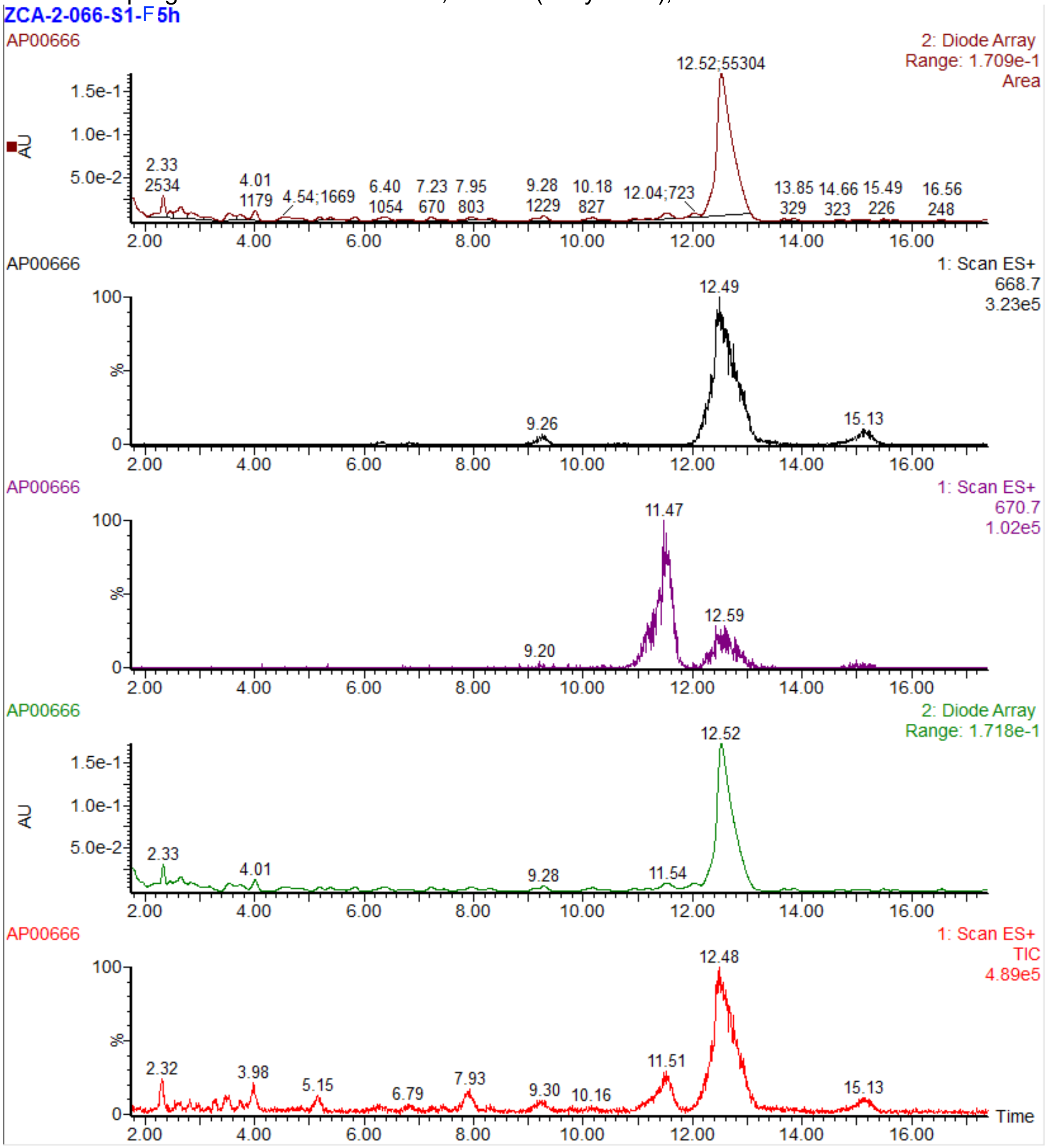

Glaser Coupling Conditions: Pra-Ala-Pra, DMSO, 5 h

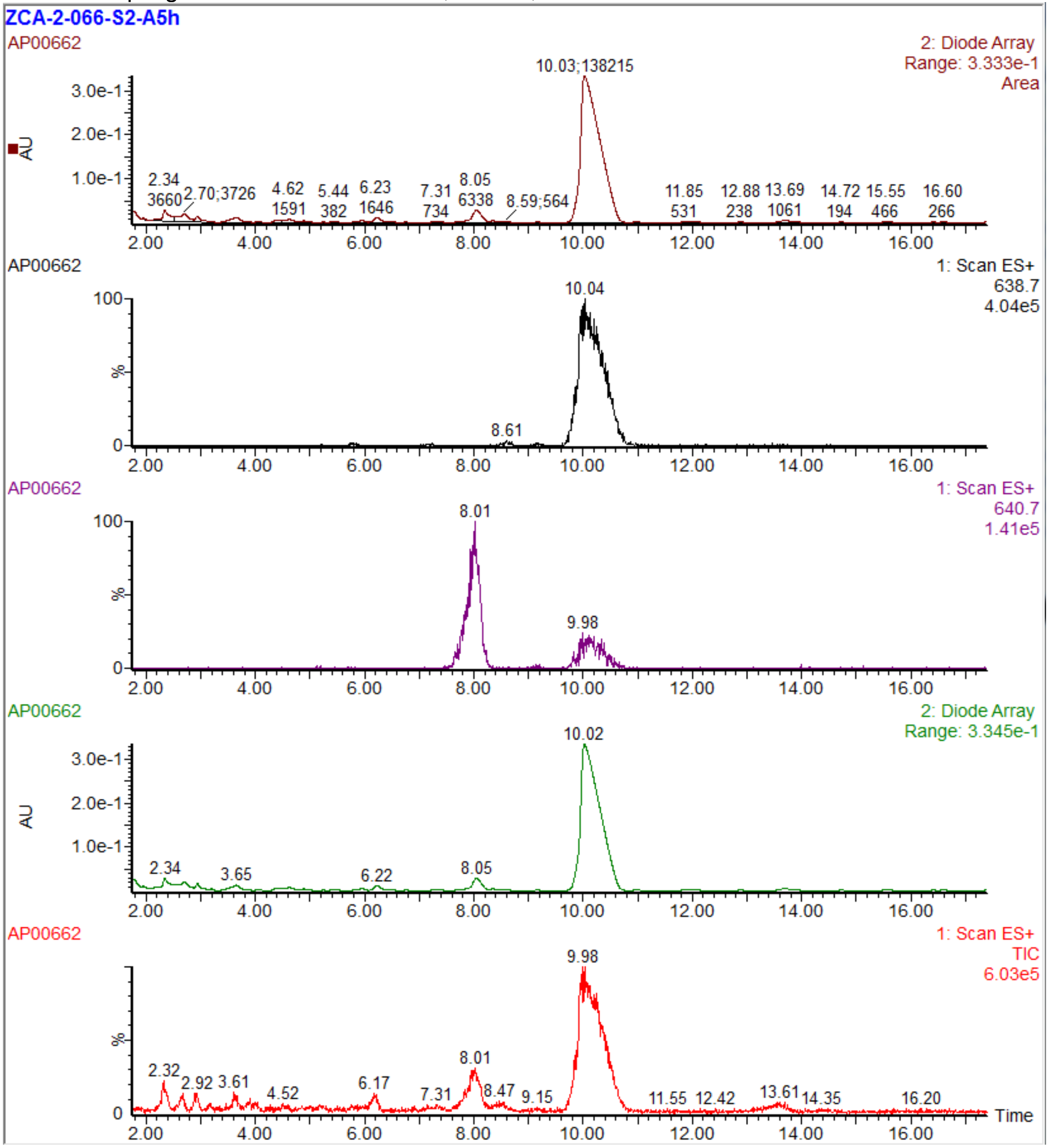

Glaser Coupling Conditions: Pra-Ala-Prs, DMSO, 5 h

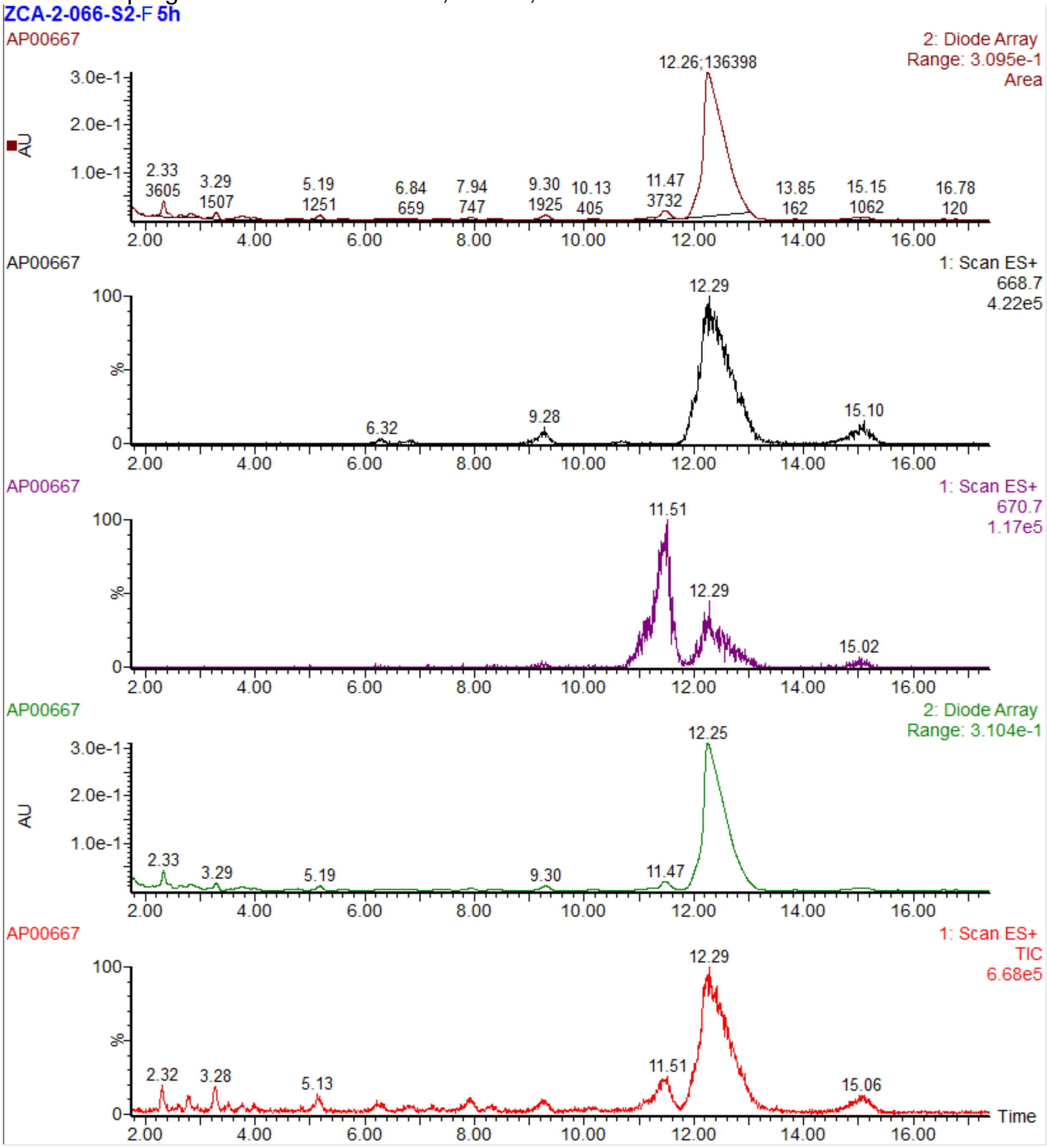

Glaser Coupling Conditions: Pra-Ala-Pra, DMA, 5 h

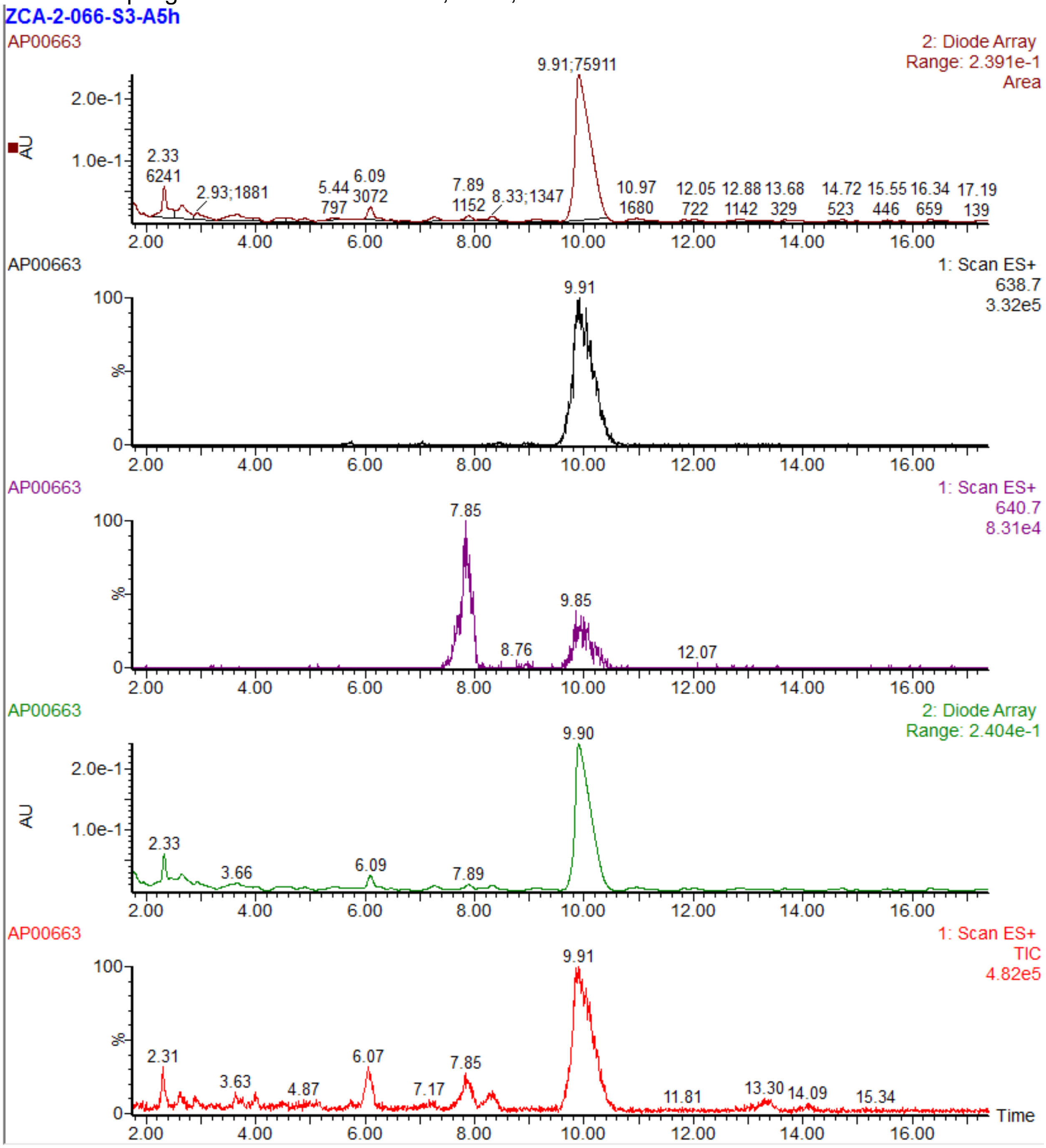

Glaser Coupling Conditions: Pra-Ala-Prs, DMA, 5 h

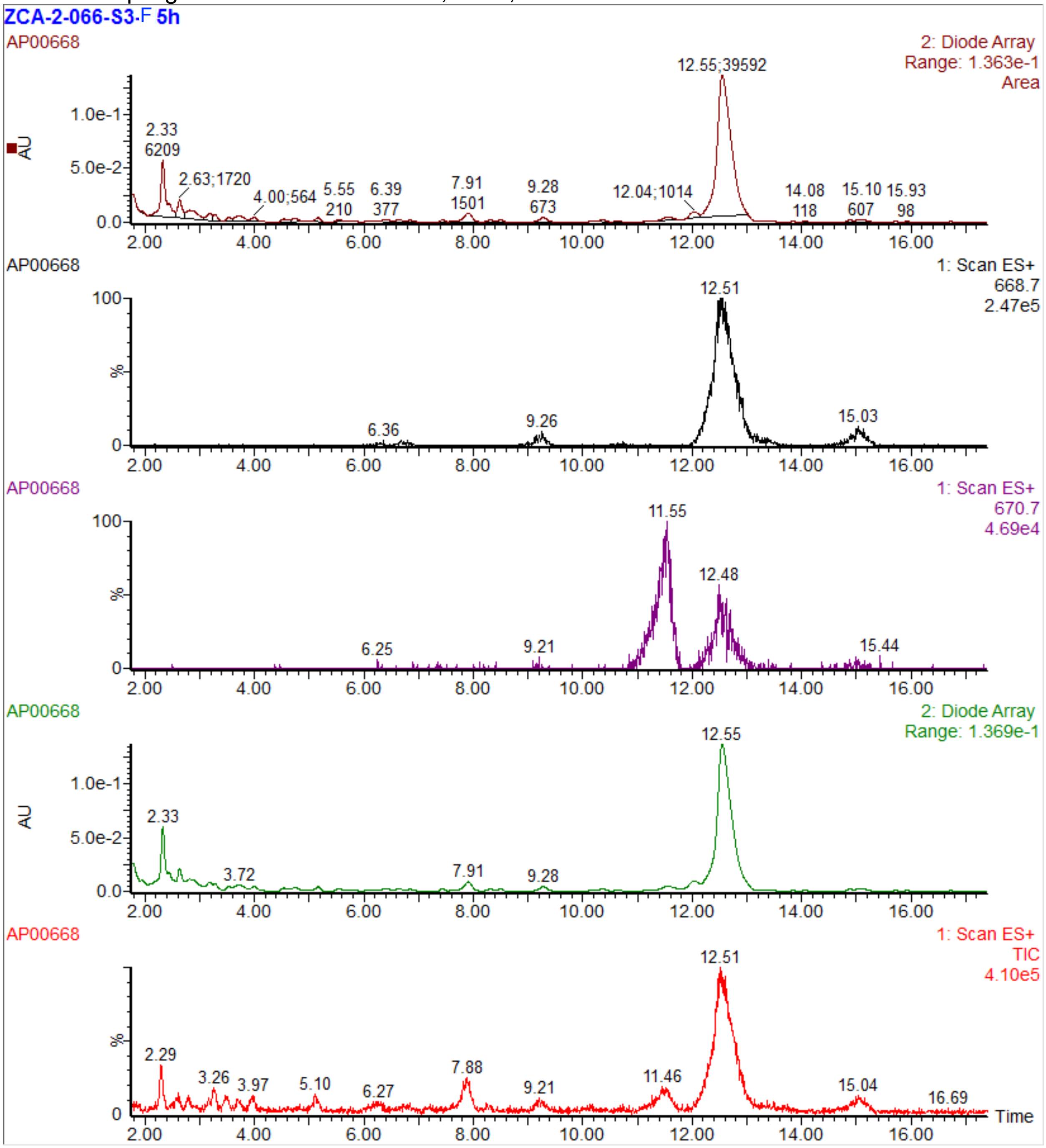

Glaser Coupling Conditions: Pra-Ala-Pra, DMF, 5 h

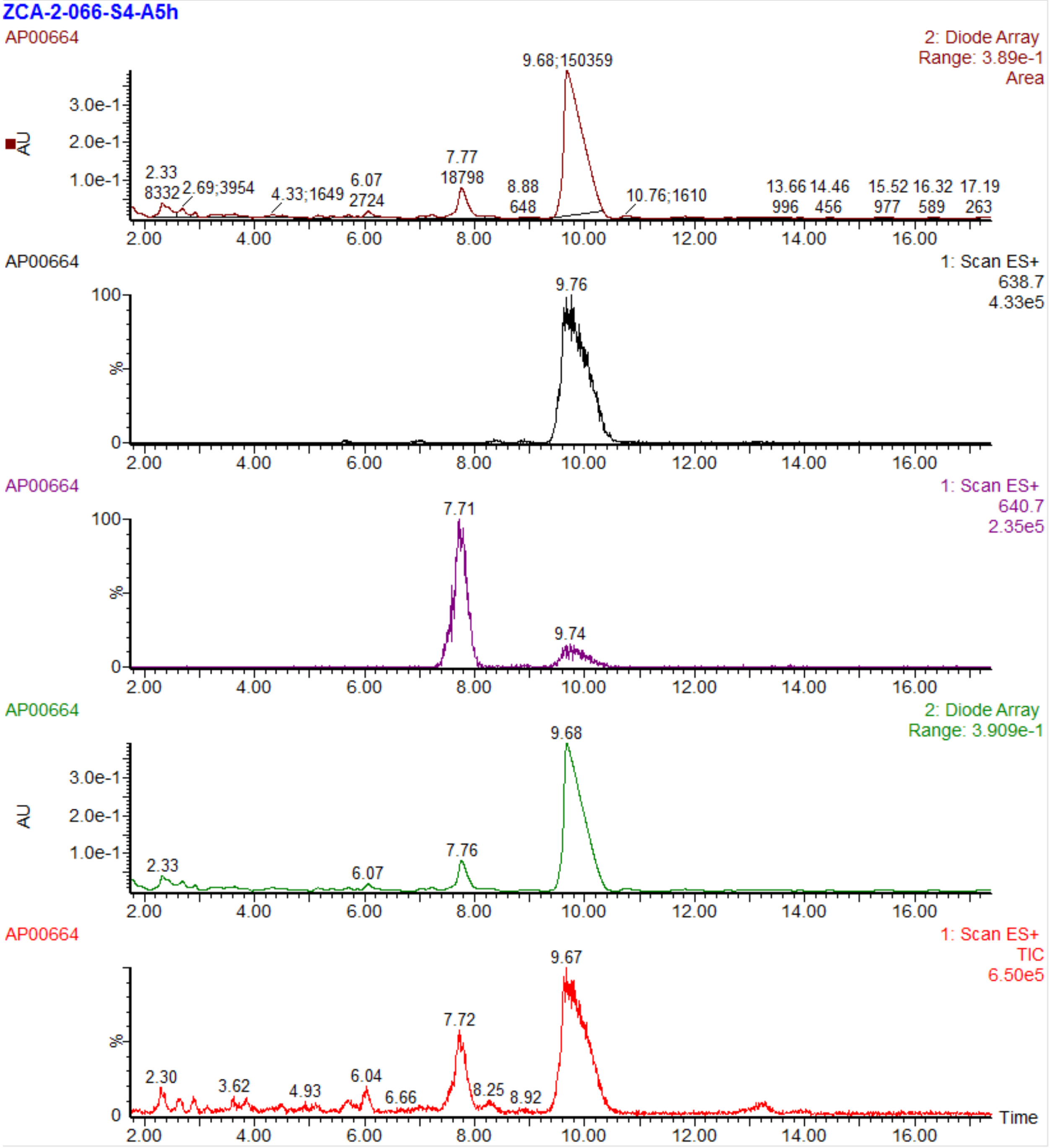

Glaser Coupling Conditions: Pra-Ala-Prs, DMF, 5 h

ZCA-2-066-S4-F 5h

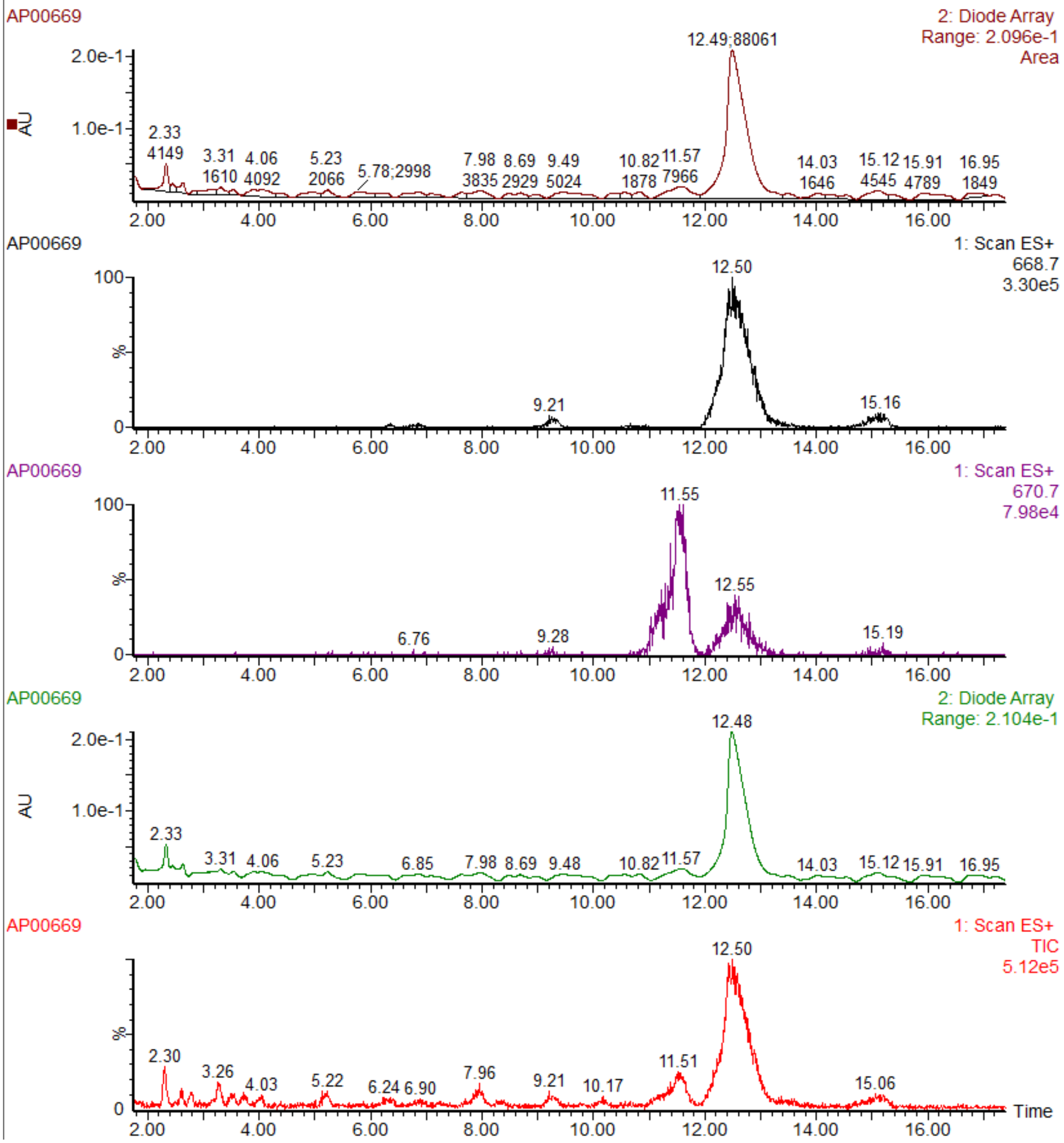

Glaser Coupling Conditions: Pra-Ala-Pra, NMP, 5 h

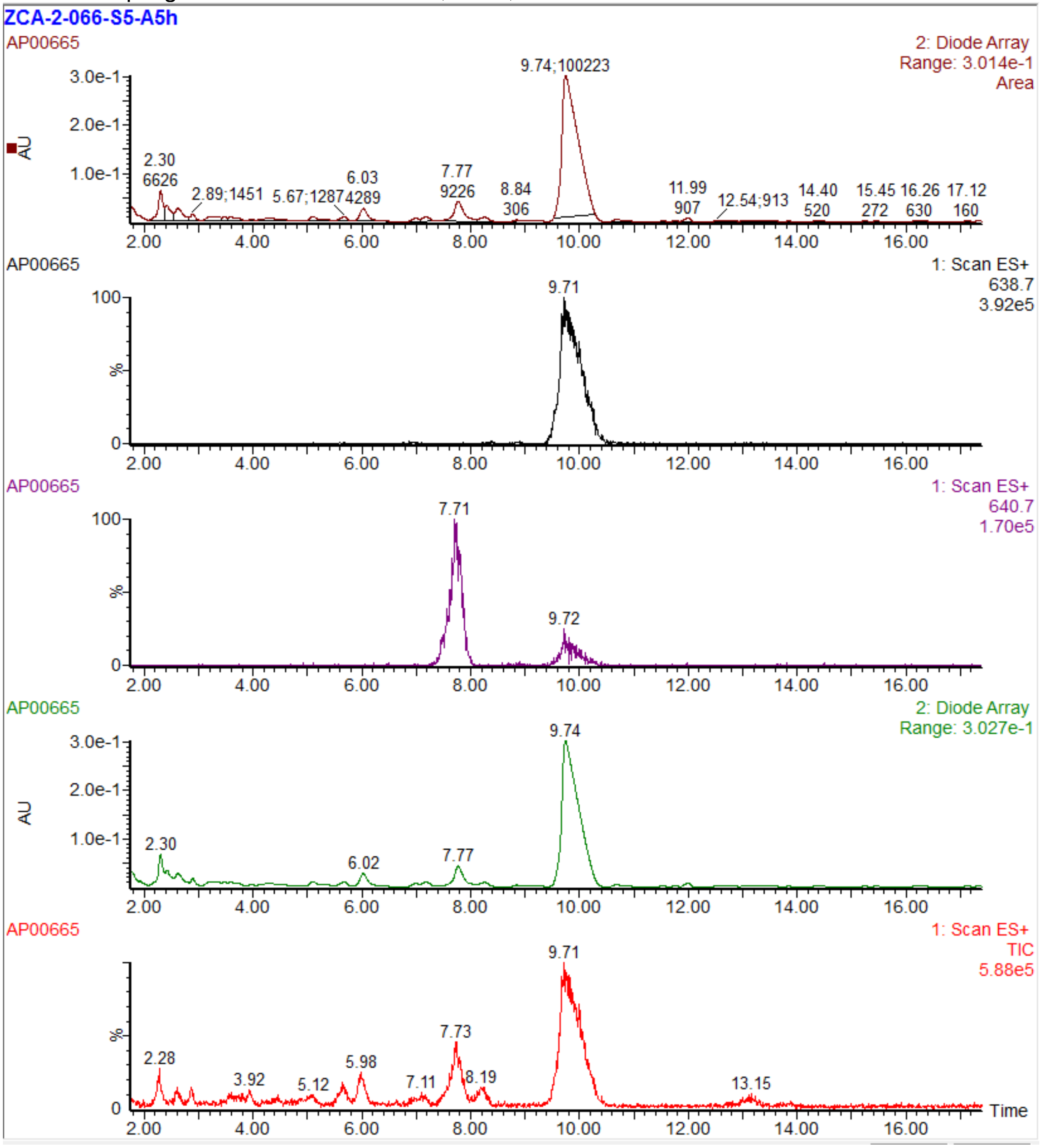

Glaser Coupling Conditions: Pra-Ala-Prs, NMP, 5 h

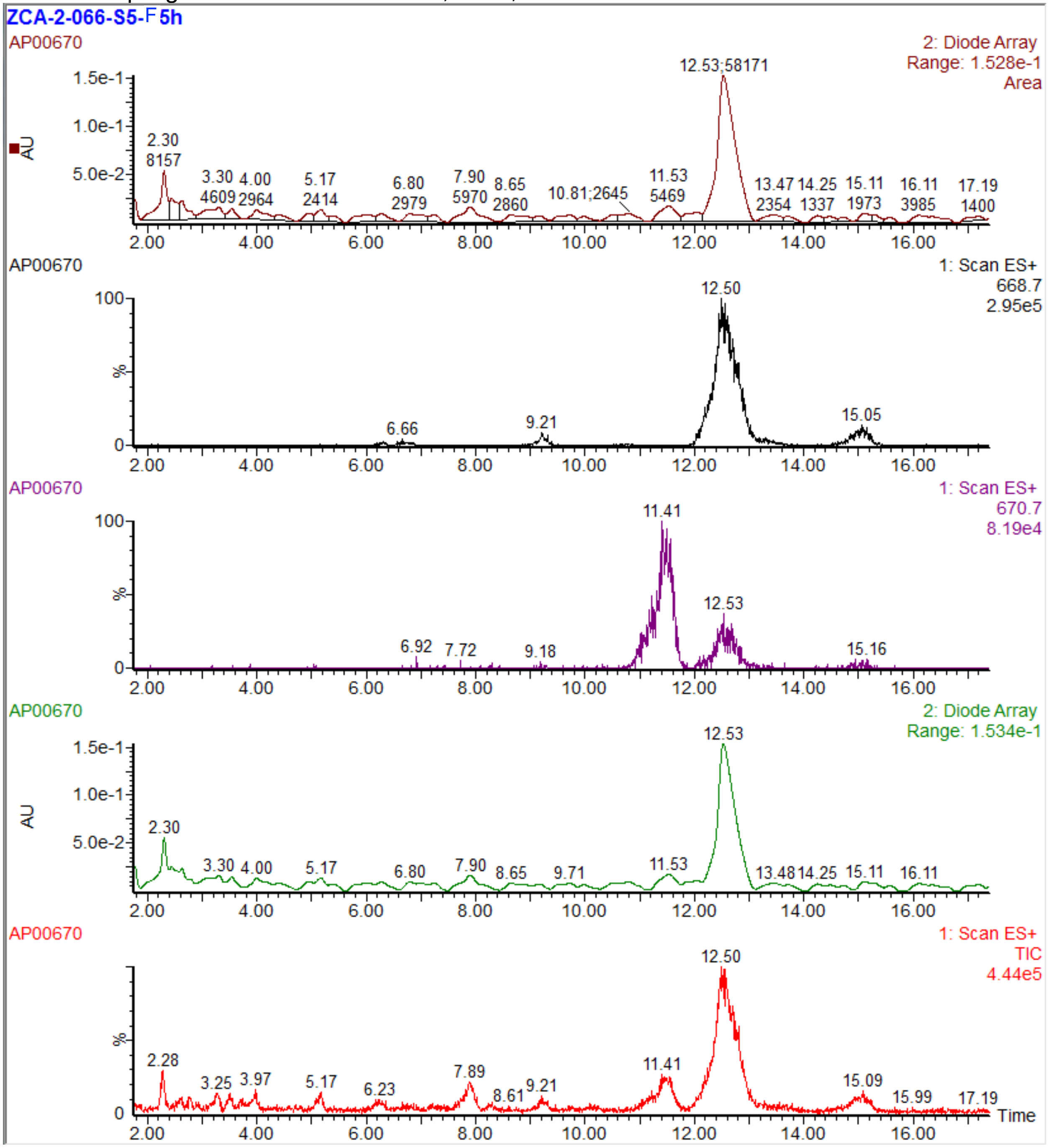

Glaser Coupling Conditions: Pra-Ala-Pra, 2-Me THF, 24 h

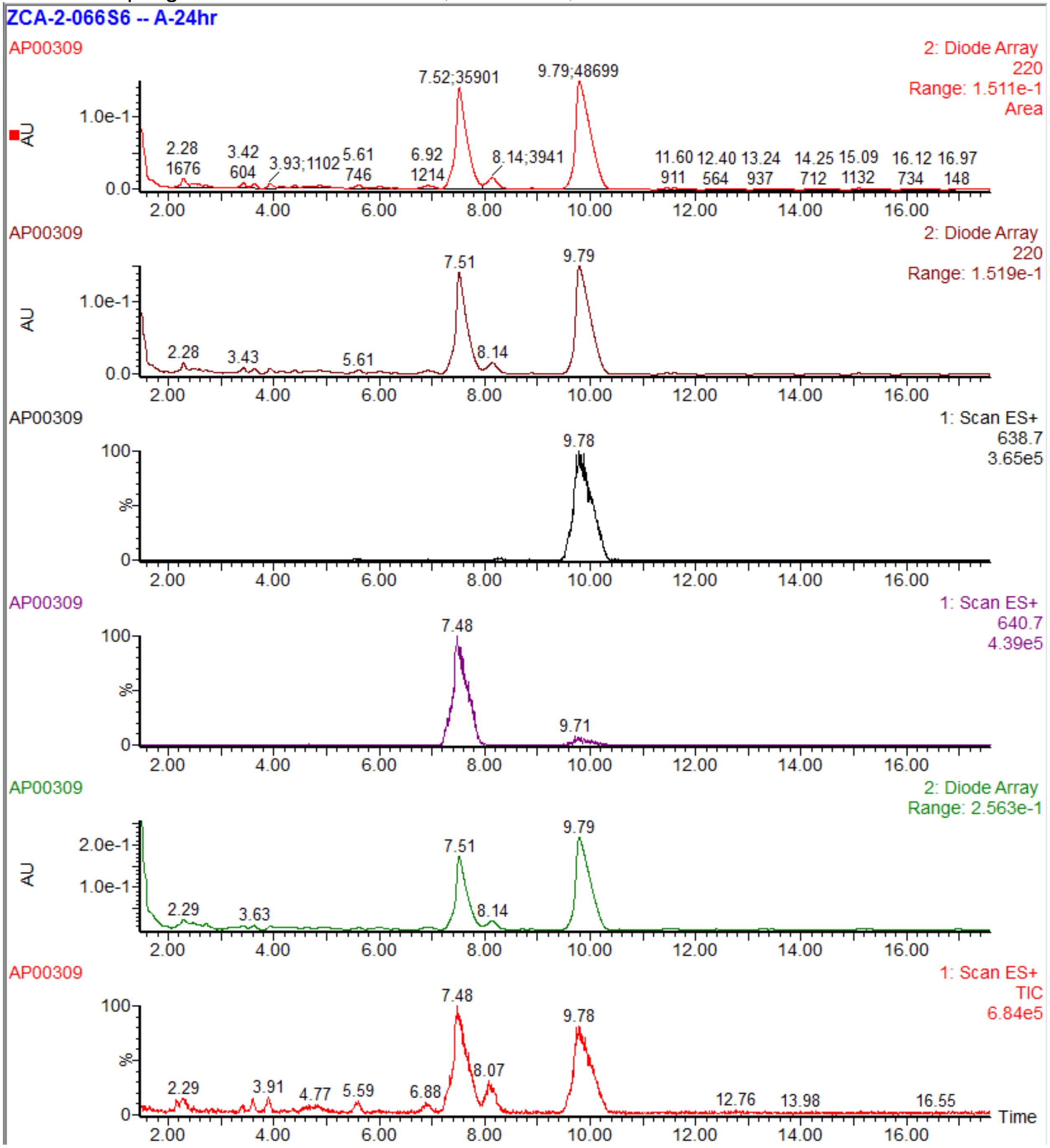

Glaser Coupling Conditions: Pra-Ala-Prs, 2-Me THF, 24 h

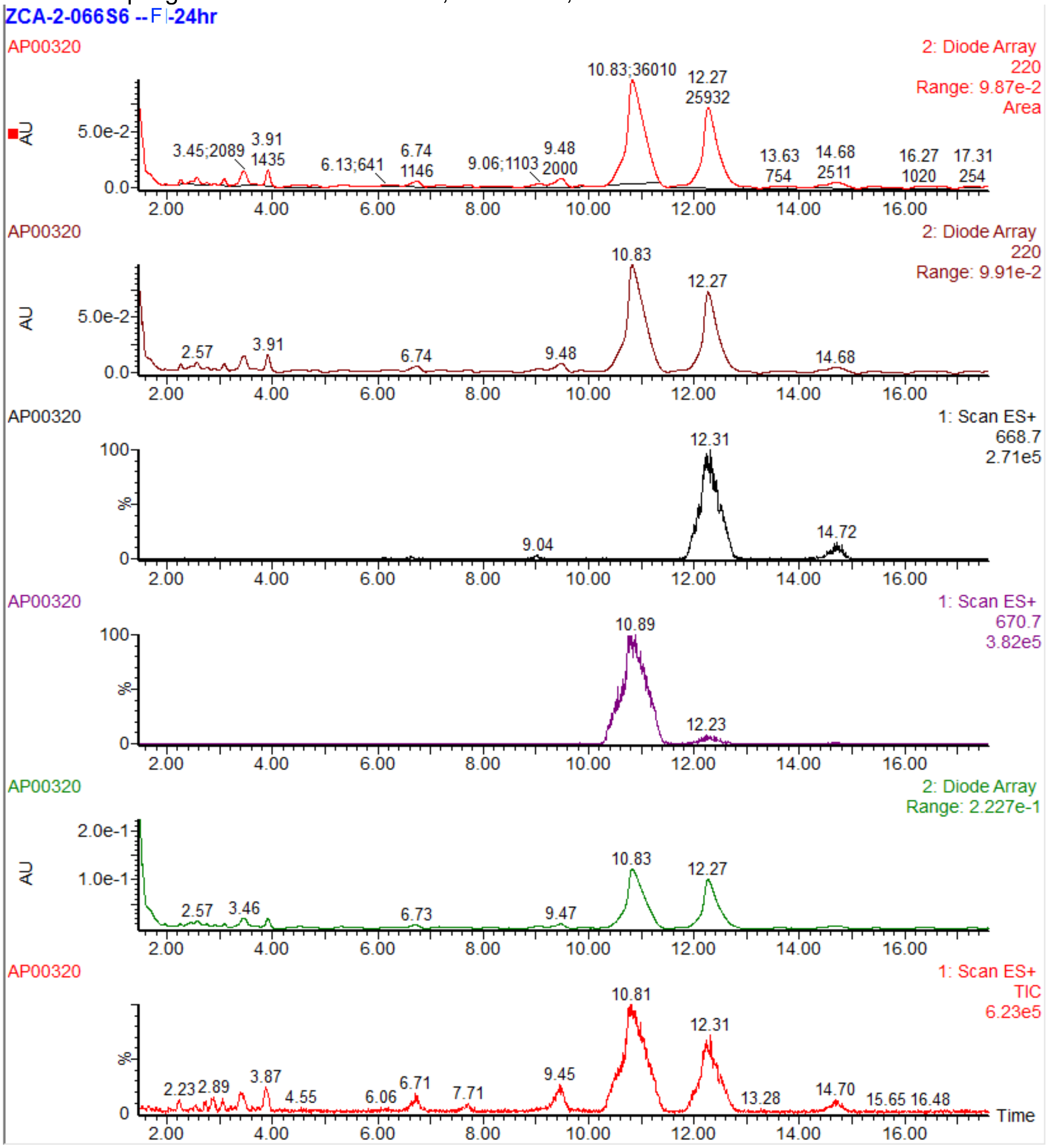

Glaser Coupling Conditions: Pra-Ala-Pra, THF, 24 h

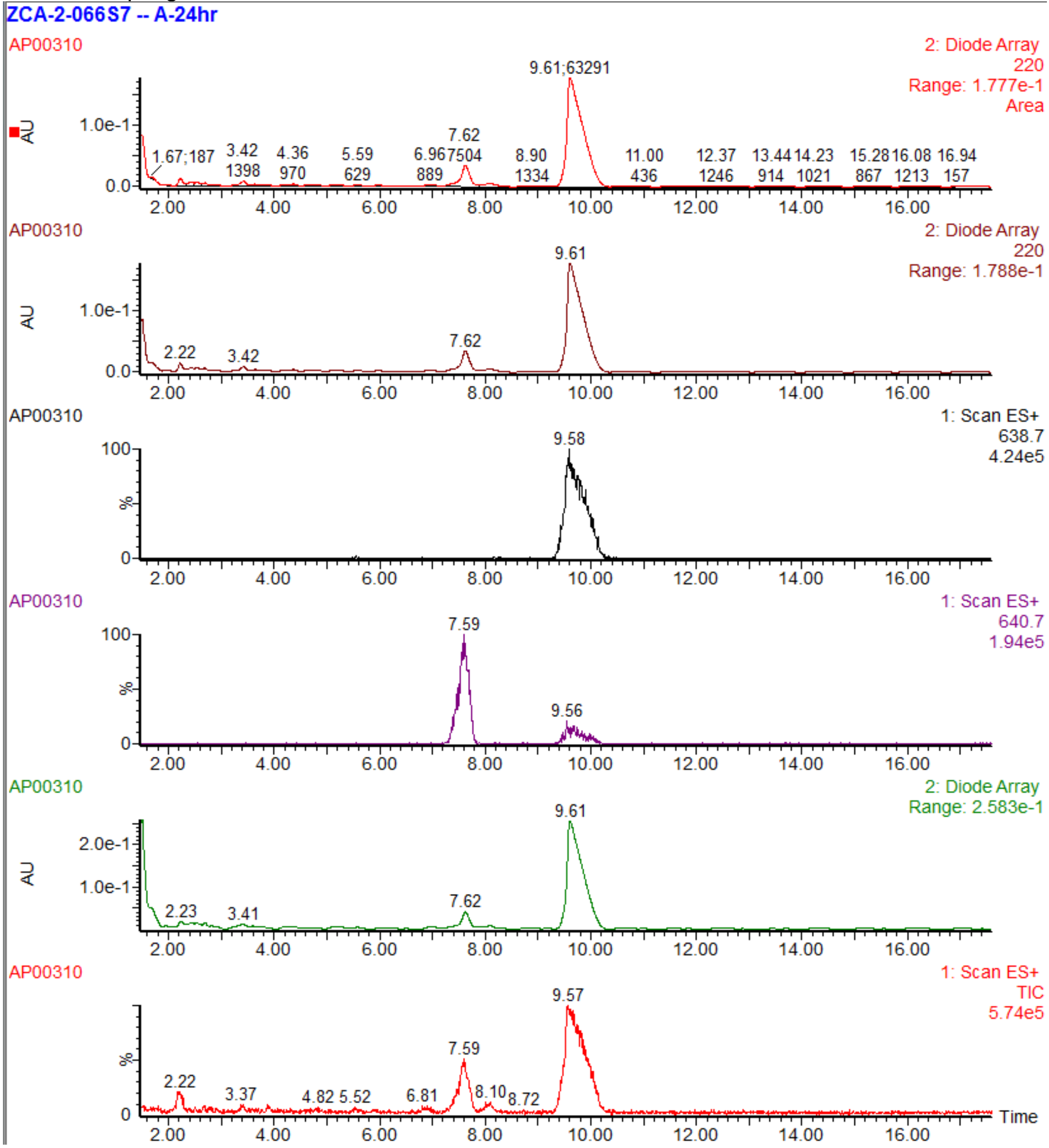

Glaser Coupling Conditions: Pra-Ala-Prs, THF, 24 h

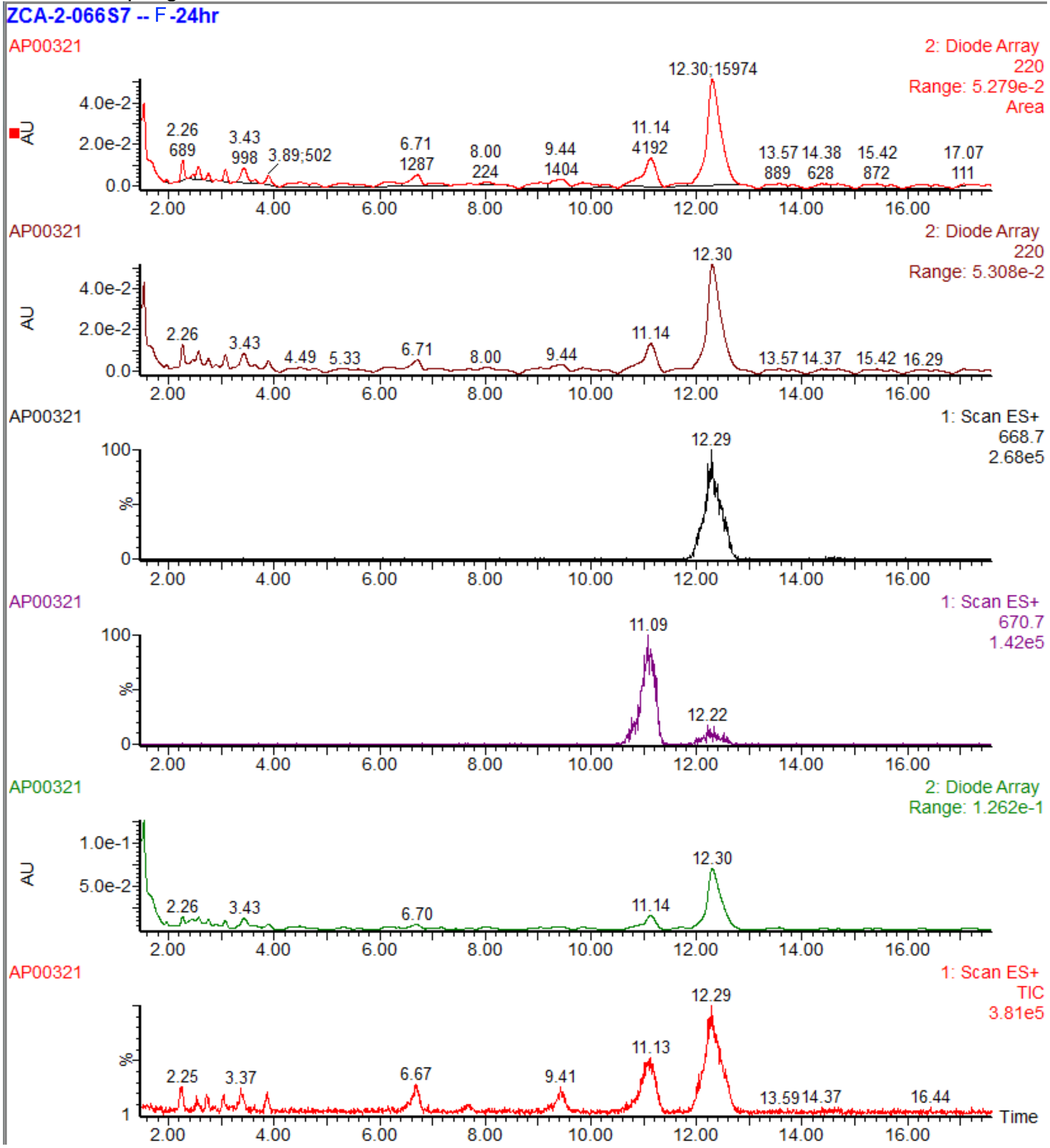

Glaser Coupling Conditions: Pra-Ala-Pra, ACN, 24 h

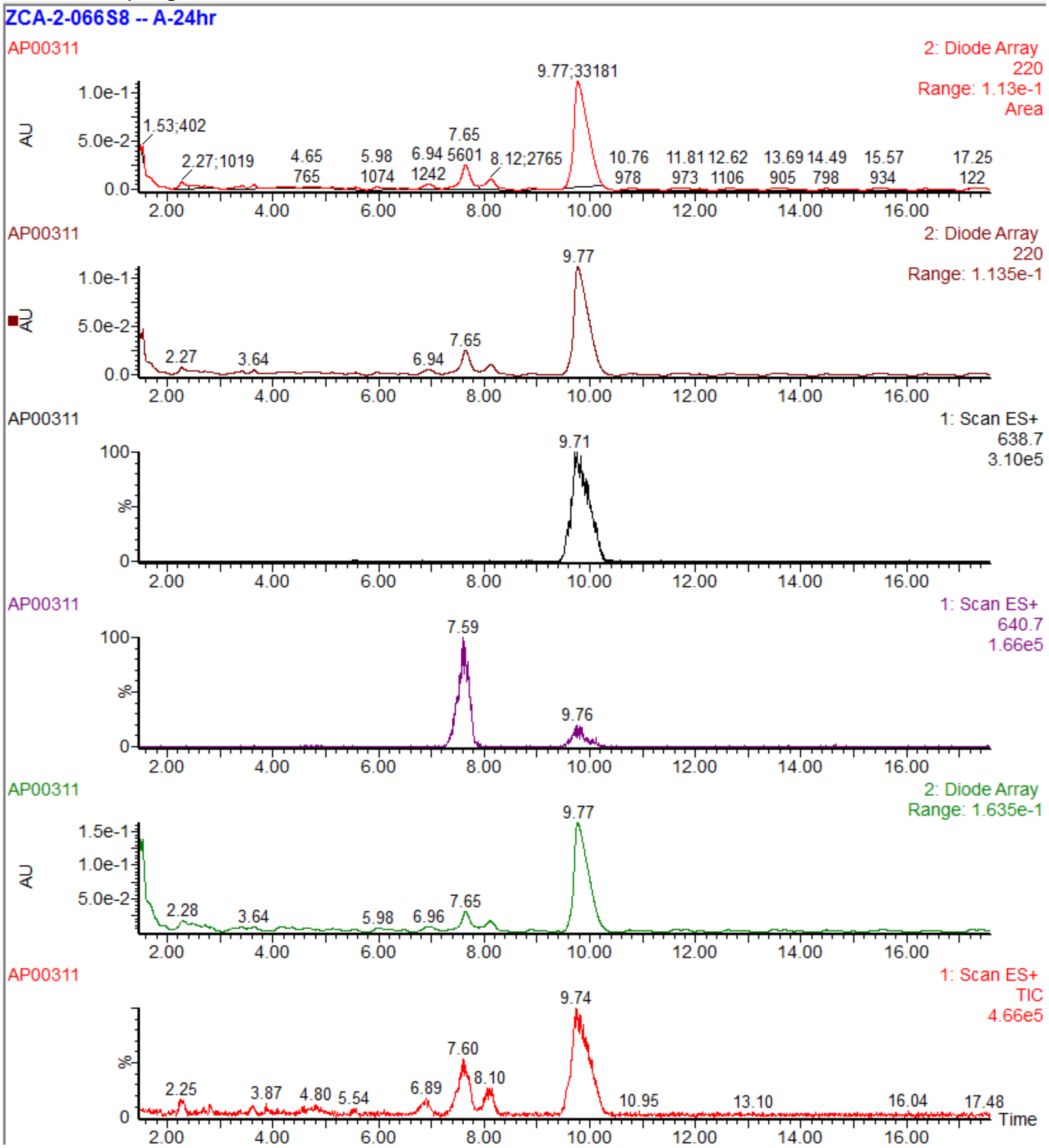

Glaser Coupling Conditions: Pra-Ala-Prs, ACN, 24 h

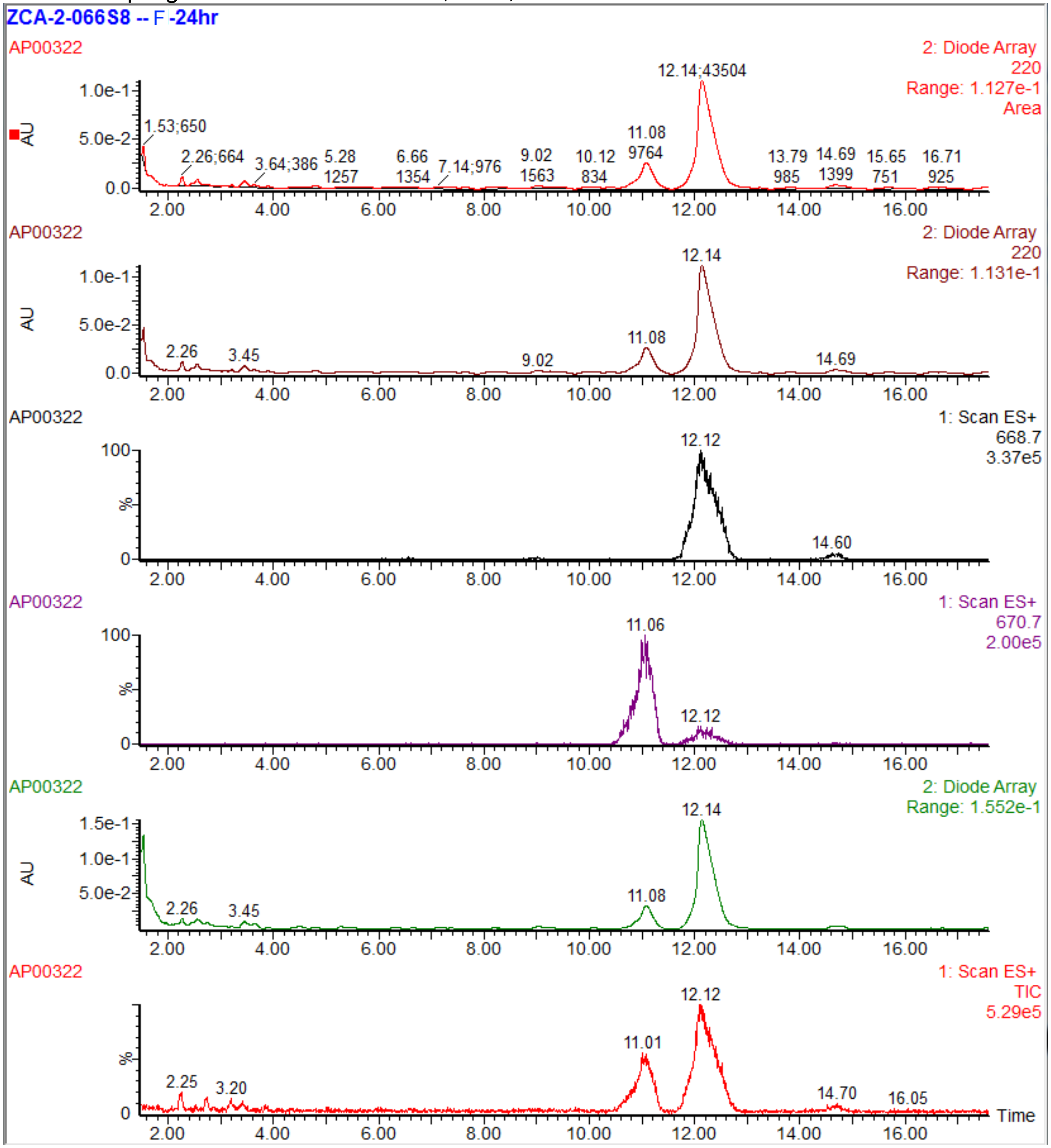

Glaser Coupling Conditions: Pra-Ala-Pra, MeOh, 24 h

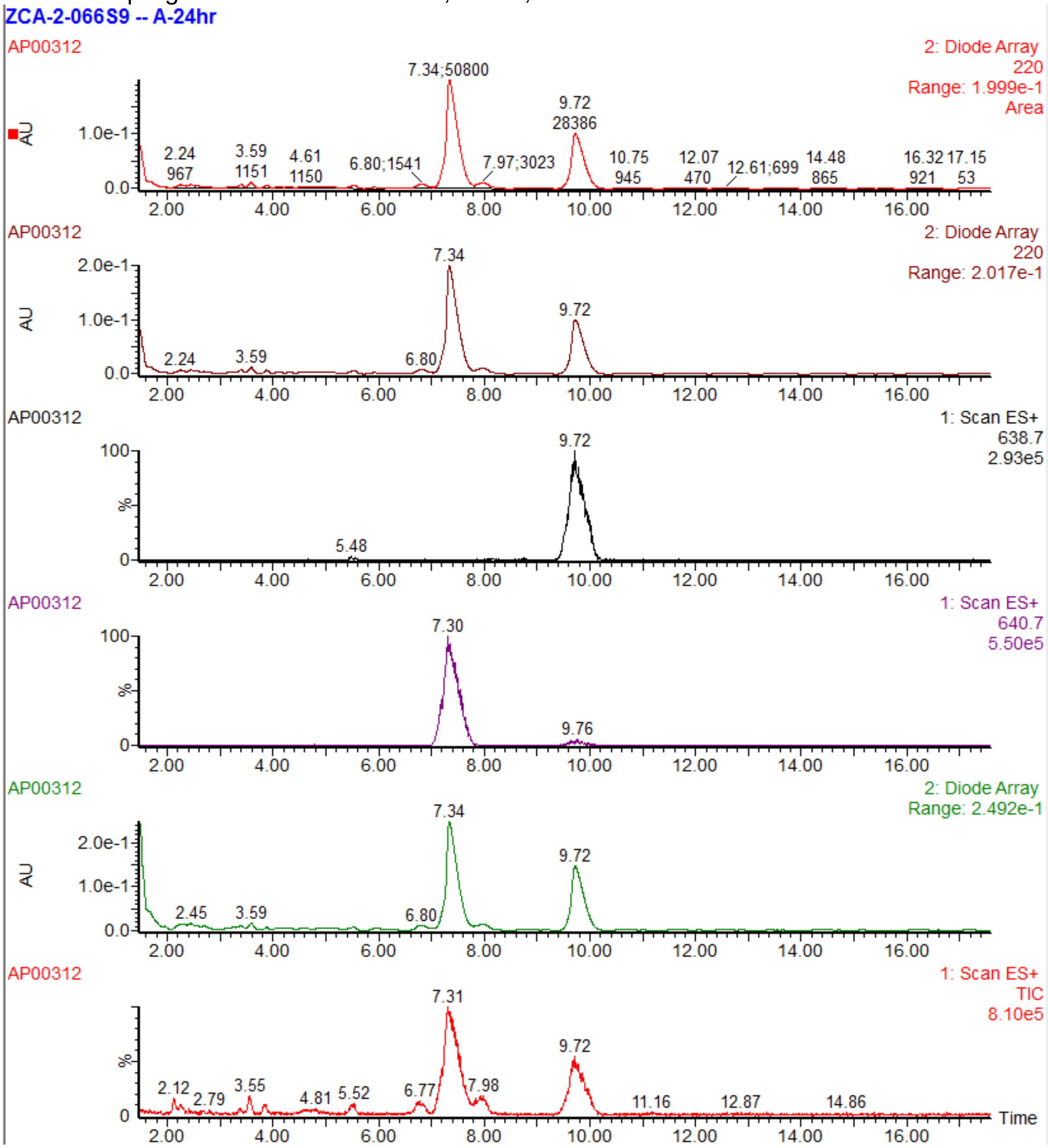

Glaser Coupling Conditions: Pra-Ala-Prs, MeOH, 24 h

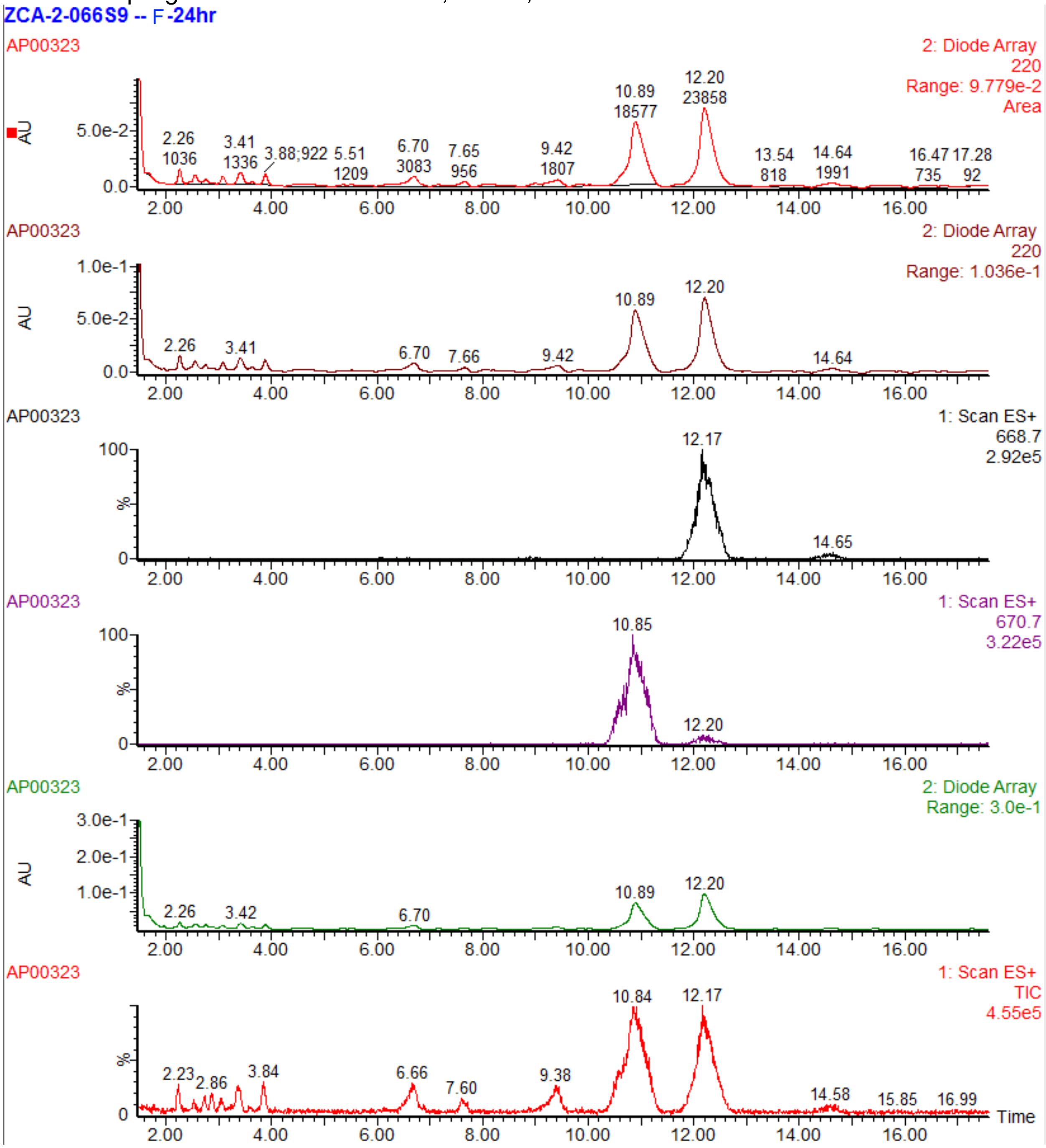

Glaser Coupling Conditions: Pra-Ala-Pra, DCM, 24 h

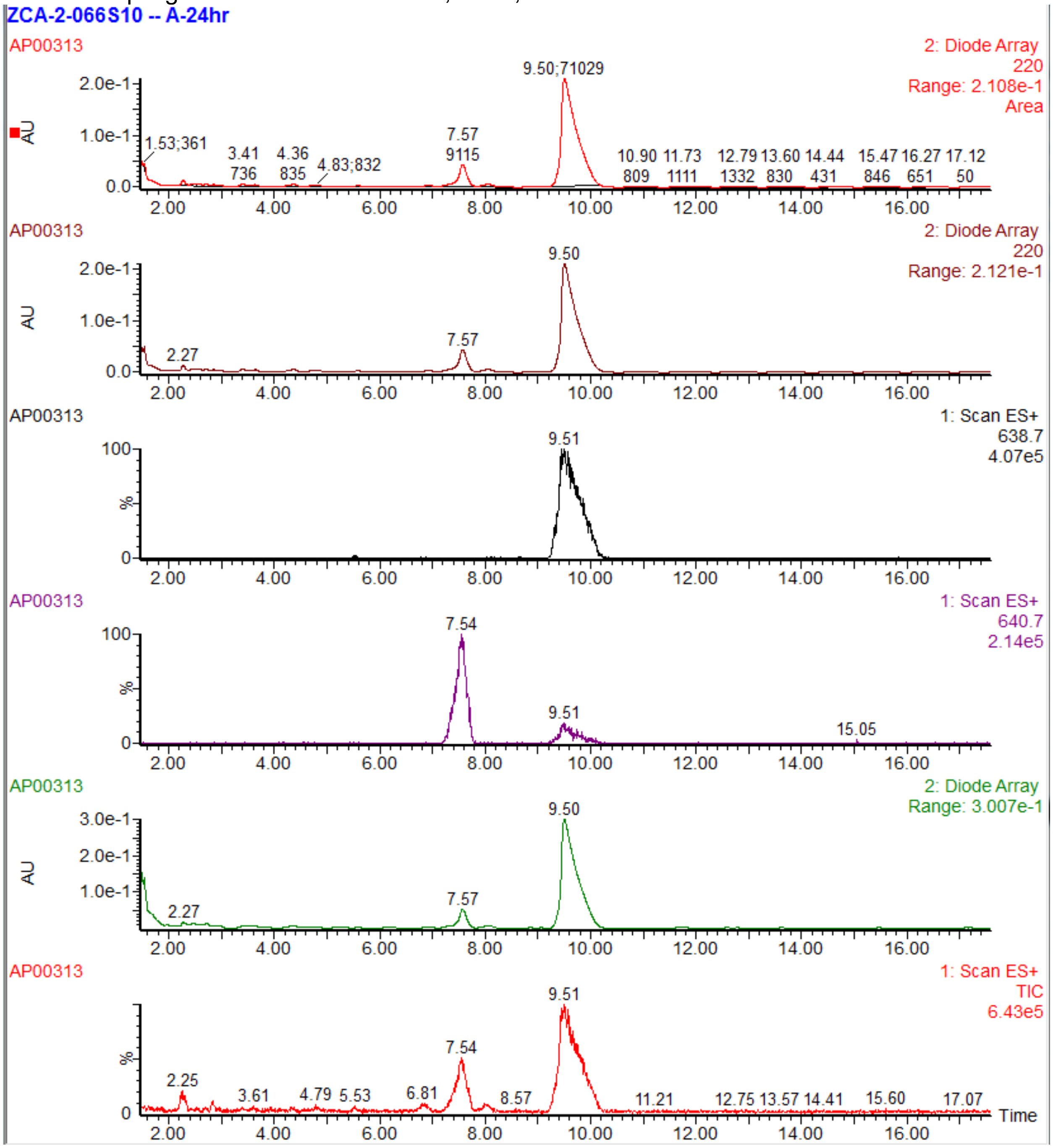

Glaser Coupling Conditions: Pra-Ala-Prs, DCM, 24 h

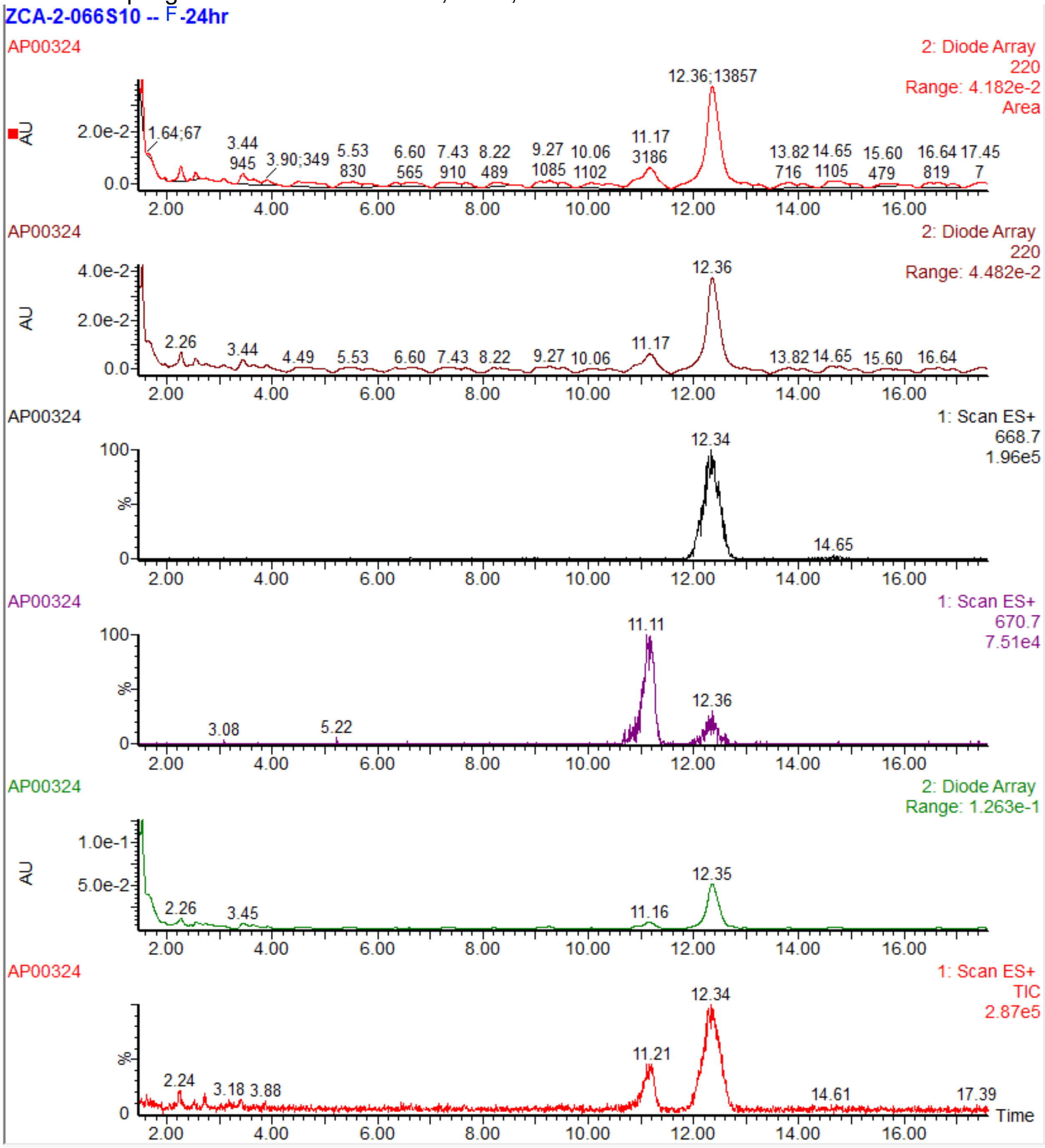

Glaser Coupling Conditions: Pra-Ala-Pra, DMA, 5 h, 4 C

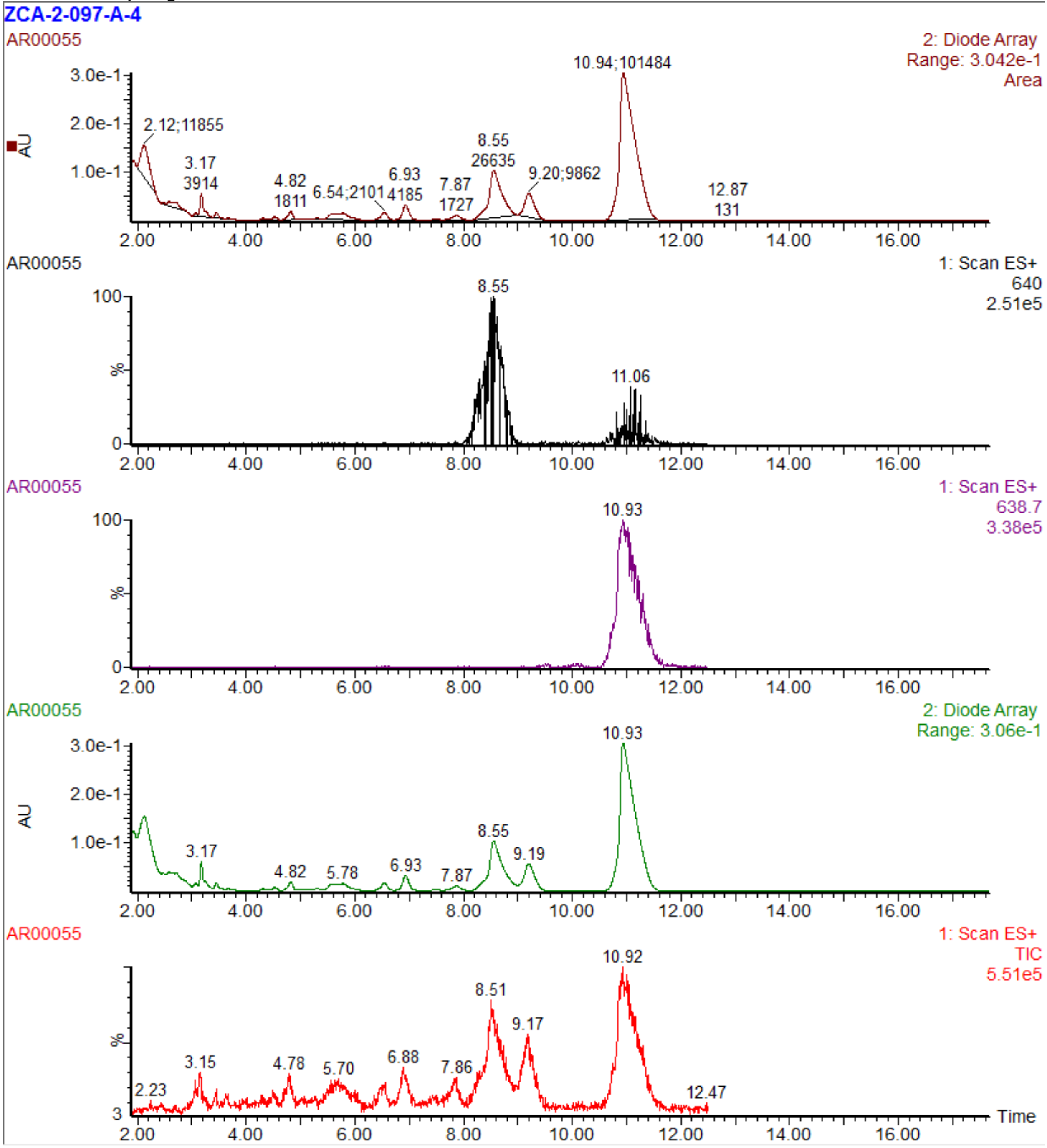

Glaser Coupling Conditions: Pra-Ala-Pra, DMA, 5 h, 25 C

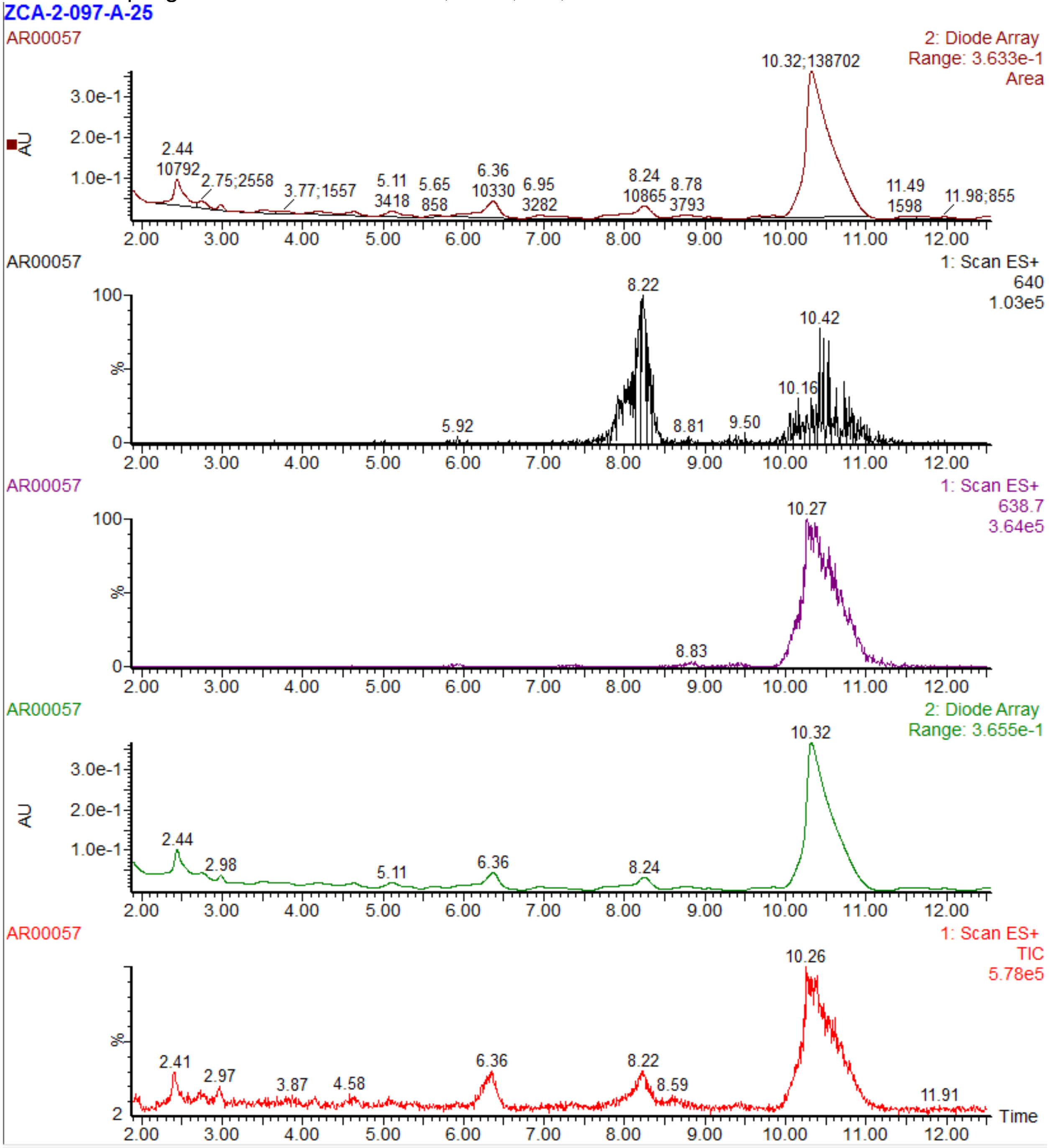

Glaser Coupling Conditions: Pra-Ala-Pra, DMA, 5 h, 37 C

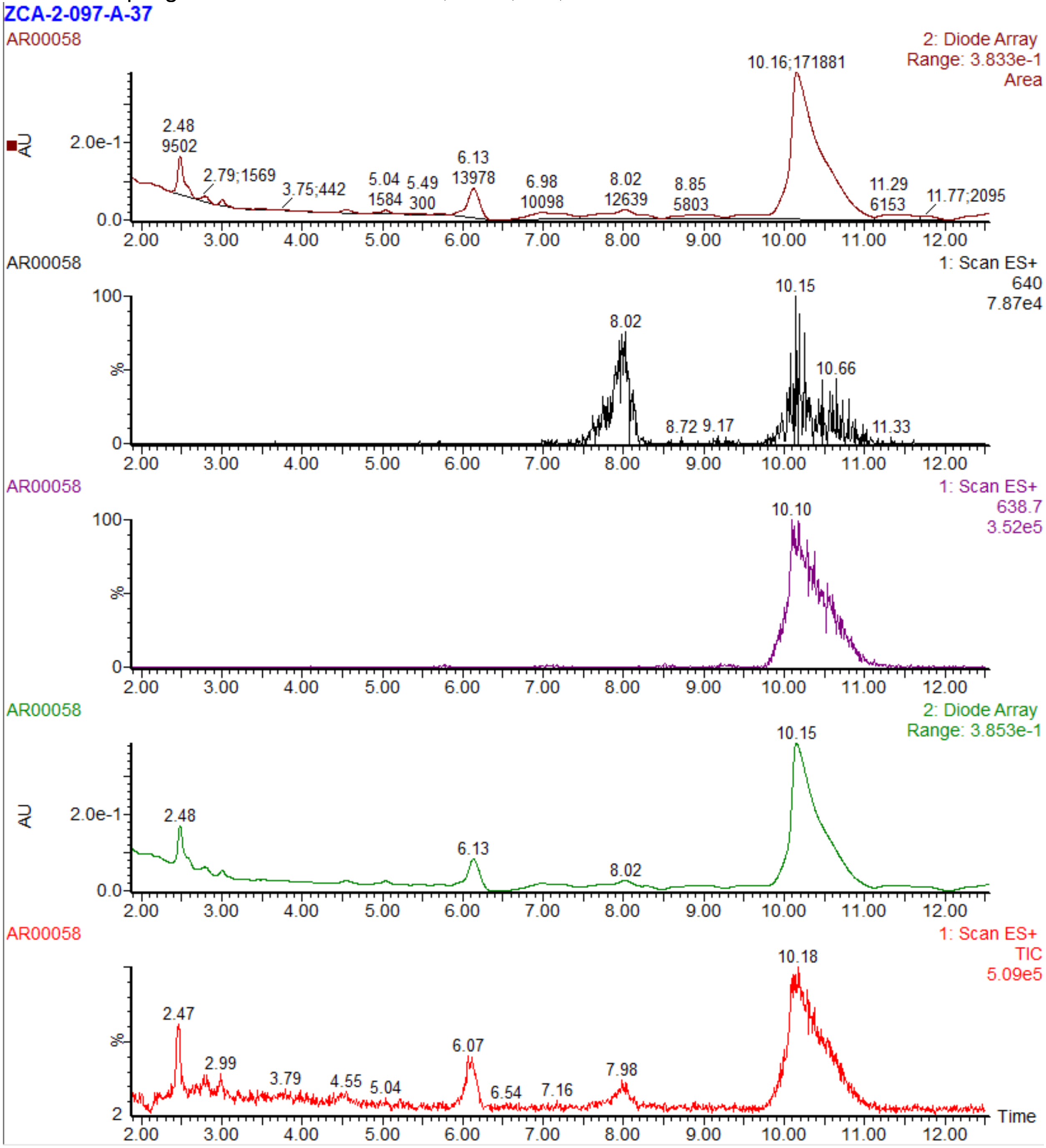

Glaser Coupling Conditions: Pra-Ala-Prs, DMA, 5 h, 4 C

ZCA-2-097-F -4

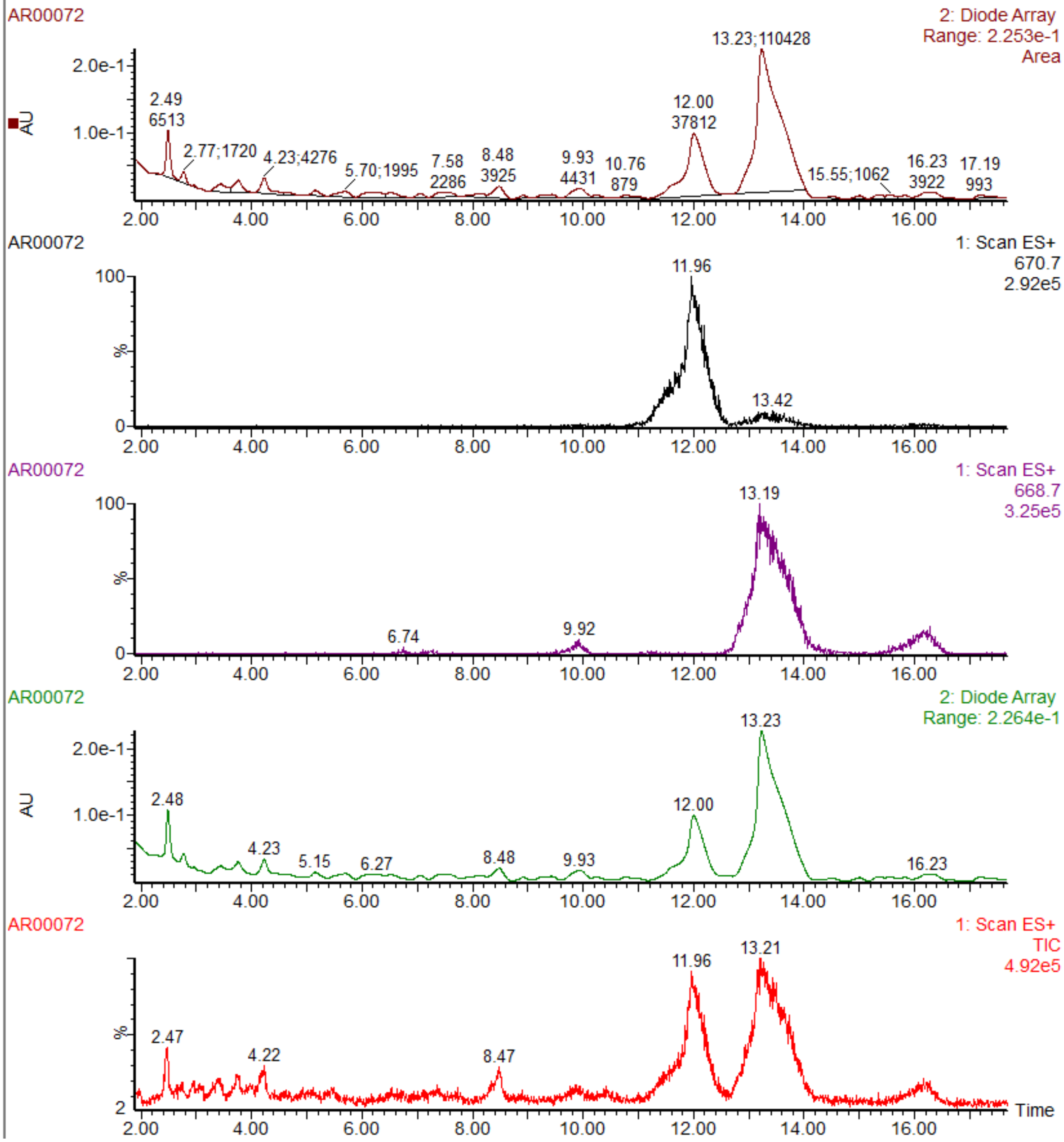

Glaser Coupling Conditions: Pra-Ala-Prs, DMA, 5 h, 25 C

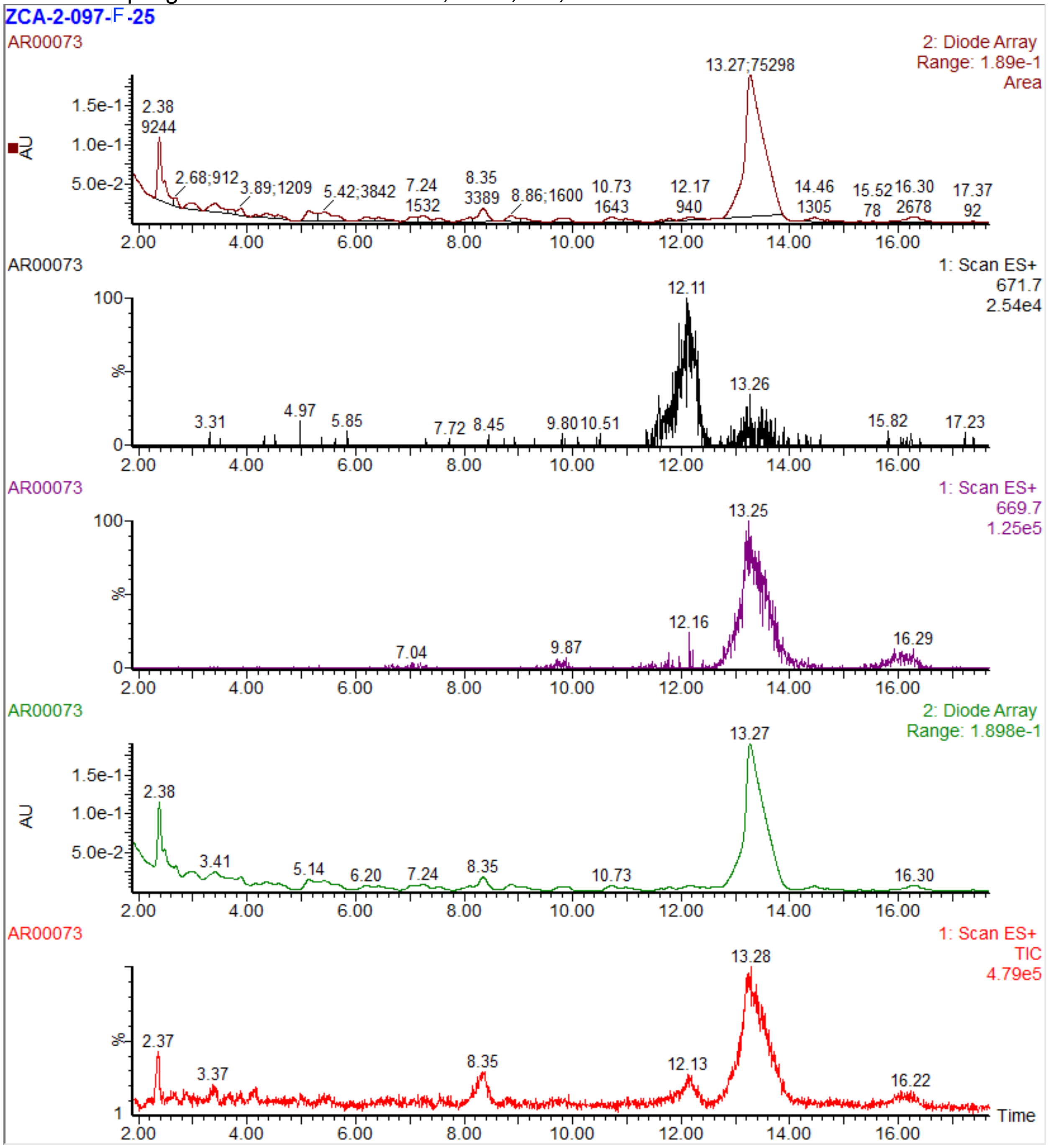

Glaser Coupling Conditions: Pra-Ala-Prs, DMA, 5 h, 37 C

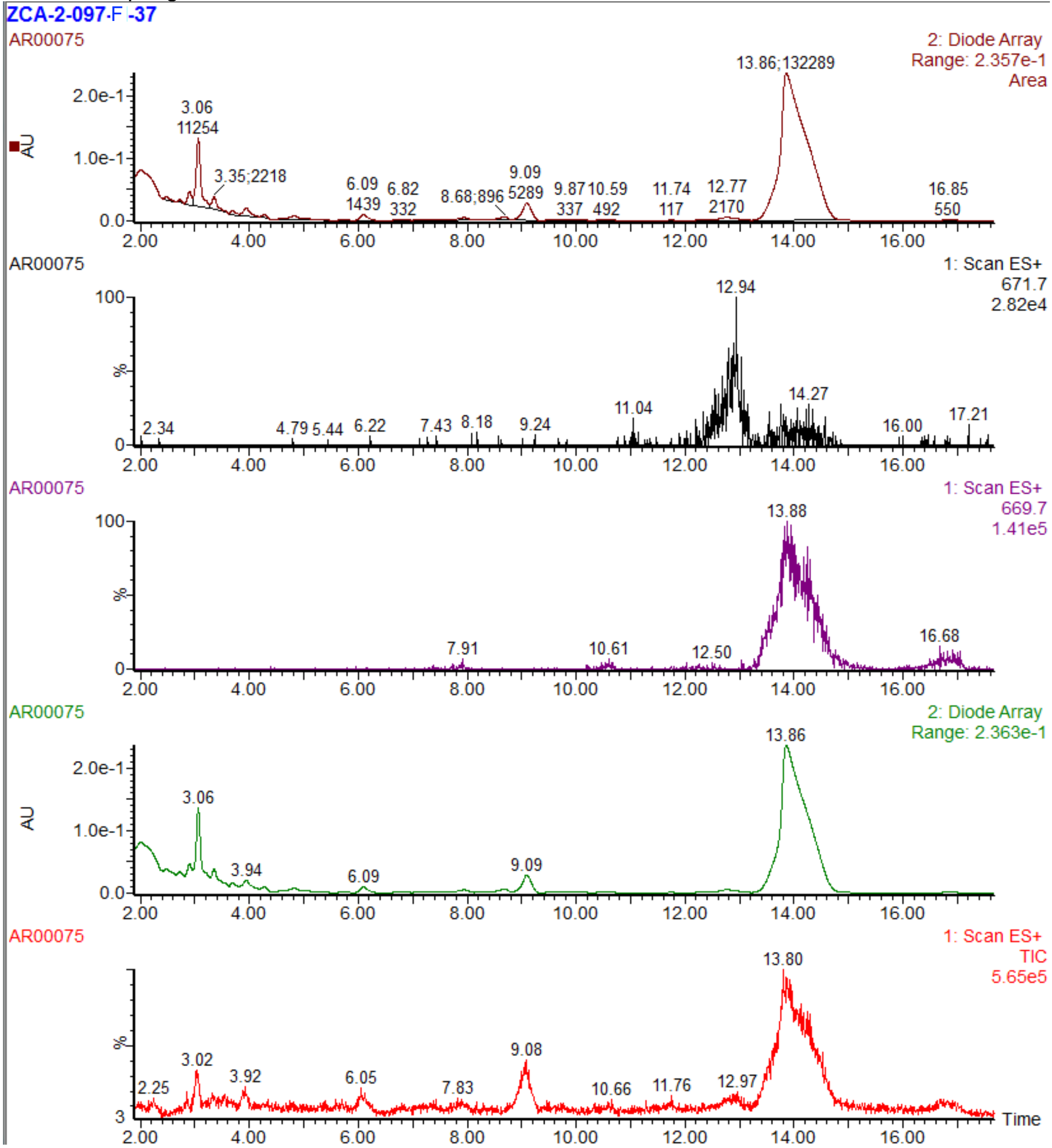

Glaser Coupling Conditions: Pra-Ala-Pra, DMA, 5 h, 37 C, under Ar

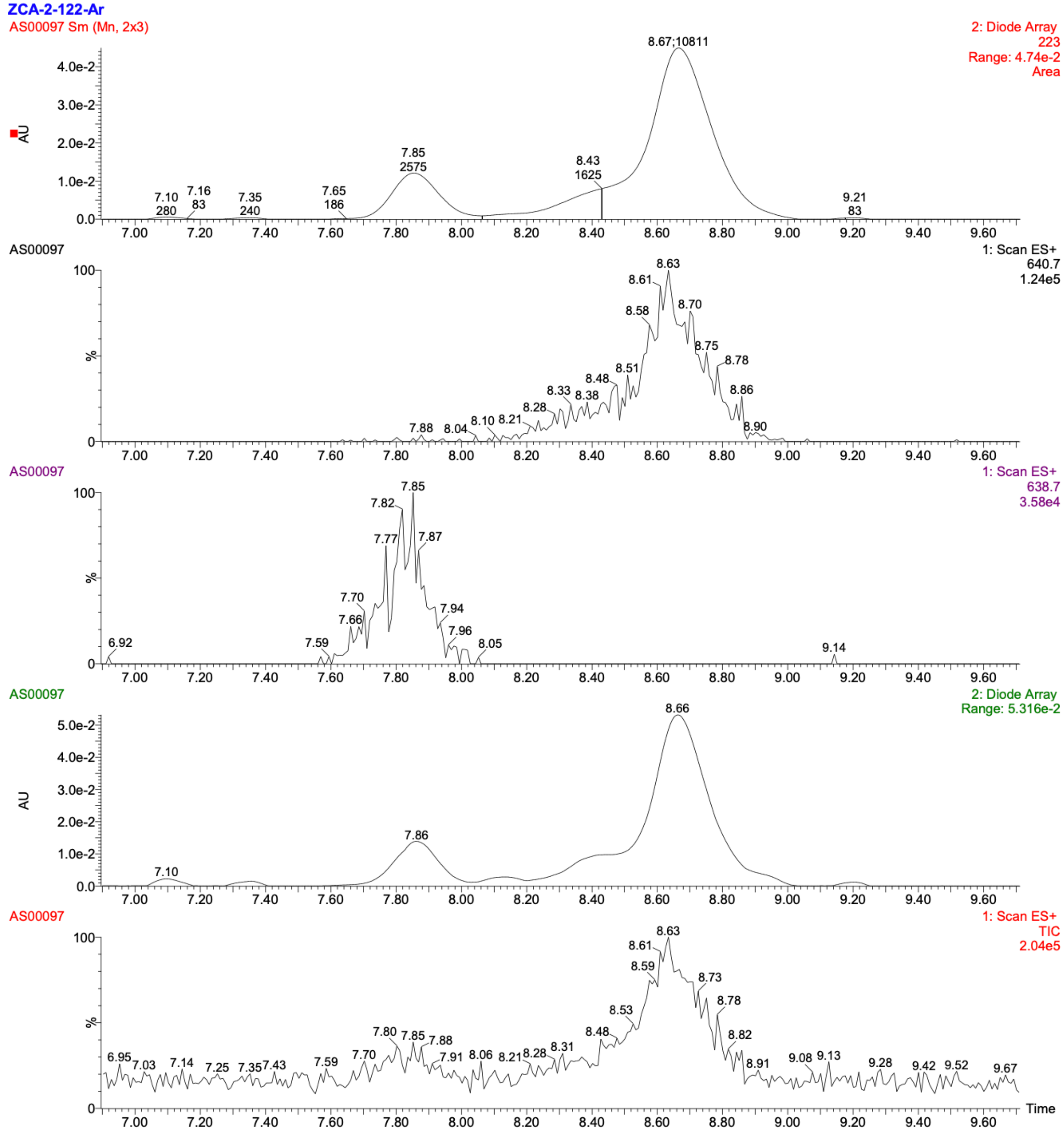

Glaser Coupling Conditions: Pra-Ala-Pra, DMA, 5 h, 37 C, vertical tube

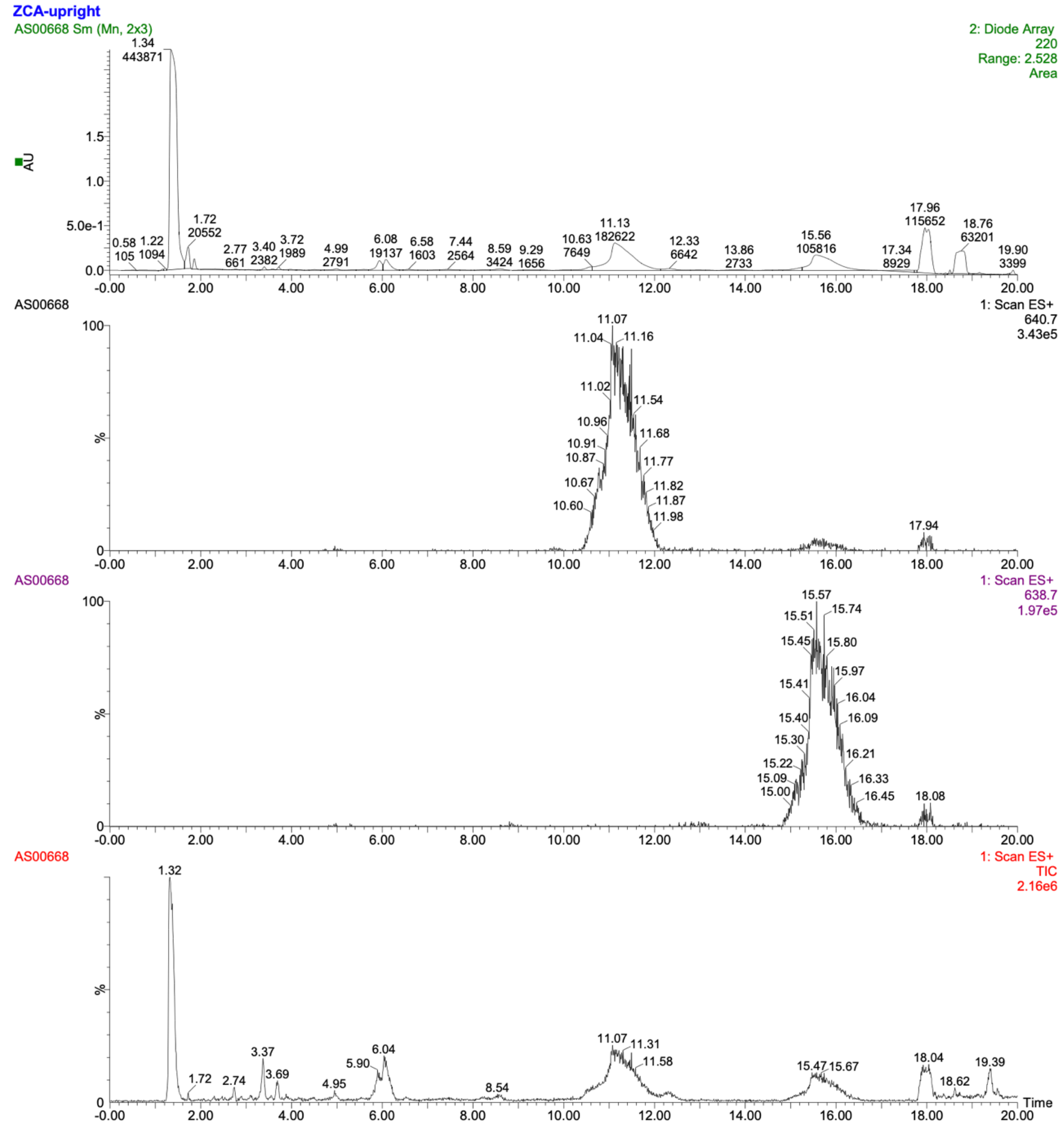

Diyne-Braced Model Peptides

Compound 3: Model Peptide A

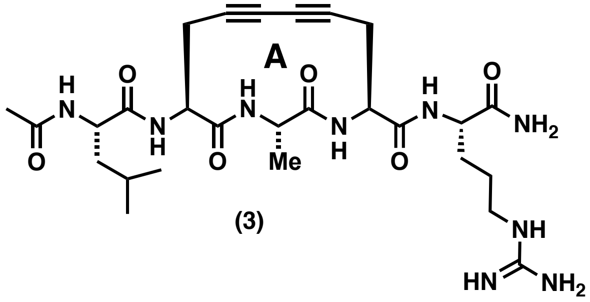

(3)

Chemical Formula: $C_{27}H_{41}N_9O_6$

Molecular Weight: 587.68

The product was purified by mass-directed prep LC. The crude material was purified on a Waters Autopurification LC with a Waters BEH C18 column (5 um, 19x160 mm) using a 0.1% aqueous trifluoroacetic acid:acetonitrile gradient (30 mL/min, main segment of gradient at 15-35% acetonitrile over 8 minutes) at ambient temperature. Fractionation was triggered by a Waters QDa single quadrupole mass spec (ESI+).
Retention time: 4.3 min

HRMS:

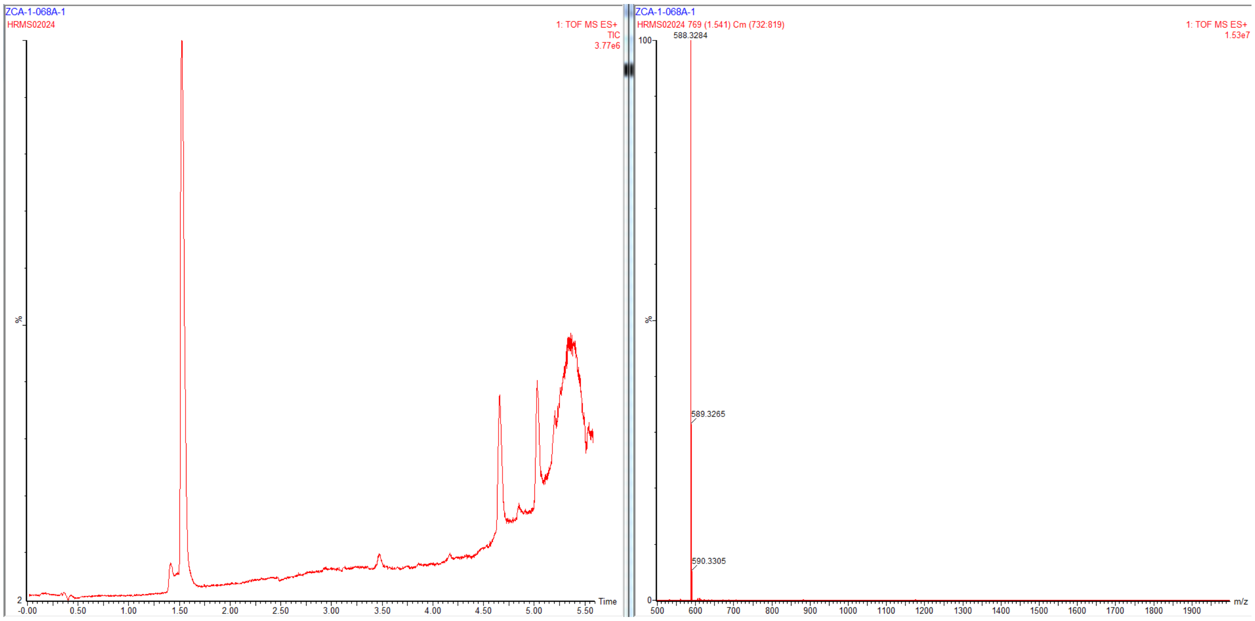

[1]H Spectrum

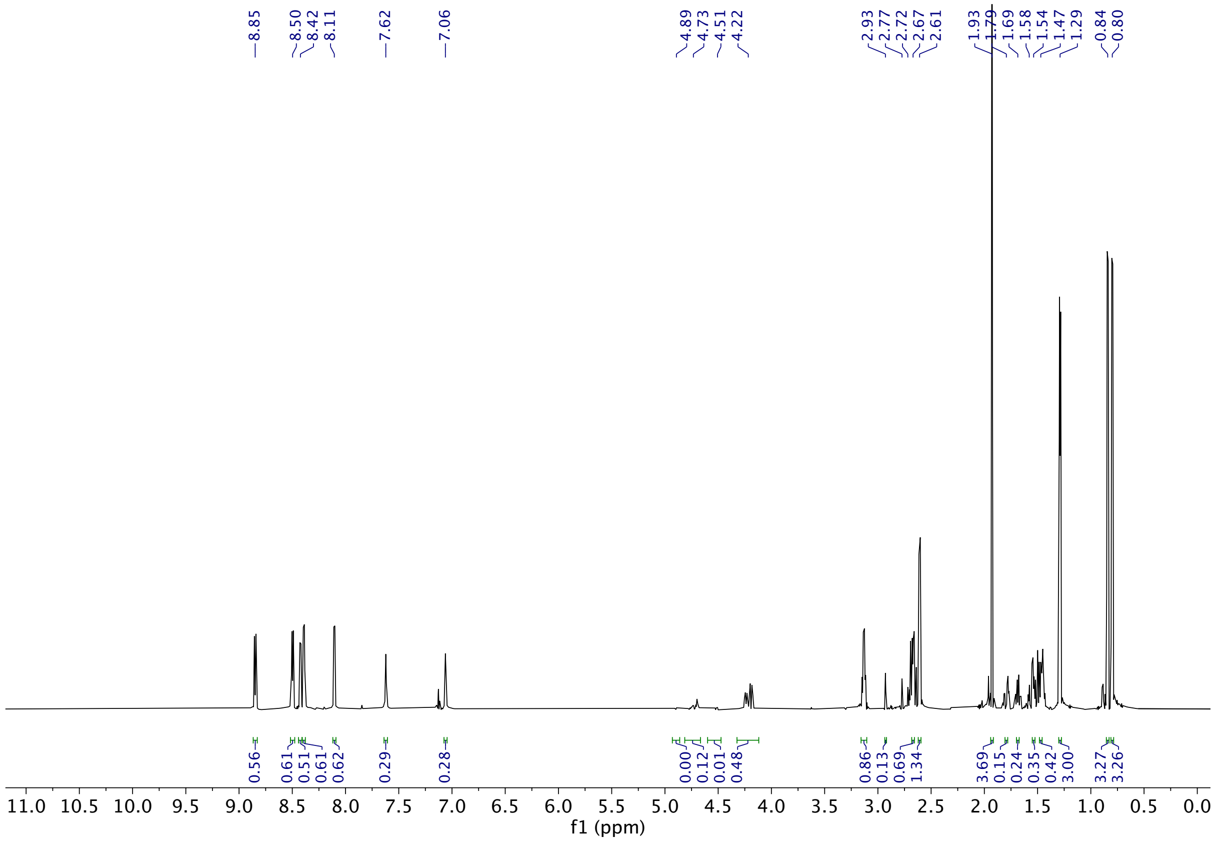

Assignment Table

Residue	Amide NH	Hα	Hβ	Hγ	other
**Leu1**	8.11 (d, *J* = 7.1 Hz)	4.19	1.47	1.58	δ: 0.84, 0.80, Ac: 1.93
**Pra2**	8.39 (d, *J* = 7.0 Hz)	4.50	2.61		
**Ala3**	8.50 (d, *J* = 9.2 Hz)	4.58	1.29		
**Pra4**	8.85 (d, *J* = 10.1 Hz)	4.87	2.66		
**Arg5**	8.42 (d, *J* = 7.6 Hz)	4.24	1.79, 1.69	1.54	δ: 3.12, ε: 7: 12, NH: 7.62, 7.06

Compound 4: Model Peptide B

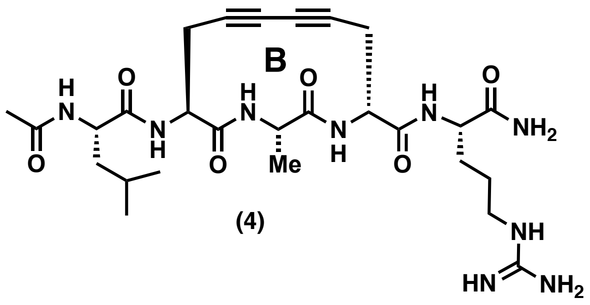

(4)

Chemical Formula: $C_{27}H_{41}N_9O_6$

Molecular Weight: 587.68

The product was purified by mass-directed prep LC. The crude material was purified on a Waters Autopurification LC with a Waters BEH C18 column (5 um, 19x160 mm) using under isocratic conditions (13% B - acetonitrile / 87% A - 0.1% aqueous trifluoroacetic acid; 30 mL/min for 15 minutes) at ambient temperature. Fractionation was triggered by a Waters QDa single quadrupole mass spec (ESI+). Retention time: 8.6 min

HRMS:

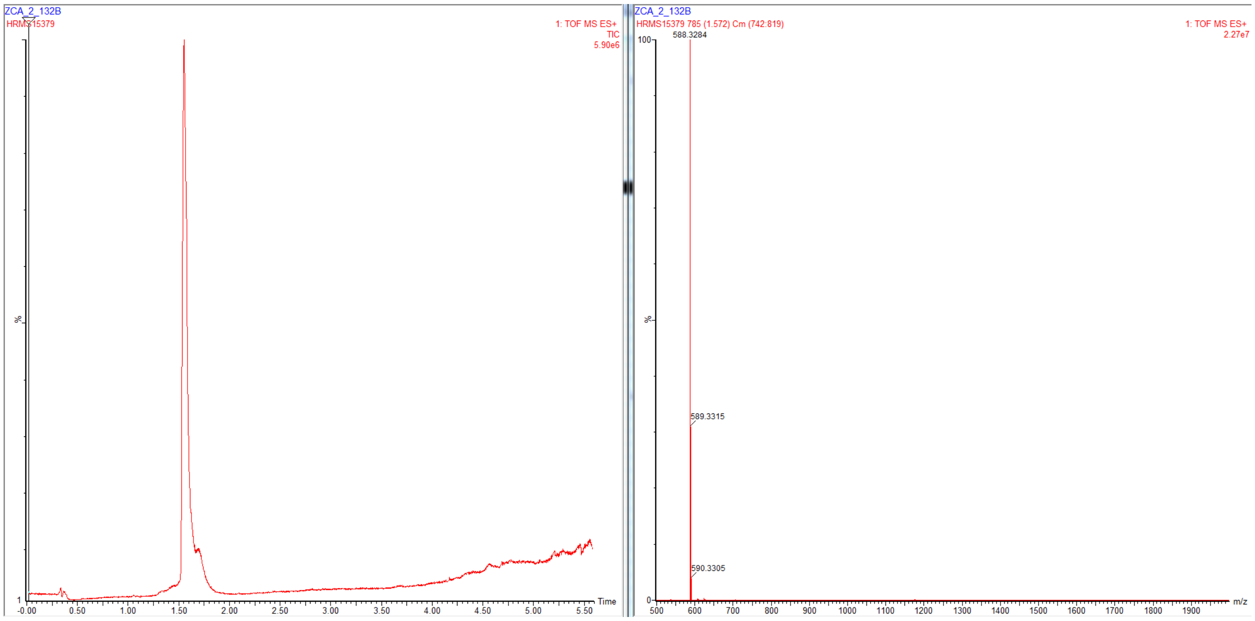

^{1}H Spectrum

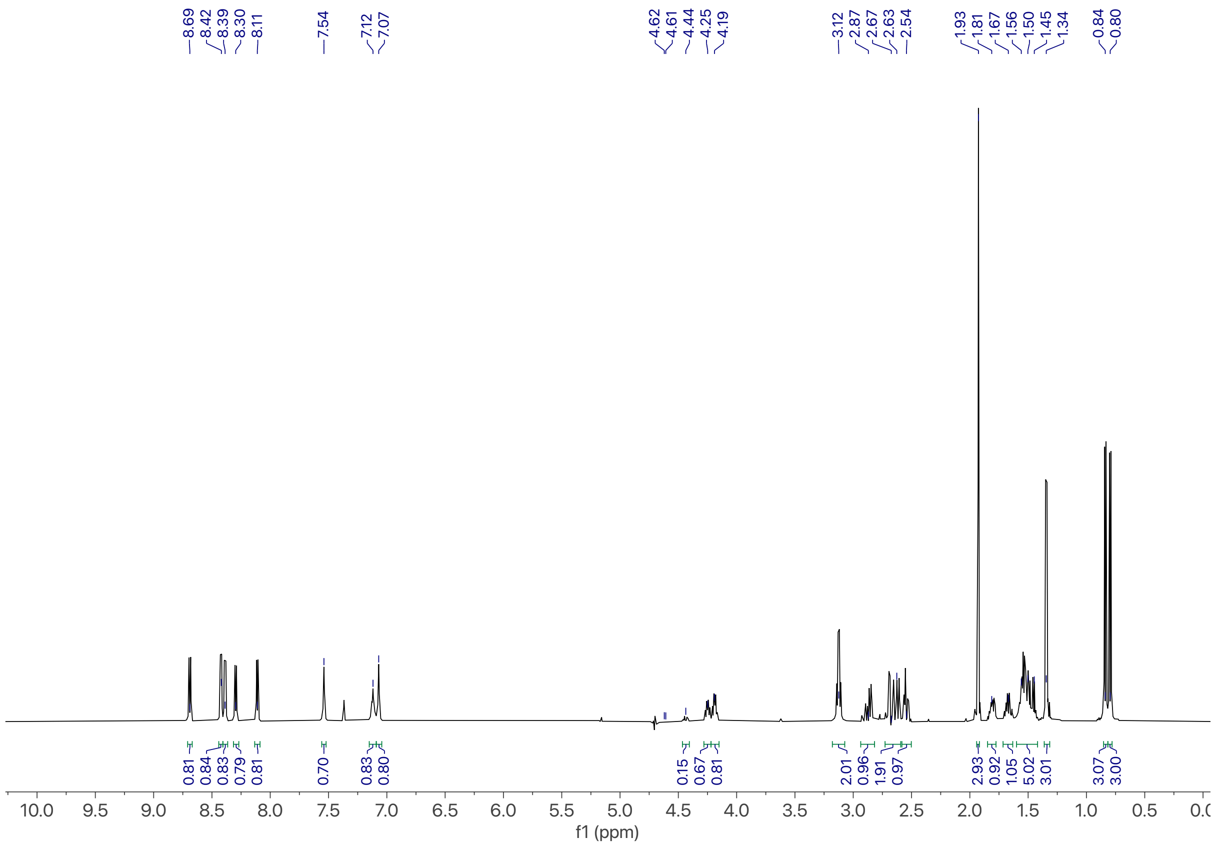

Assignment Table

Residue	Amide NH	Hα	Hβ	Hγ	other
**Leu1**	8.11 (d, *J* = 6.9 Hz)	4.19	1.50	1.45	δ: 0.84, 0.80, Ac: 1.93
**Pra2**	8.42 (d, *J* = 6.7 Hz)	4.44	2.63, 2.54		
**Ala3**	8.69 (d, *J* = 8.9 Hz)	4.61	1.34		
**D-Pra4**	8.39 (d, *J* = 8.0 Hz)	4.62	2.87, 2.67		
**Arg5**	8.30 (d, *J* = 7.4 Hz)	4.25	1.81, 1.67	1.56	δ: 3.12, ε: 7: 12, NH: 7.54, 7.07

Compound 5: Model Peptide C

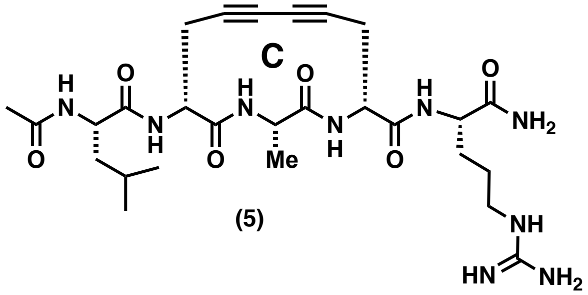

(5)

Chemical Formula: $C_{27}H_{41}N_9O_6$

Molecular Weight: 587.68

The product was purified by mass-directed prep LC. The crude material was purified on a Waters Autopurification LC with a Waters BEH C18 column (5 um, 19x160 mm) using under isocratic conditions (20% B - acetonitrile / 80% A - 0.1% aqueous trifluoroacetic acid; 30 mL/min for 8 minutes) at ambient temperature. Fractionation was triggered by a Waters QDa single quadrupole mass spec (ESI+).
Retention time: 4.3 min

HRMS:

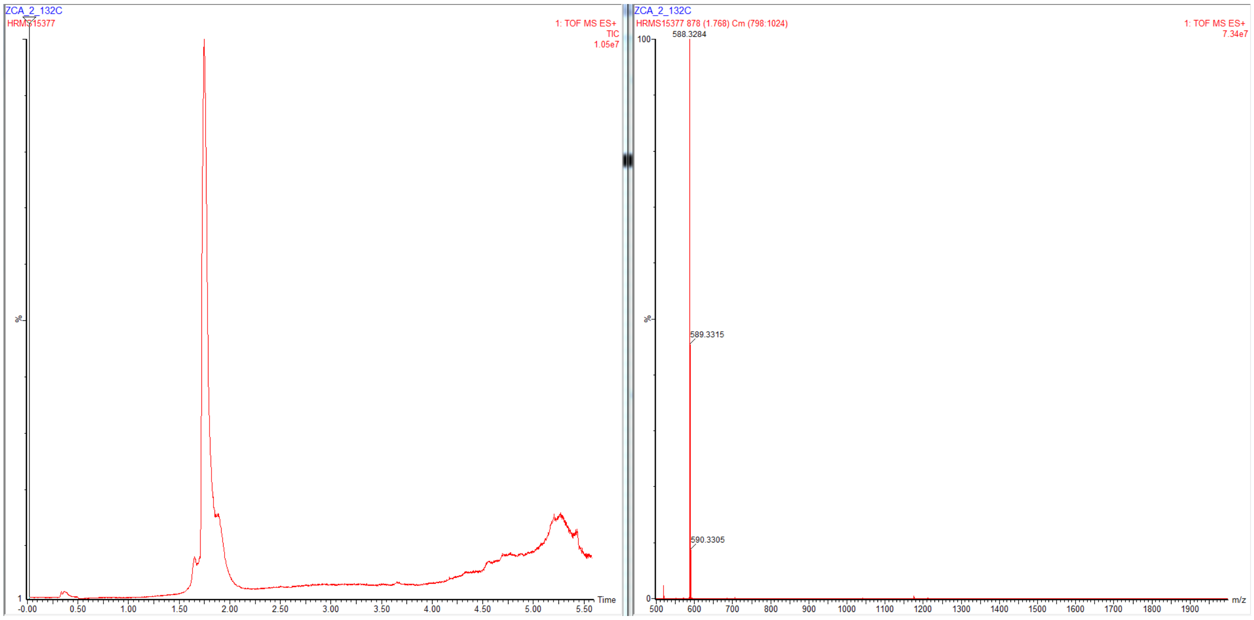

[1]H Spectrum

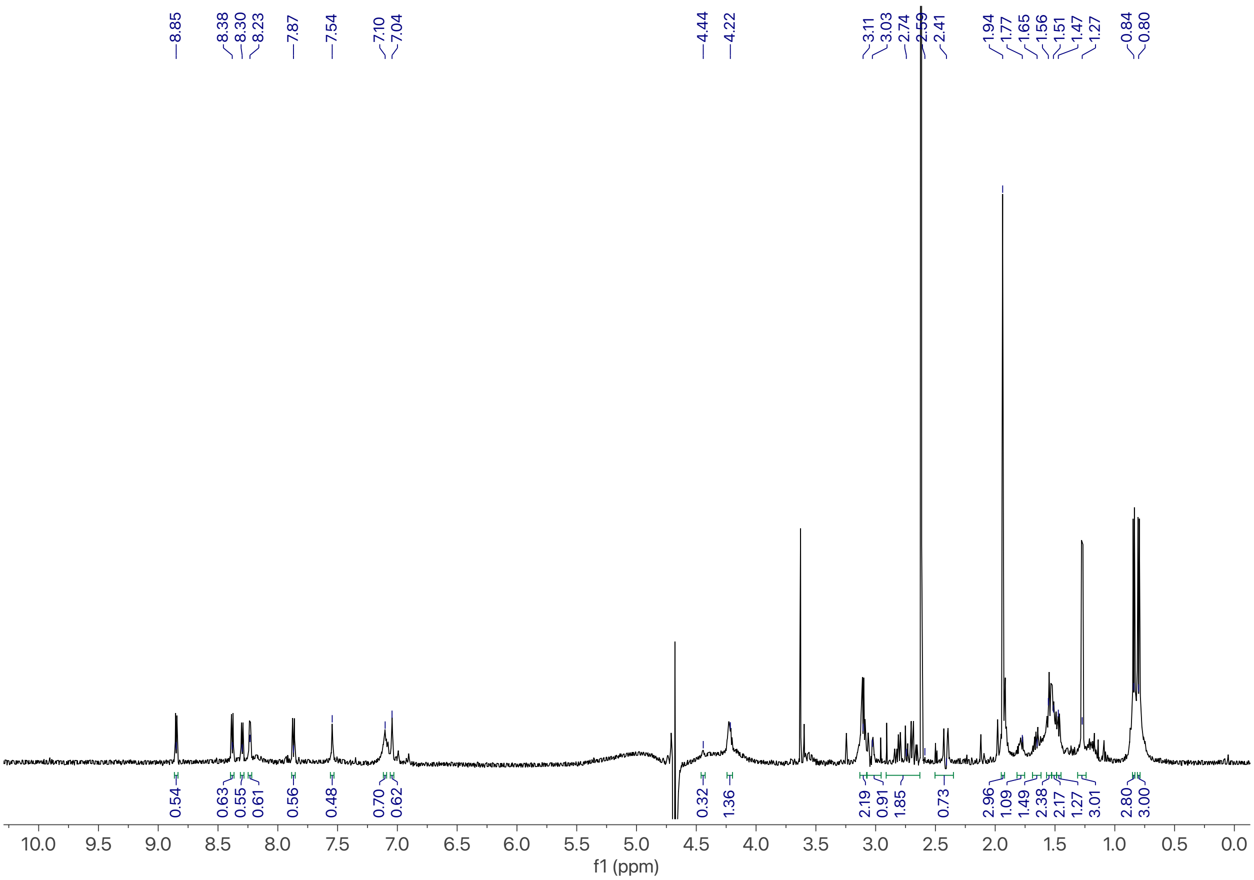

Assignment Table

Residue	Amide NH	Hα	Hβ	Hγ	other
**Leu1**	8.23 (d, *J* = 5.8 Hz)	4.22	1.51	1.47	δ: 0.84, 0.80, Ac: 1.94
**D-Pra2**	8.85 (d, *J* = 6.2 Hz)	4.44	3.03, 2.41		
**Ala3**	7.86 (d, *J* = 8.9 Hz)	4.51	1.27		
**D-Pra4**	8.38 (d, *J* = 7.8 Hz)	4.66	2.74		
**Arg5**	8.30 (d, *J* = 7.5 Hz)	4.25	1.77, 1.66	1.56	δ: 3.11, ε: 7: 10, NH: 7.54, 7.04

Compound 6: Model Peptide D

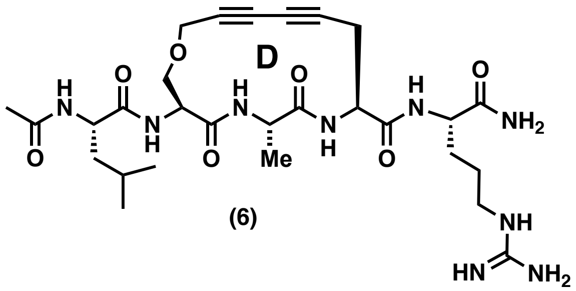

(6)

Chemical Formula: $C_{28}H_{43}N_9O_7$

Molecular Weight: 617.71

The product was purified by mass-directed prep LC. The crude material was purified on a Waters Autopurification LC with a Waters BEH C18 column (5 um, 19x160 mm) using under isocratic conditions (15% B - acetonitrile / 85% A - 0.1% aqueous trifluoroacetic acid; 30 mL/min for 15 minutes) at ambient temperature. Fractionation was triggered by a Waters QDa single quadrupole mass spec (ESI+).
Retention time: 7.4 min

HRMS:

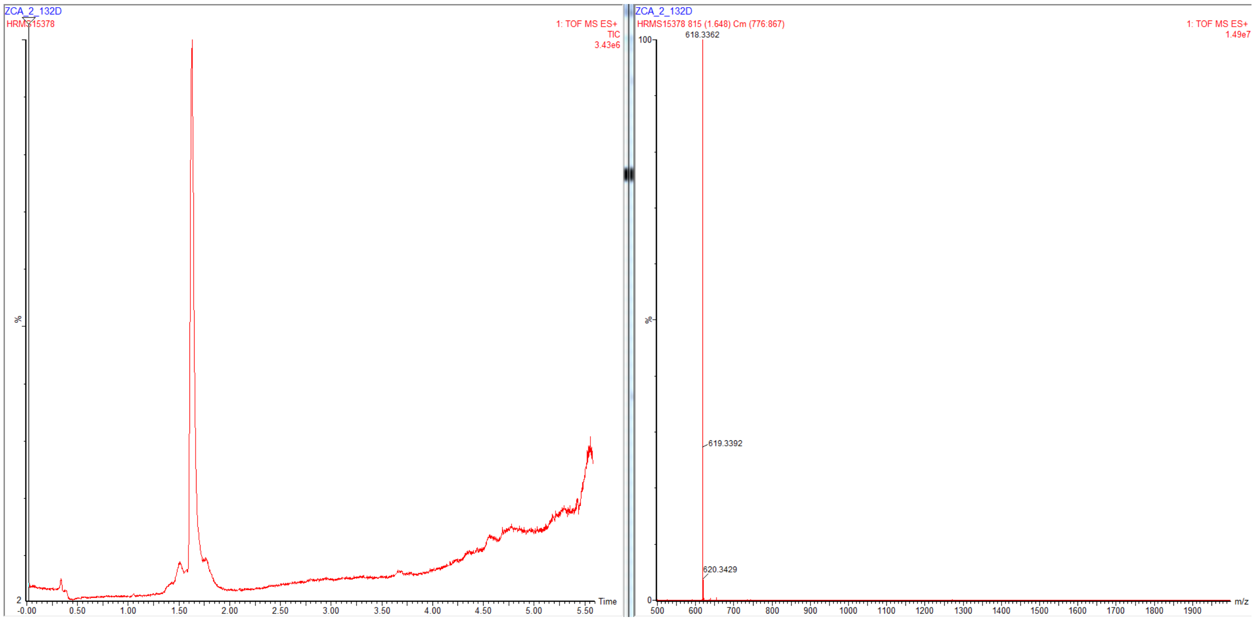

^1H Spectrum

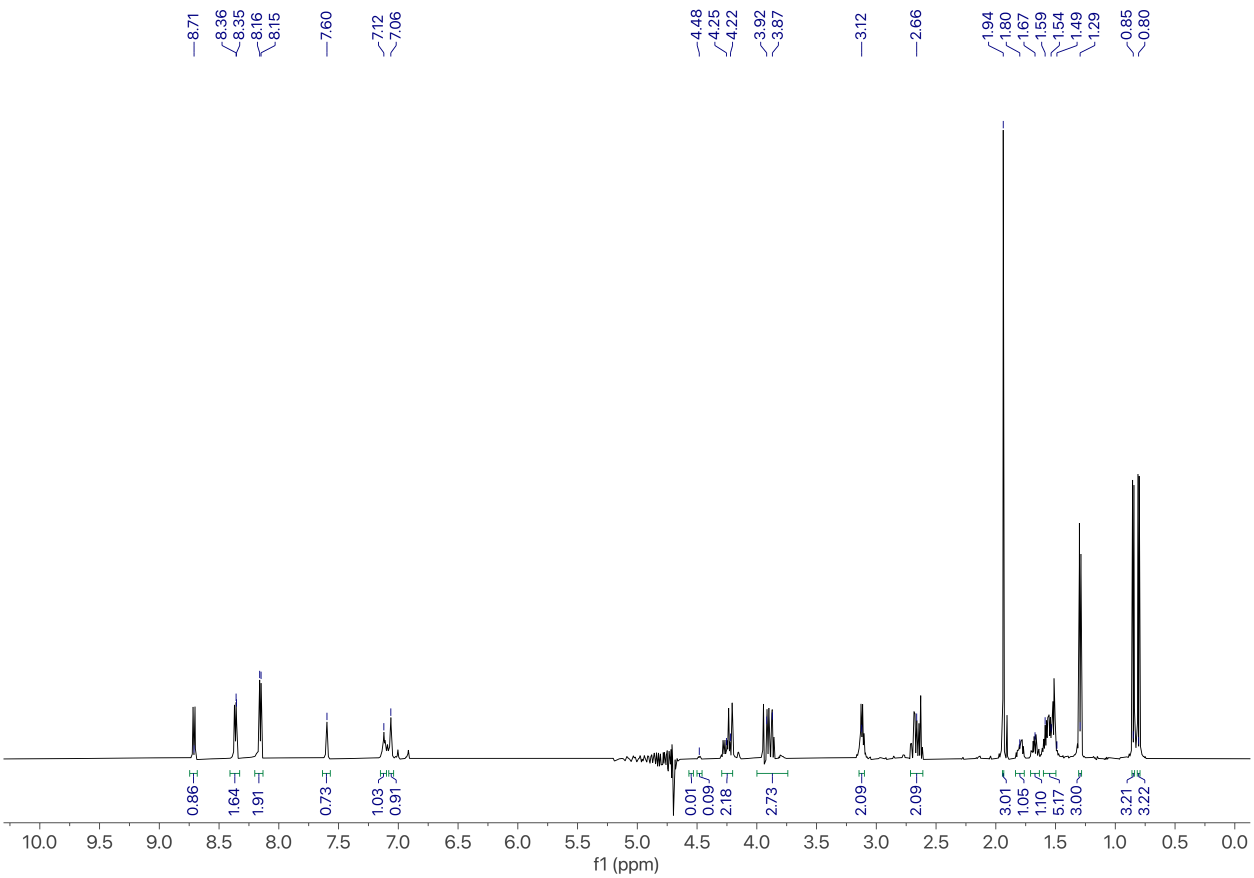

Assignment Table

Residue	Amide NH	Hα	Hβ	Hγ	other
**Leu1**	8.16 (d, *J* = 7.0 Hz)	4.25	1.49	1.59	δ: 0.85, 0.80, Ac: 1.94
**Prs2**	8.15 (d, *J* = 7.0 Hz)	4.48	3.87		δ: 3.92
**Ala3**	8.36 (d, *J* = 8.4 Hz)	4.55	1.29		
**Pra4**	8.71 (d, *J* = 9.6 Hz)	4.84	2.66		
**Arg5**	8.35 (d, *J* = 7.1 Hz)	4.22	1.80, 1.67	1.54	δ: 3.12, ε: 7.12, NH: 7.60, 7.06

Compound 7: Model Peptide E

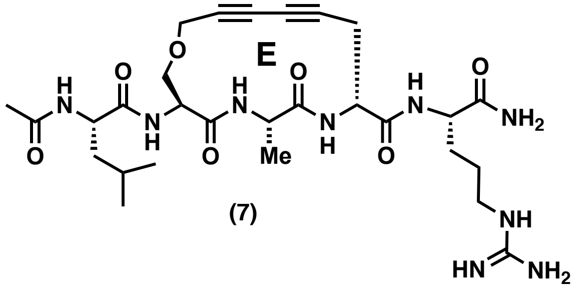

Chemical Formula: $C_{28}H_{43}N_9O_7$

Molecular Weight: 617.71

The product was purified by mass-directed prep LC. The crude material was purified on a Waters Autopurification LC with a Waters BEH C18 column (5 um, 19x160 mm) using a 0.1% aqueous trifluoroacetic acid:acetonitrile gradient (30 mL/min, main segment of gradient at 15-35% acetonitrile over 8 minutes) at ambient temperature. Fractionation was triggered by a Waters QDa single quadrupole mass spec (ESI+).
Retention time: 4.9 min

HRMS:

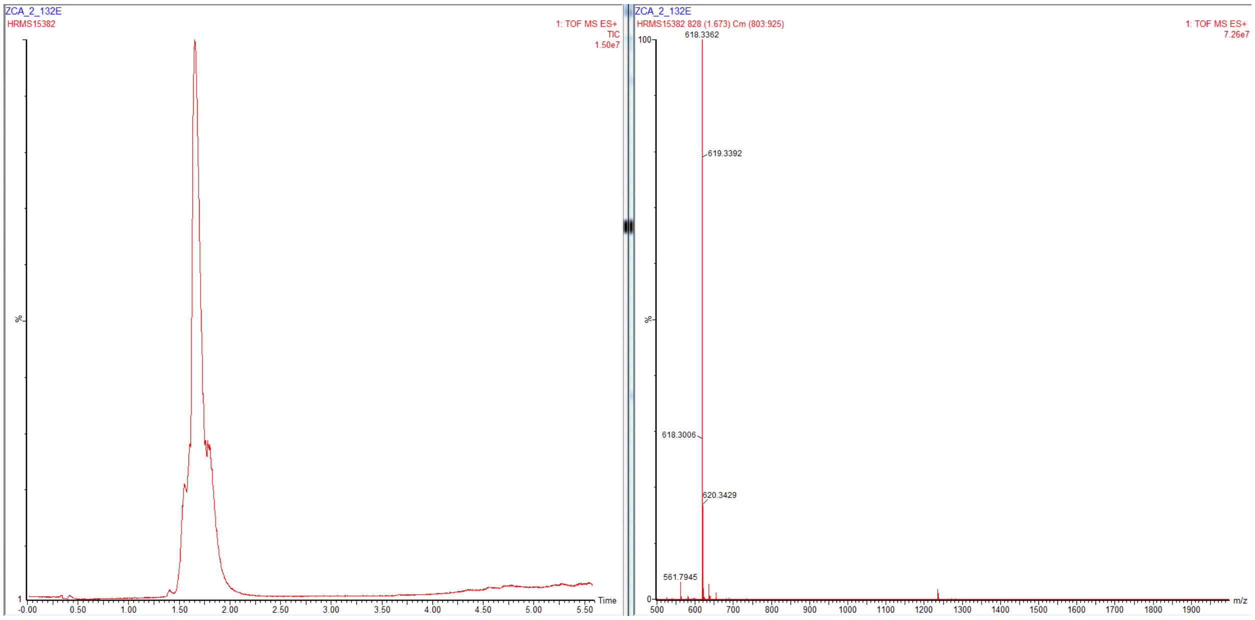

^{1}H Spectrum

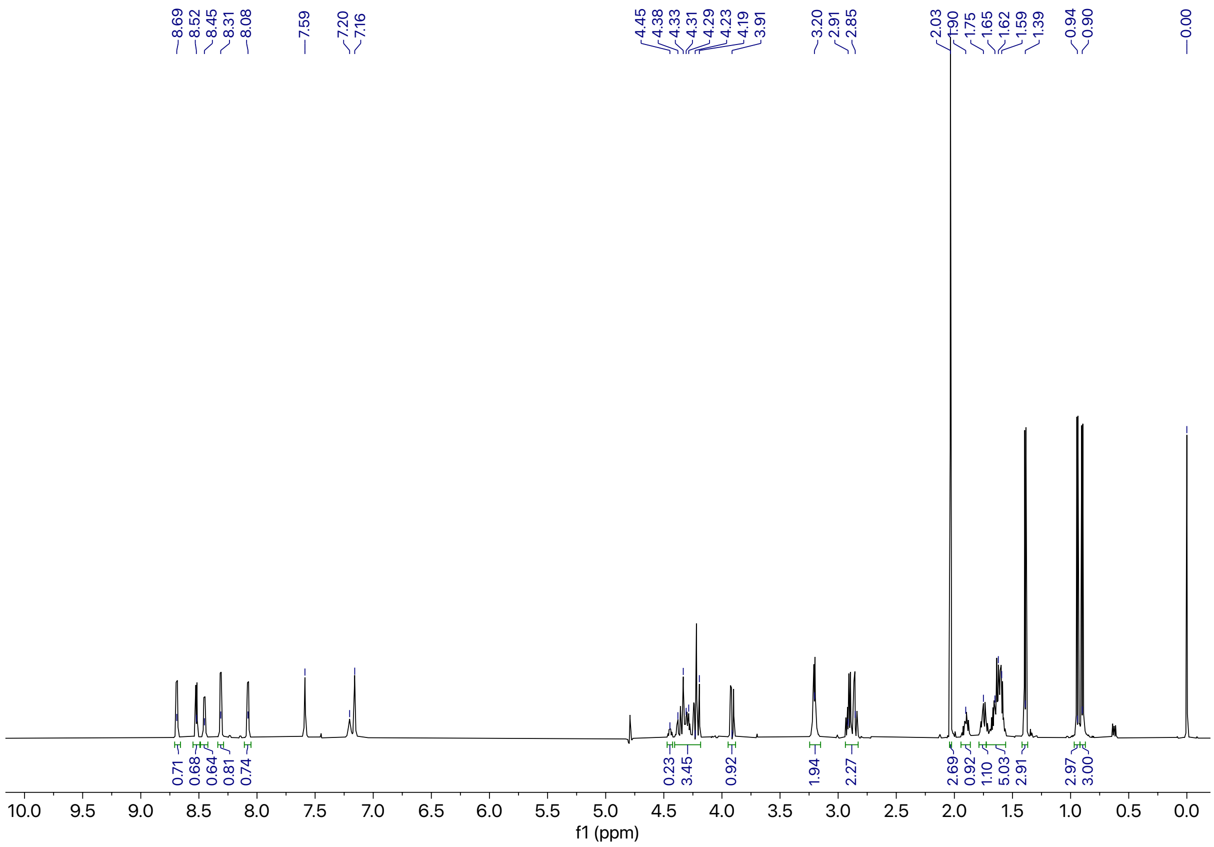

Assignment Table

Residue	Amide NH	Hα	Hβ	Hγ	other
**Leu1**	8.31 (d, *J* = 6.3 Hz)	4.29	1.67	1.59	δ: 0.94, 0.90, Ac: 2.03
**Prs2**	8.69 (d, *J* = 7.6 Hz)	4.78	3.91, 4.23		δ: 4.19, 4.33
**Ala3**	8.08 (d, *J* = 6.9 Hz)	4.38	1.39		
**D-Pra4**	8.52 (d, *J* = 6.5 Hz)	4.45	2.85, 2.91		
**Arg5**	8.45 (d, *J* = 7.6 Hz)	4.31	1.90, 1.75	1.62	δ: 3.20, ε: 7.20, NH: 7.59, 7.16

Compound 8: Model Peptide F

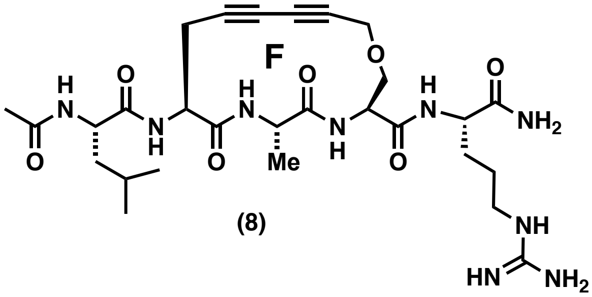

(8)

Chemical Formula: $C_{28}H_{43}N_9O_7$

Molecular Weight: 617.71

The product was purified by mass-directed prep LC. The crude material was purified on a Waters Autopurification LC with a Waters BEH C18 column (5 um, 19x160 mm) using under isocratic conditions (15% B - acetonitrile / 85% A - 0.1% aqueous trifluoroacetic acid; 30 mL/min for 17 minutes) at ambient temperature. Fractionation was triggered by a Waters QDa single quadrupole mass spec (ESI+).
Retention time: 7.0 min

HRMS:

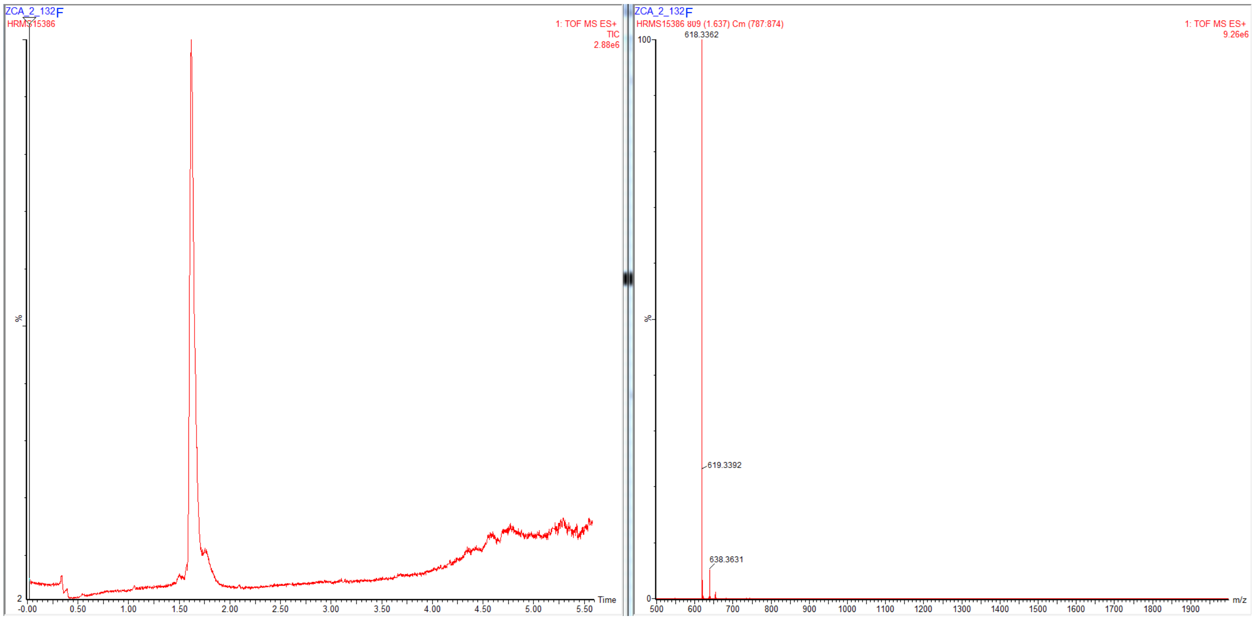

[1]H Spectrum

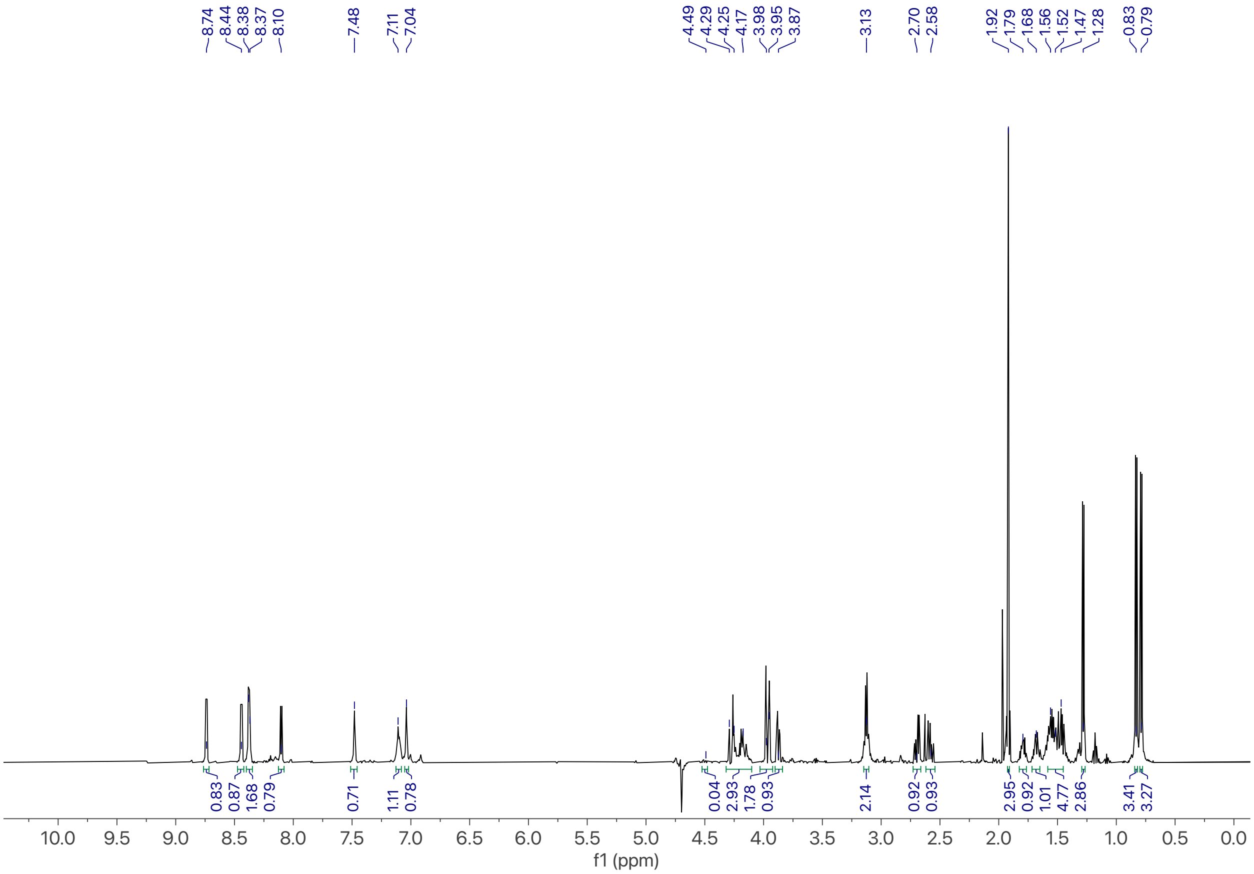

Assignment Table

Residue	Amide NH	Hα	Hβ	Hγ	other
**Leu1**	8.10 (d, *J* = 6.8 Hz)	4.17	1.47	1.52	δ: 0.83, 0.79, Ac: 1.92
**Pra2**	8.38 (d, *J* = 6.4 Hz)	4.49	2.79, 2.58		
**Ala3**	8.44 (d, *J* = 7.7 Hz)	4.55	1.28		
**Prs4**	8.74 (d, *J* = 6.9 Hz)	4.66	3.95, 3.87		δ: 4.29, 3.98
**Arg5**	8.37 (d, *J* = 7.3 Hz)	4.25	1.79, 1.68	1.56	δ: 3.13, ε: 7.11, NH: 7.48, 7.04

Compound 9: Model Peptide G

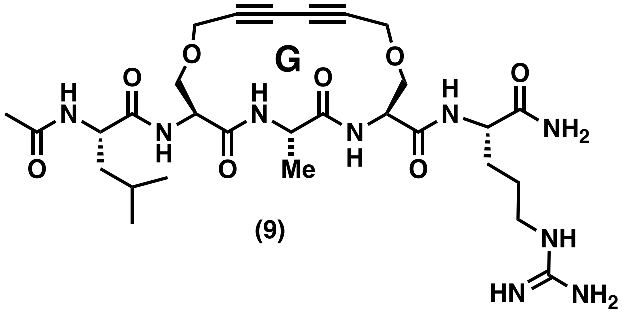

(9)

Chemical Formula: $C_{29}H_{45}N_9O_8$

Molecular Weight: 647.73

The product was purified by mass-directed prep LC. The crude material was purified on a Waters Autopurification LC with a Waters BEH C18 column (5 um, 19x160 mm) using a 0.1% aqueous trifluoroacetic acid:acetonitrile gradient (30 mL/min, main segment of gradient at 15-35% acetonitrile over 8 minutes) at ambient temperature. Fractionation was triggered by a Waters QDa single quadrupole mass spec (ESI+).
Retention time: 5.1 min

HRMS:

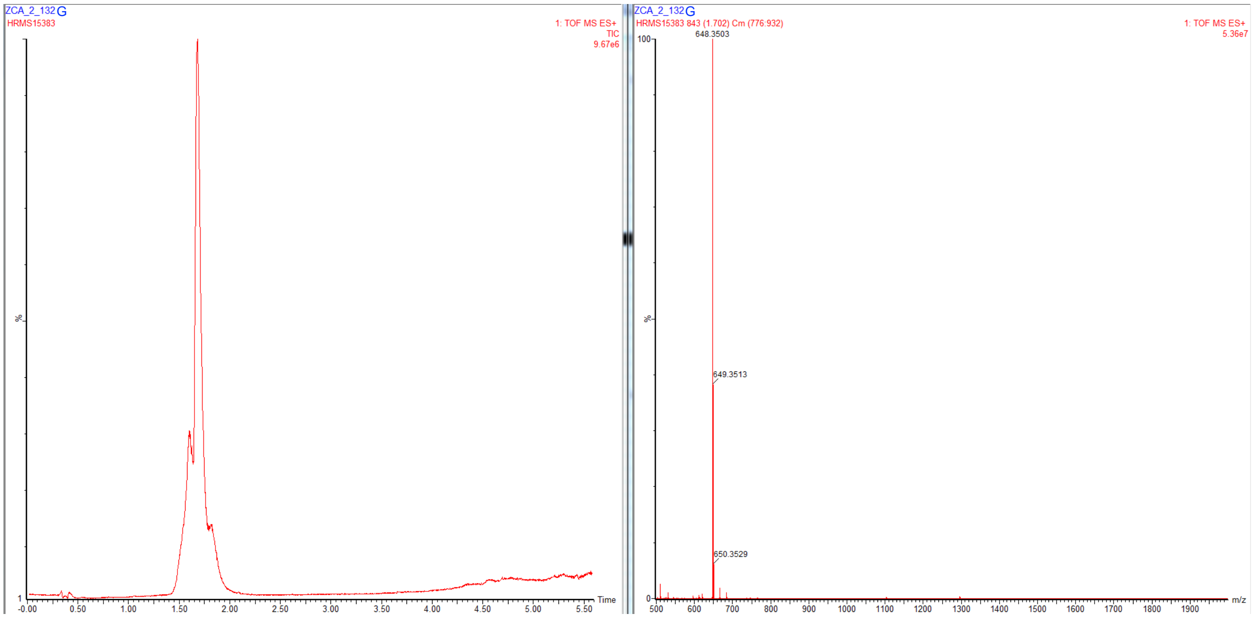

^{1}H Spectrum

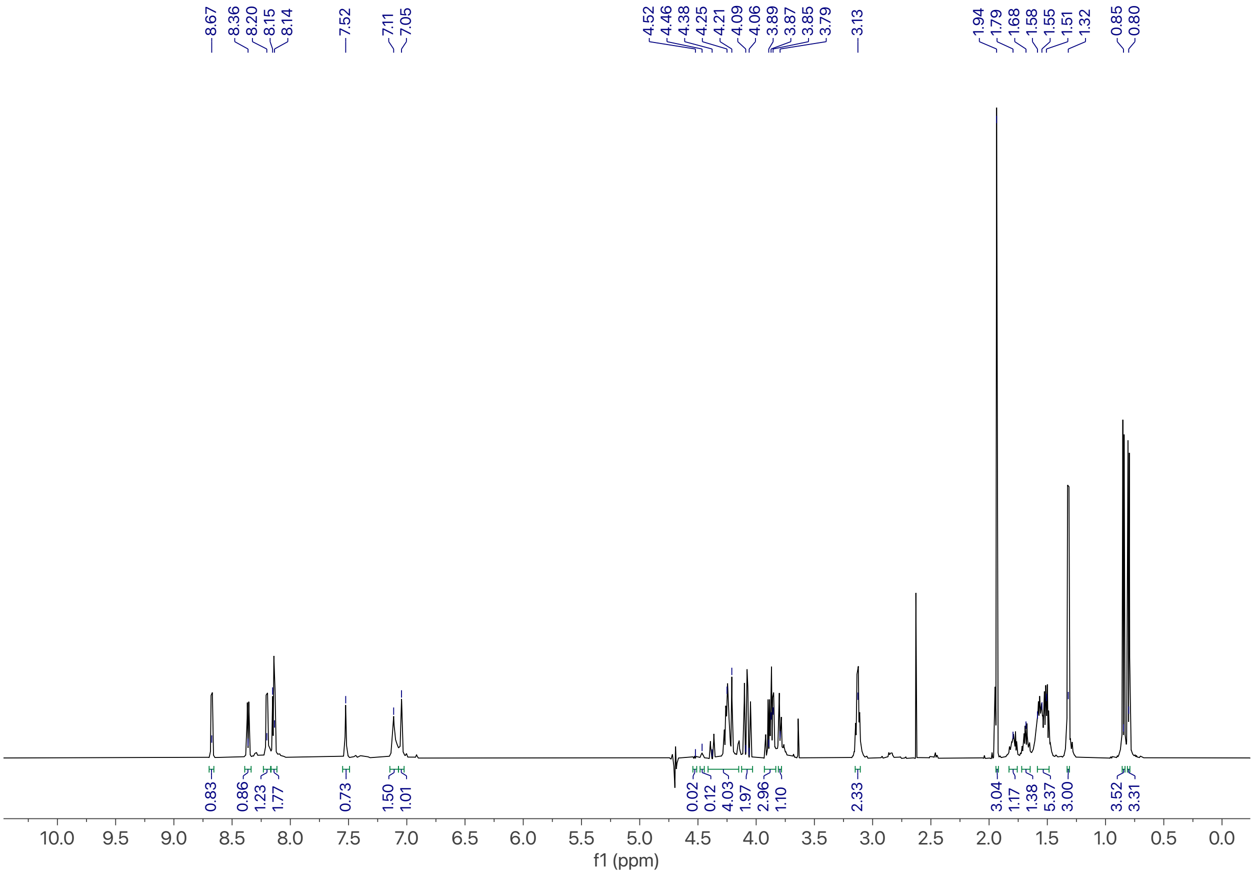

Assignment Table

Residue	Amide NH	Hα	Hβ	Hγ	other
**Leu1**	8.15 (d, *J* = 6.4 Hz)	4.25	1.51	1.58	δ: 0.85, 0.80, Ac: 1.94
**Prs2**	8.14 (d, *J* = 6.4 Hz)	4.46	3.85, 3.79		δ: 4.21, 4.06
**Ala3**	8.20 (d, *J* = 7.4 Hz)	4.52	1.32		
**Prs4**	8.67 (d, *J* = 6.8 Hz)	4.77	3.89, 3.87		δ: 4.38, 4.09
**Arg5**	8.36 (d, *J* = 7.4 Hz)	4.25	1.79, 1.68	1.55	δ: 3.13, ε: 7.11, NH: 7.52, 7.05

Alkynomycin Compounds

Compound 10: Alkynomycin A12

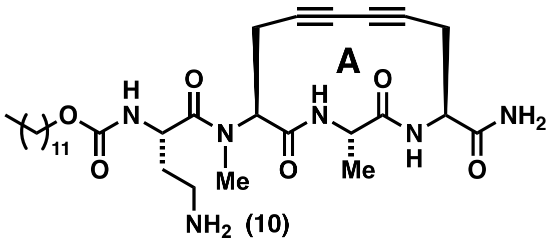

Chemical Formula: $C_{31}H_{50}N_6O_6$

Molecular Weight: 602.78

The product was purified by mass-directed prep LC. The crude material was purified on a Waters Autopurification LC with a Waters BEH C18 column (5 um, 19x160 mm) using a 0.1% aqueous trifluoroacetic acid:acetonitrile gradient (30 mL/min, main segment of gradient at 45-65% acetonitrile over 8 minutes) at ambient temperature. Fractionation was triggered by a Waters QDa single quadrupole mass spec (ESI+).
Retention time: 4.3 min

HRMS:

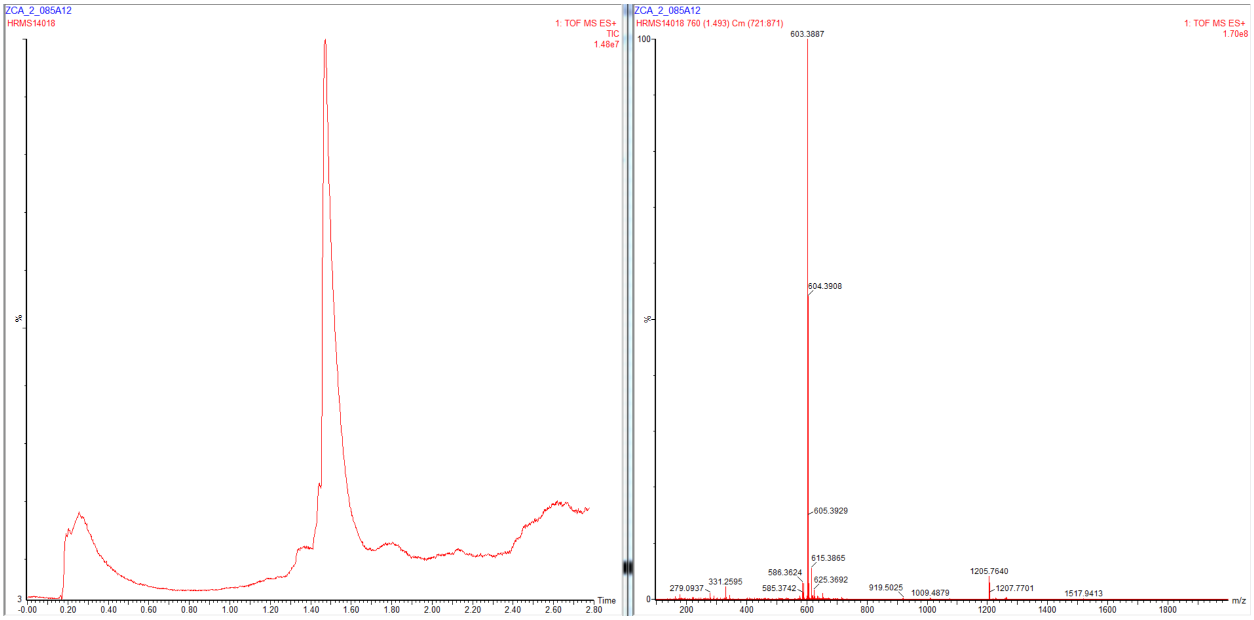

^1H Spectrum

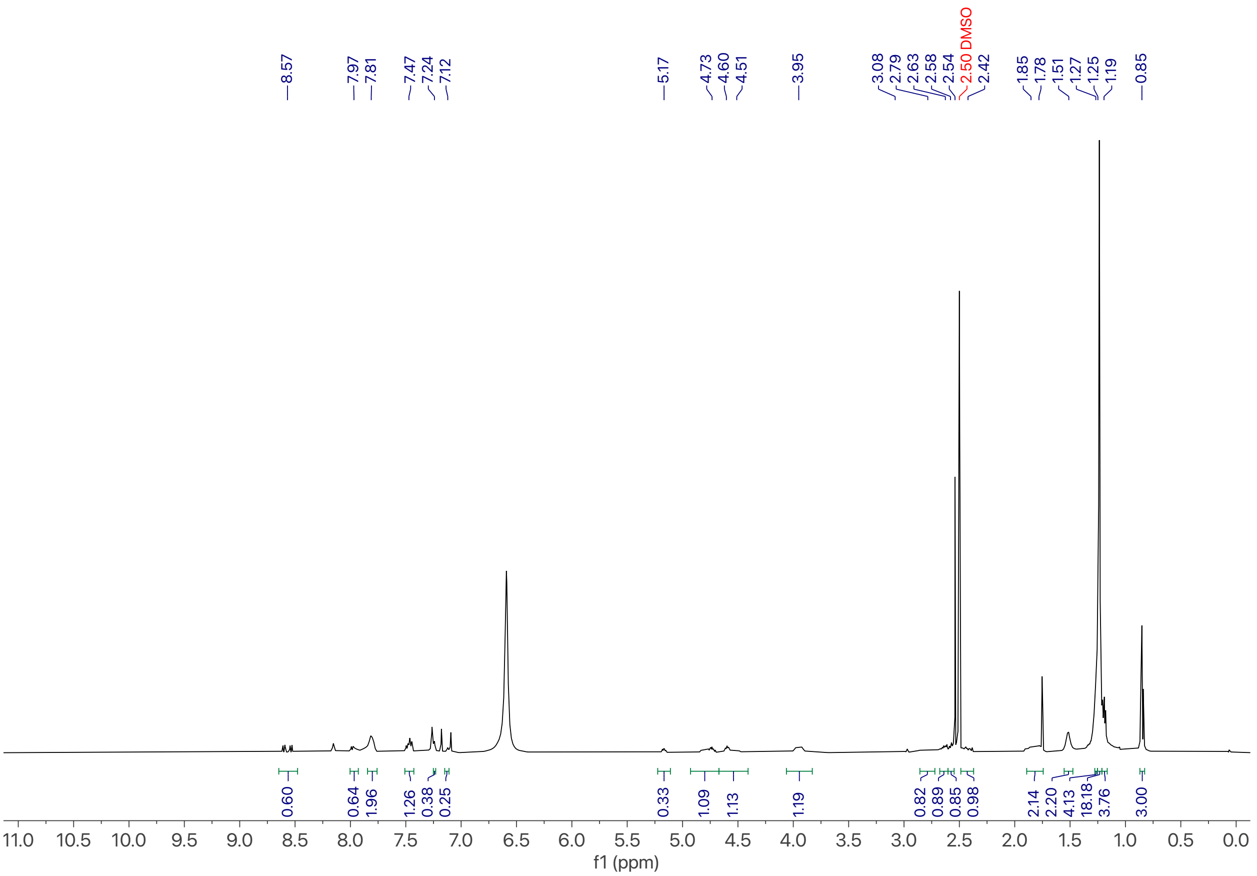

Assignment Table

Residue	Amide NH	Hα	Hβ	Hγ	other
**Lipid**		3.95	1.51	1.27	C4-11: 1.24, C12: 0.85
**Dab1**	7.49 (d, *J* = 8.8 Hz)	4.51	1.85, 1.78	2.85, 2.72	NH: 7.81, 7.12
**Pra2**		5.17	2.79, 2.42		NMe: 3.08
**Ala3**	7.97 (d, *J* = 9.0 Hz)	4.60	1.19		
**Pra4**	8.57 (d, *J* = 10.2 Hz)	4.73	2.63, 2.58		NH: 7.24, 7.47

Chemical shifts for Alkynomycin **A12** were assigned using ^1H, TOCSY, ROESY, ^{13}C-HSQC, ^{13}C-HMBC data.

Note: All N-methylated compounds, A12-H16, showed *cis* and *trans* isomers in the NMR timescale. Isomers were resolved with reference to exchangeable sets of protons. For example, exchange between the Hα protons of *cis* and *trans* Dab1 (4.51 ppm, 4.84 ppm) and *cis* and *trans* Pra2 (5.17, 4.81 ppm) were observed in the ROE spectrum of A12. The major isomer is reported for all compounds.

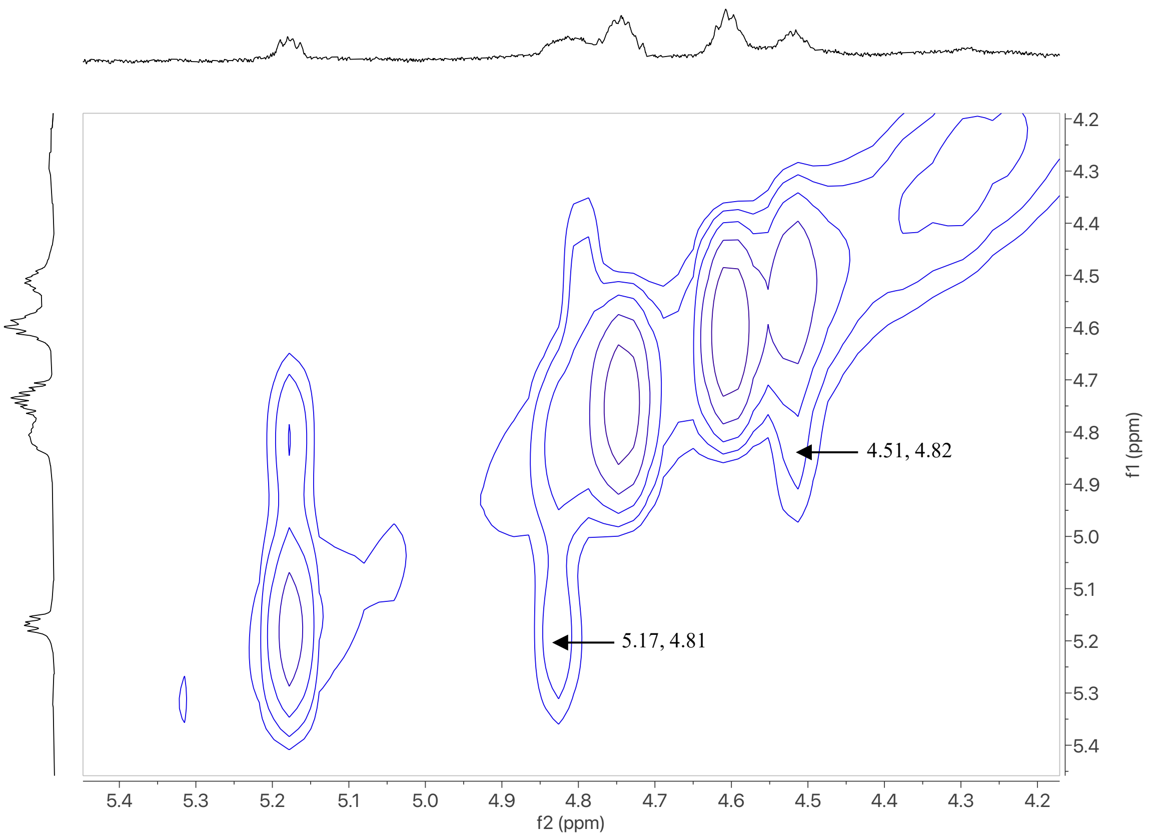

Compound S1: Alkynomycin B12

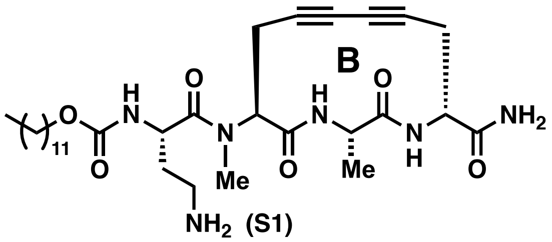

Chemical Formula: $C_{31}H_{50}N_6O_6$

Molecular Weight: 602.78

The product was purified by mass-directed prep LC. The crude material was purified on a Waters Autopurification LC with a Waters BEH C18 column (5 um, 19x160 mm) using under isocratic conditions (40% B - acetonitrile / 60% A - 0.1% aqueous trifluoroacetic acid; 30 mL/min for 13 minutes) at ambient temperature. Fractionation was triggered by a Waters QDa single quadrupole mass spec (ESI+).
Retention time: 11.2 min

HRMS:

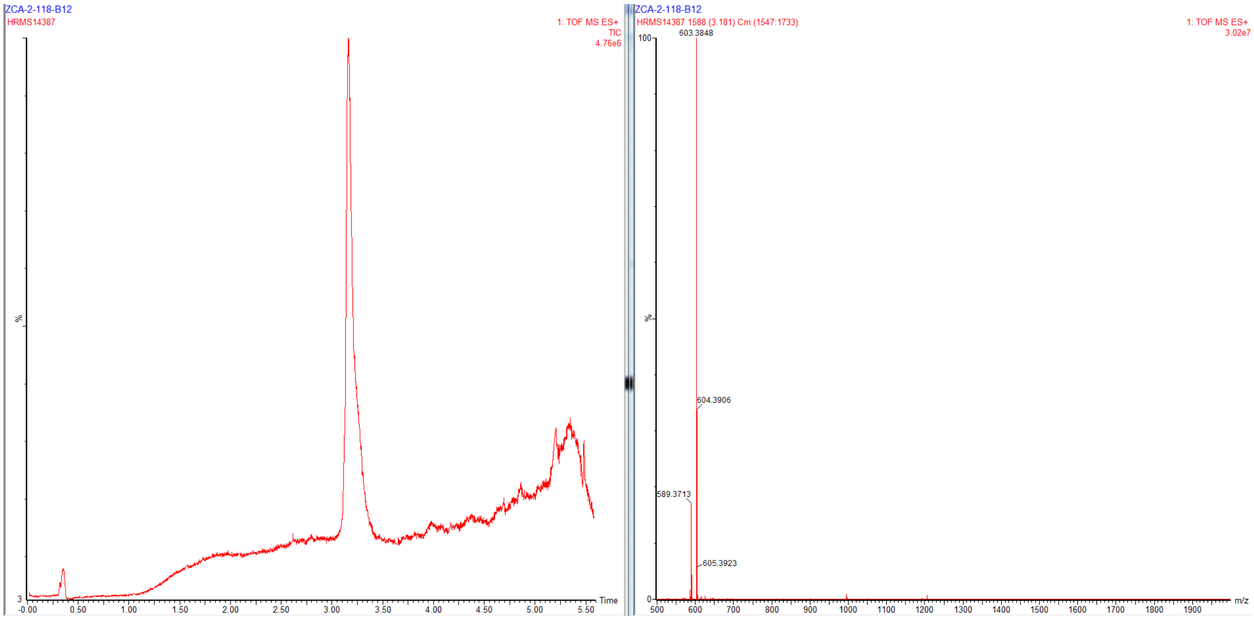

[1]H Spectrum

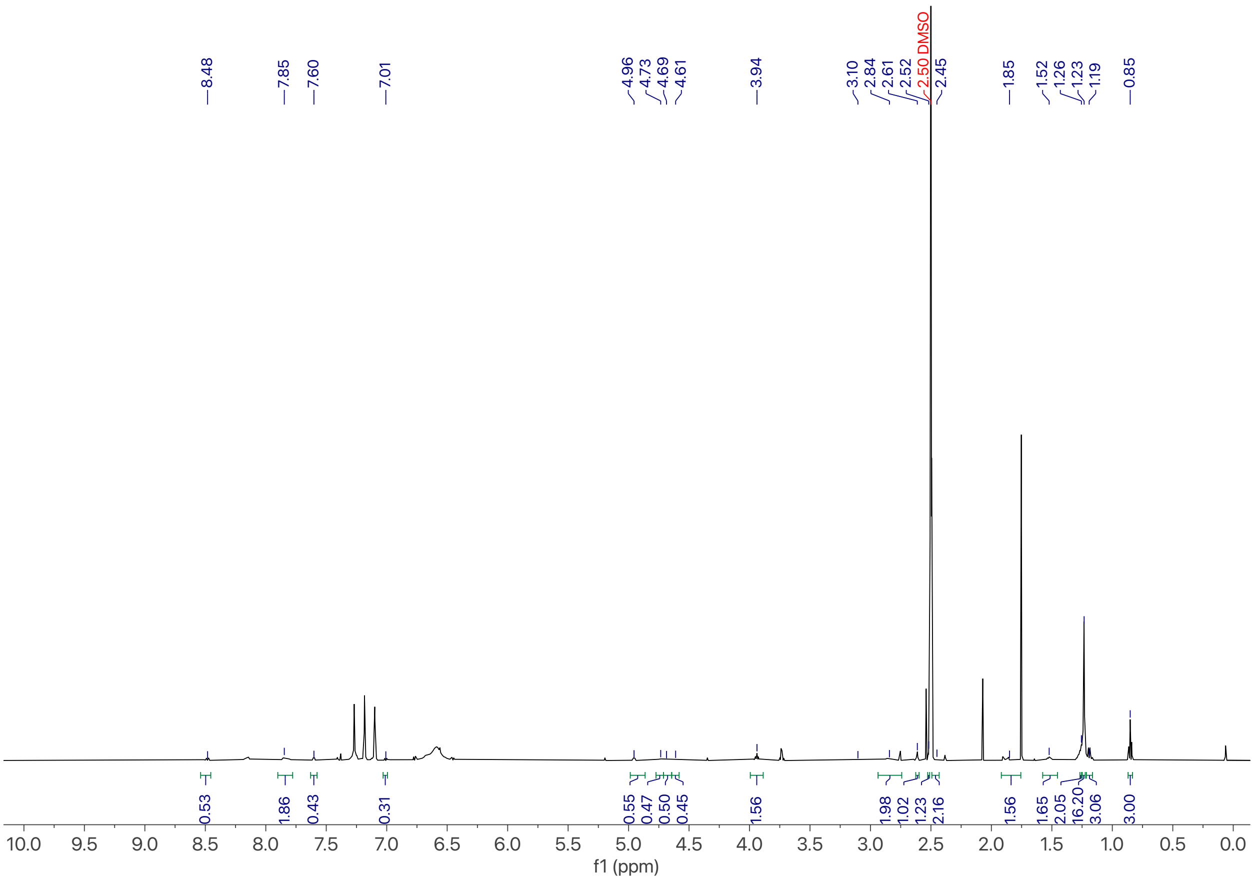

Assignment Table

Residue	Amide NH	Hα	Hβ	Hγ	other
**Lipid**		3.95	1.52	1.26	C4-11: 1.24, C12: 0.85
**Dab1**	7.61 (d, J = 6.9 Hz)	4.61	1.85	2.86	NH: 7.85
**Pra2**		4.96	2.47		NMe: 3.10
**Ala3**	7.02 (d, J = 9.6 Hz)	4.69	1.19		
**D-Pra4**	8.49 (d, J = 10.3 Hz)	4.73	2.61, 2.52		

Compound 12: Alkynomycin C12

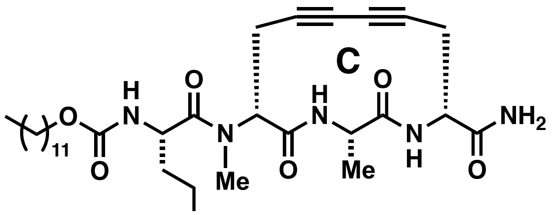

(12)

Chemical Formula: $C_{31}H_{50}N_6O_6$

Molecular Weight: 602.78

The product was purified by mass-directed prep LC. The crude material was purified on a Waters Autopurification LC with a Waters BEH C18 column (5 um, 19x160 mm) using under isocratic conditions (45% B - acetonitrile / 55% A - 0.1% aqueous trifluoroacetic acid; 30 mL/min for 11 minutes) at ambient temperature. Fractionation was triggered by a Waters QDa single quadrupole mass spec (ESI+).
Retention time: 5.7 min

HRMS:

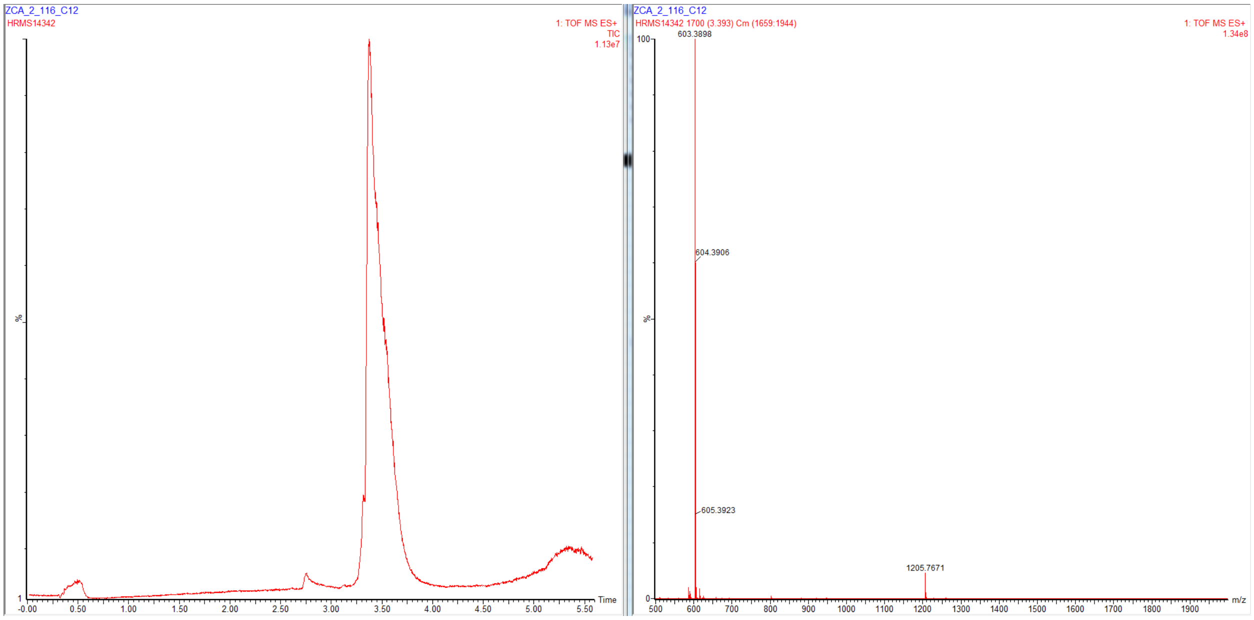

^{1}H Spectrum

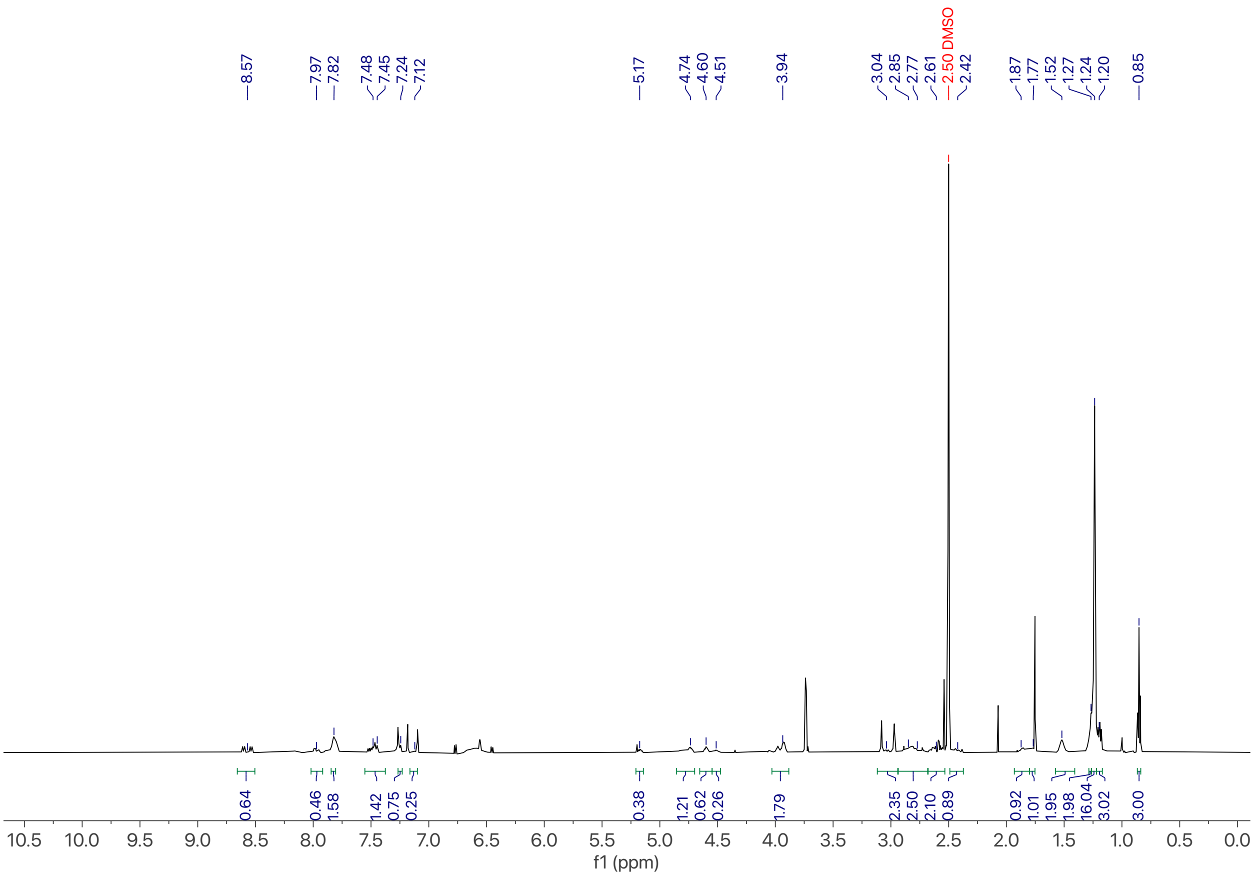

Assignment Table

Residue	Amide NH	Hα	Hβ	Hγ	other
**Lipid**		3.94	1.51	1.27	C4-11: 1.24, C12: 0.85
**Dab1**	7.48	4.51	1.87, 1.78	2.83	NH: 7.82, 7.12
**D-Pra2**		5.17	2.77, 2.42		NMe: 3.08
**Ala3**	7.99 (d, *J* = 8.5 Hz)	4.60	1.20		
**D-Pra4**	8.57 (d, *J* = 9.8 Hz)	4.74	2.61		NH: 7.45, 7.24

Compound S3: Alkynomycin D12

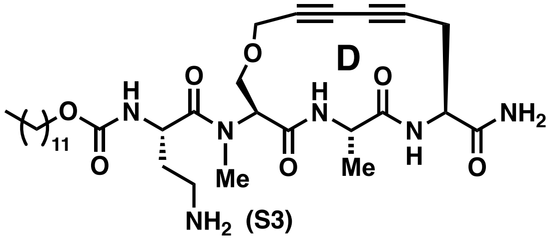

Chemical Formula: $C_{32}H_{52}N_6O_7$

Molecular Weight: 632.80

The product was purified by mass-directed prep LC. The crude material was purified on a Waters Autopurification LC with a Waters BEH C18 column (5 um, 19x160 mm) using under isocratic conditions (45% B - acetonitrile / 55% A - 0.1% aqueous trifluoroacetic acid; 30 mL/min for 12 minutes) at ambient temperature. Fractionation was triggered by a Waters QDa single quadrupole mass spec (ESI+).
Retention time: 5.2 min

HRMS:

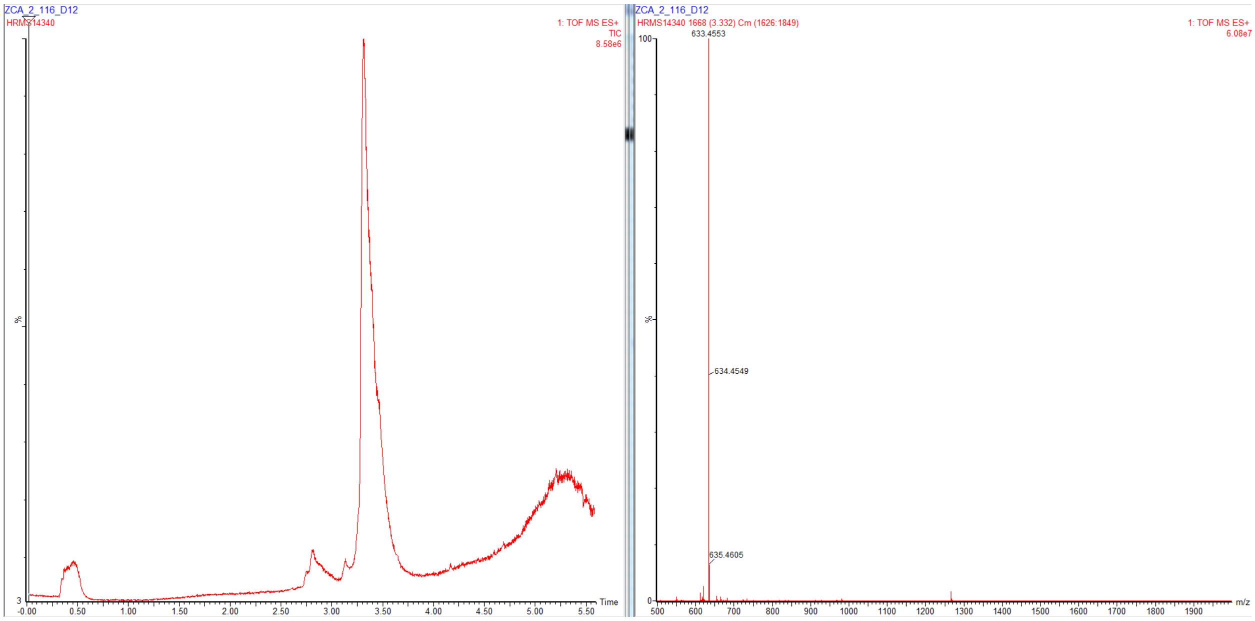

[1]H Spectrum

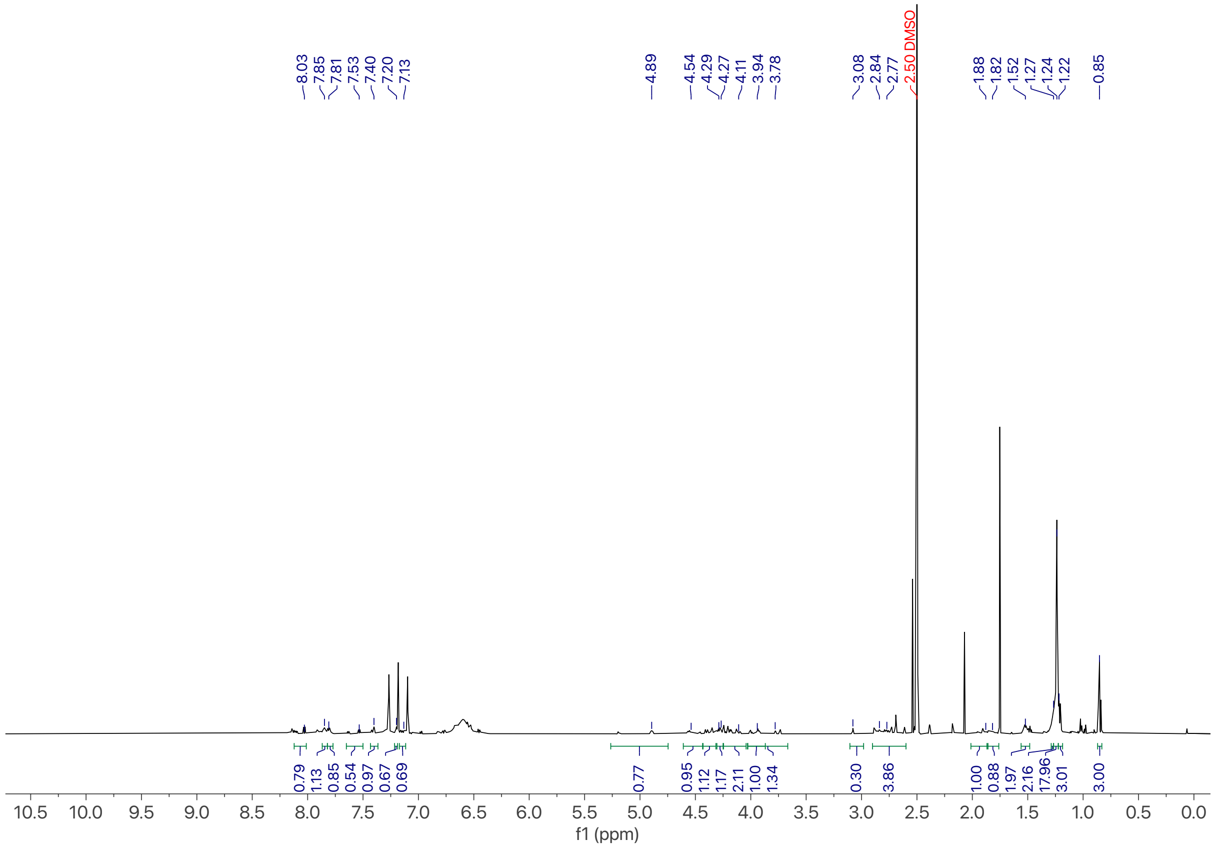

Assignment Table

Residue	Amide NH	Hα	Hβ	Hγ	other
**Lipid**		3.94	1.52	1.27	C4-11: 1.24, C12: 0.85
**Dab1**	7.53 (d, *J* = 8.1 Hz)	4.54	1.88, 1.82	2.84	NH: 7.85, 7.13
**Prs2**		4.89	3.78		δ: 4.11, NMe: 3.08
**Ala3**	7.81 (d, *J* = 9.2 Hz)	4.29	1.22		
**Pra4**	8.03 (d, *J* = 8.1 Hz)	4.27	2.77		NH: 7.40, 7.20

Compound S5: Alkynomycin E12

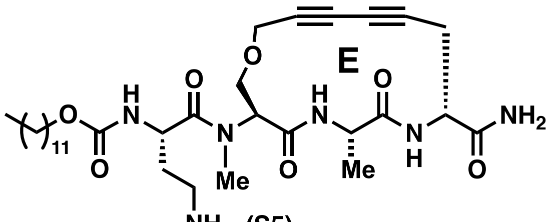

(S5)

Chemical Formula: $C_{32}H_{52}N_6O_7$

Molecular Weight: 632.80

The product was purified by mass-directed prep LC. The crude material was purified on a Waters Autopurification LC with a Waters BEH C18 column (5 um, 19x160 mm) using under isocratic conditions (45% B - acetonitrile / 55% A - 0.1% aqueous trifluoroacetic acid; 30 mL/min for 15 minutes) at ambient temperature. Fractionation was triggered by a Waters QDa single quadrupole mass spec (ESI+).
Retention time: 6.2 min

HRMS:

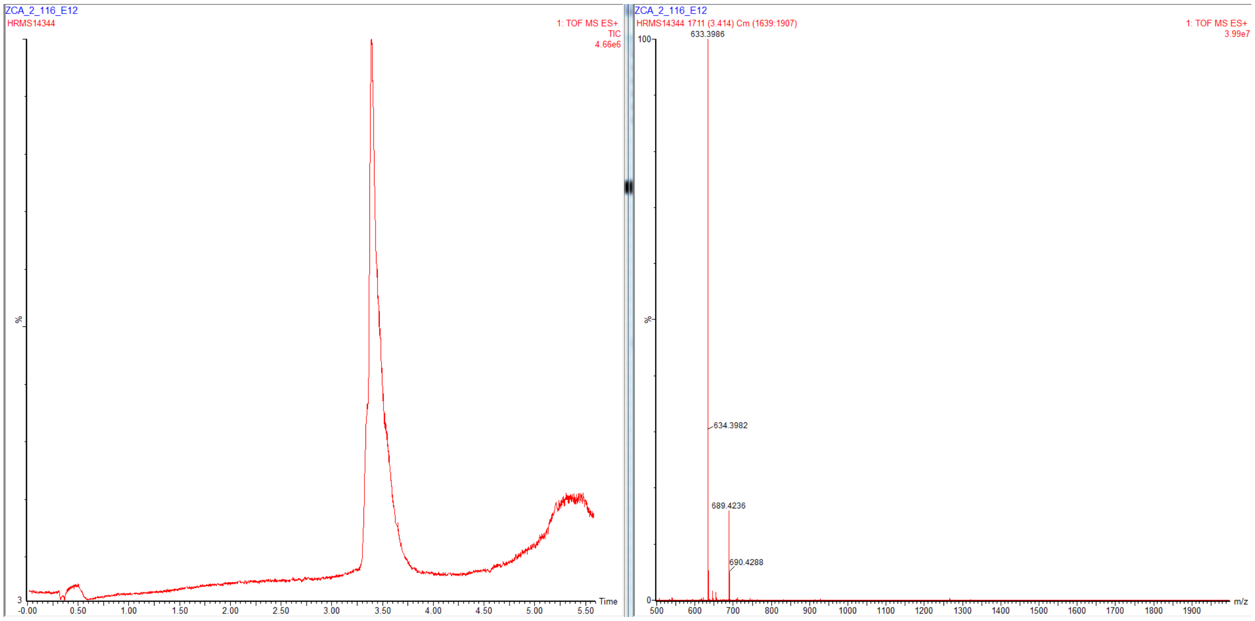

[1]H Spectrum

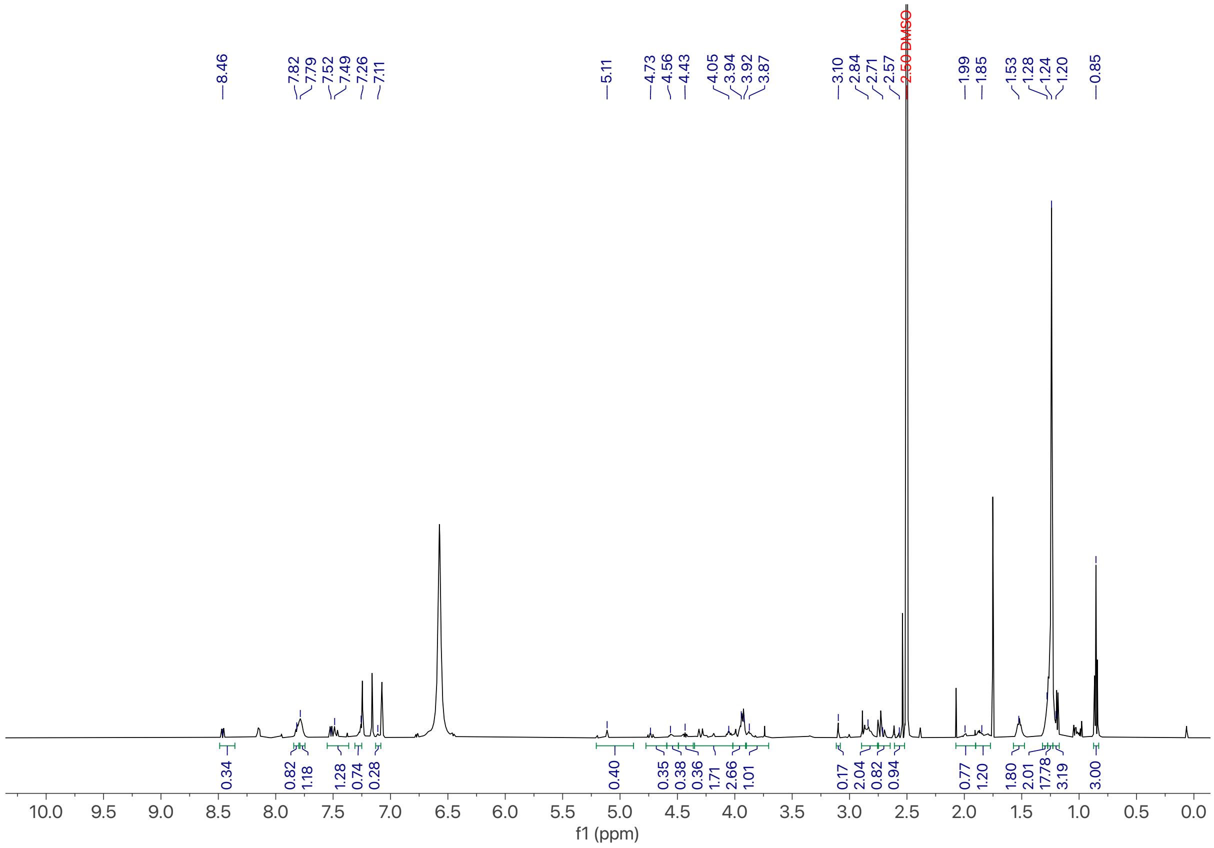

Assignment Table

Residue	Amide NH	Hα	Hβ	Hγ	other
**Lipid**		3.94	1.53	1.28	C4-11: 1.24, C12: 0.85
**Dab1**	7.52 (d, *J* = 8.3 Hz)	4.56	1.99, 1.85	2.85	NH: 7.79, 7.11
**Prs2**		5.11	3.87, 3.92		δ: 4.05, NMe: 3.11
**Ala3**	7.82 (d, *J* = 7.3 Hz)	4.43	1.20		
**D-Pra4**	8.45 (d, *J* = 10.0 Hz)	4.73	2.71, 2.57		NH: 7.49, 7.26

Compound 14: Alkynomycin F12

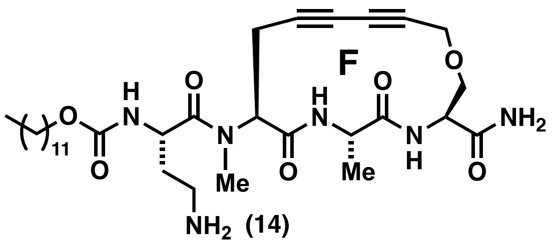

(14)

Chemical Formula: $C_{32}H_{52}N_6O_7$

Molecular Weight: 632.80

The product was purified by mass-directed prep LC. The crude material was purified on a Waters Autopurification LC with a Waters BEH C18 column (5 um, 19x160 mm) using under isocratic conditions (45% B - acetonitrile / 55% A - 0.1% aqueous trifluoroacetic acid; 30 mL/min for 15 minutes) at ambient temperature. Fractionation was triggered by a Waters QDa single quadrupole mass spec (ESI+).
Retention time: 6.5 min

HRMS:

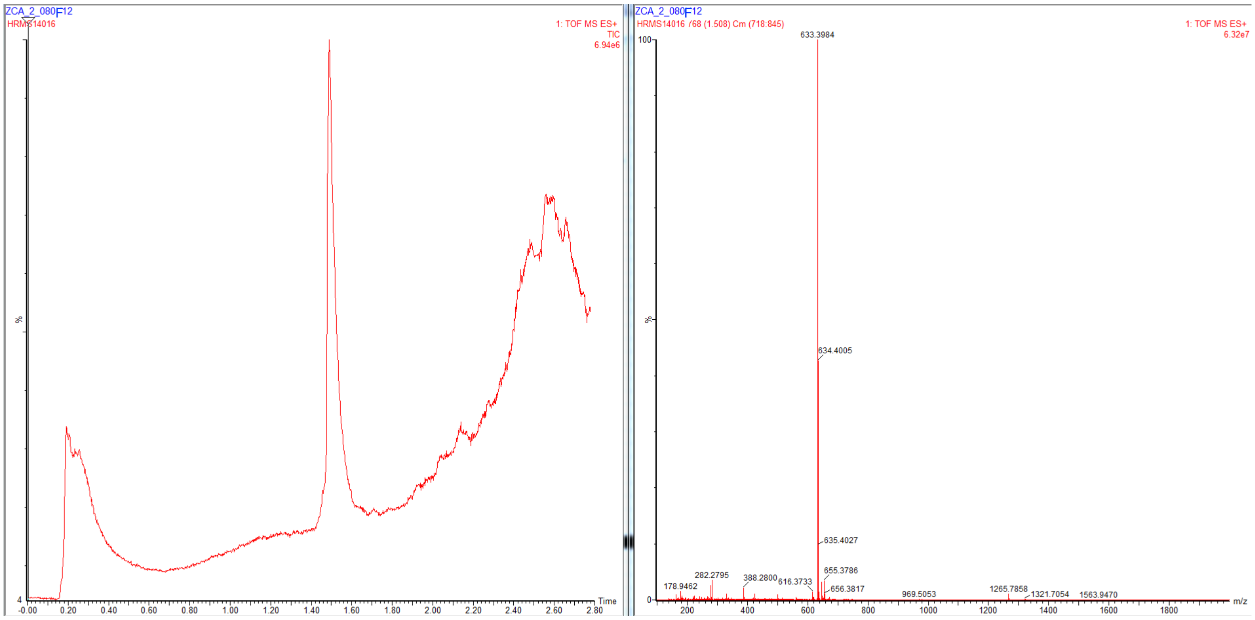

[1]H Spectrum

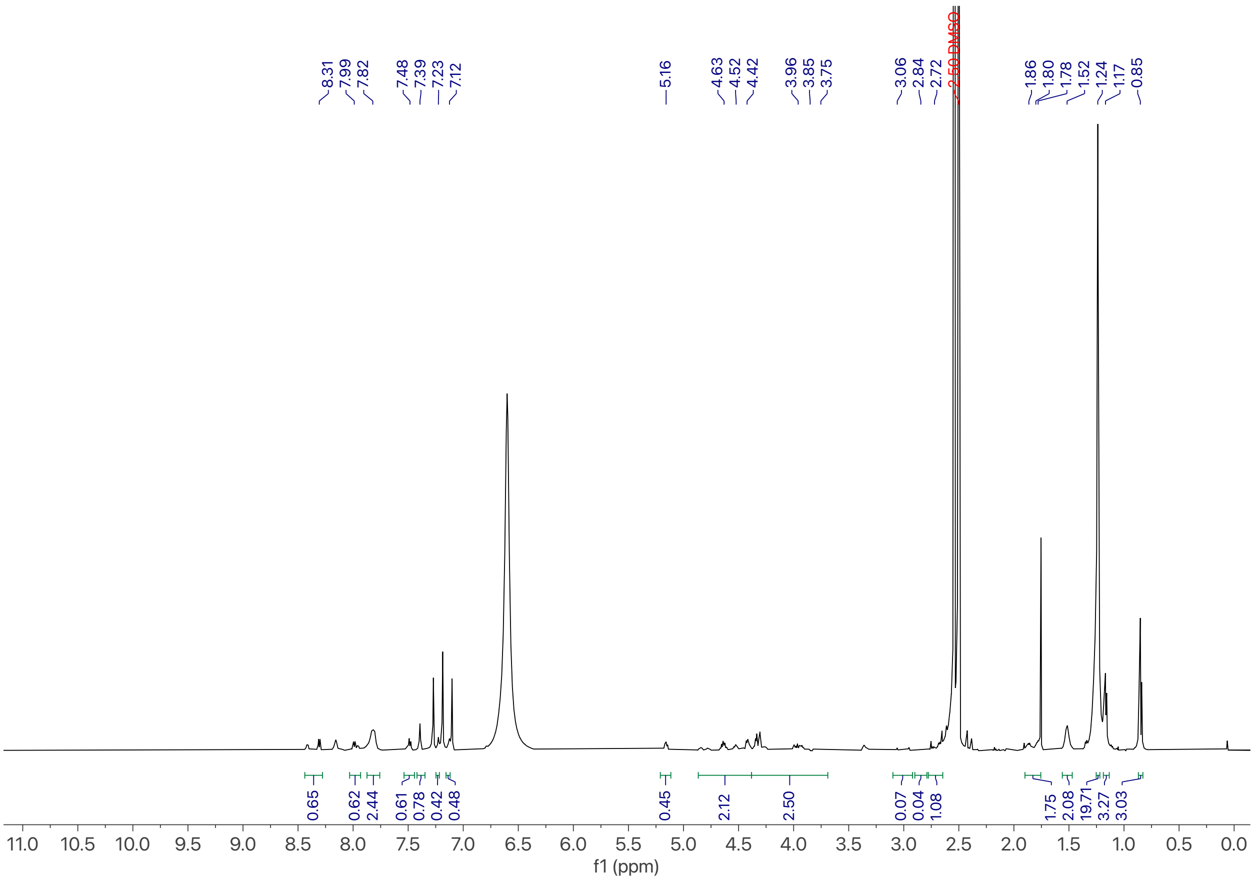

Assignment Table

Residue	Amide NH	Hα	Hβ	Hγ	other
**Lipid**		3.96	1.52	1.24	C4-11: 1.24, C12: 0.85 (t)
**Dab1**	7.48 (d, *J* = 8.6 Hz)	4.53	1.84, 1.72	2.84	NH: 7.82, 7.12
**Pra2**		5.16	2.73		NMe: 3.06
**Ala3**	7.99 (d, *J* = 8.3 Hz)	4.63	1.17		
**Prs4**	8.31 (d, *J* = 7.6 Hz)	4.42	3.85, 3.76		NH: 7.39, 7.23

Compound S7: Alkynomycin G12

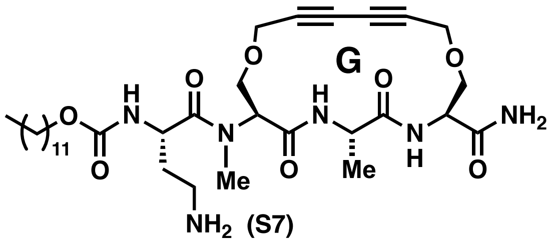

(S7)

Chemical Formula: $C_{33}H_{54}N_6O_8$

Molecular Weight: 662.83

The product was purified by mass-directed prep LC. The crude material was purified on a Waters Autopurification LC with a Waters BEH C18 column (5 um, 19x160 mm) using under isocratic conditions (45% B - acetonitrile / 55% A - 0.1% aqueous trifluoroacetic acid; 30 mL/min for 15 minutes) at ambient temperature. Fractionation was triggered by a Waters QDa single quadrupole mass spec (ESI+).
Retention time: 7.1 min

HRMS:

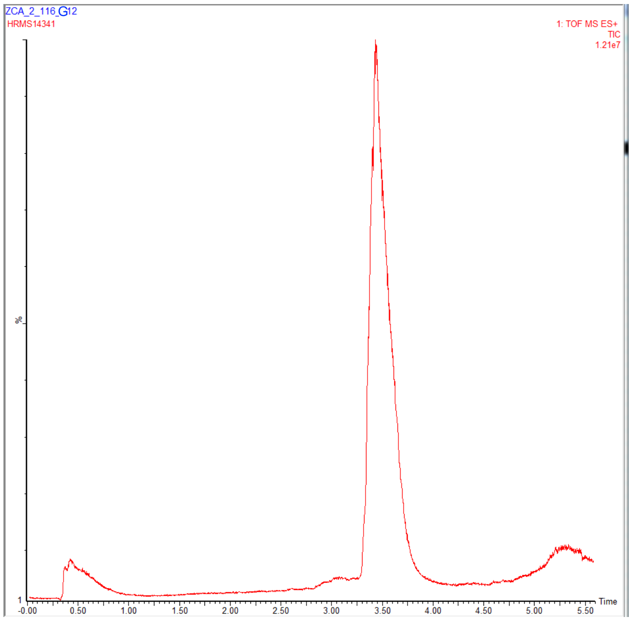

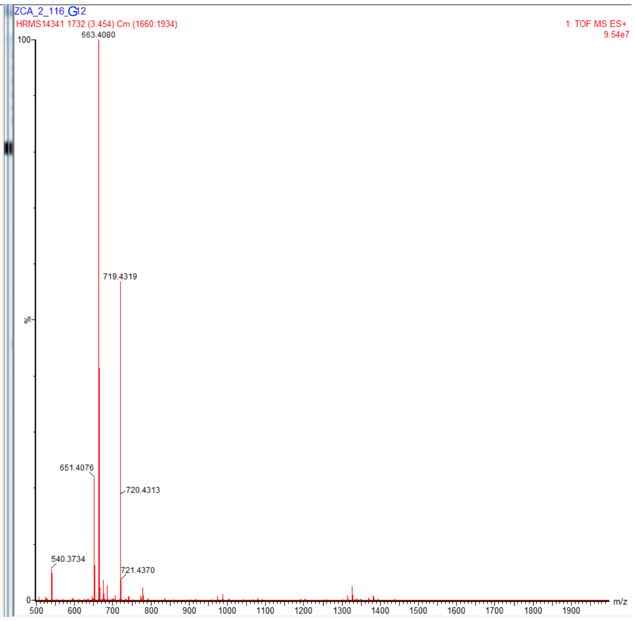

[1]H Spectrum

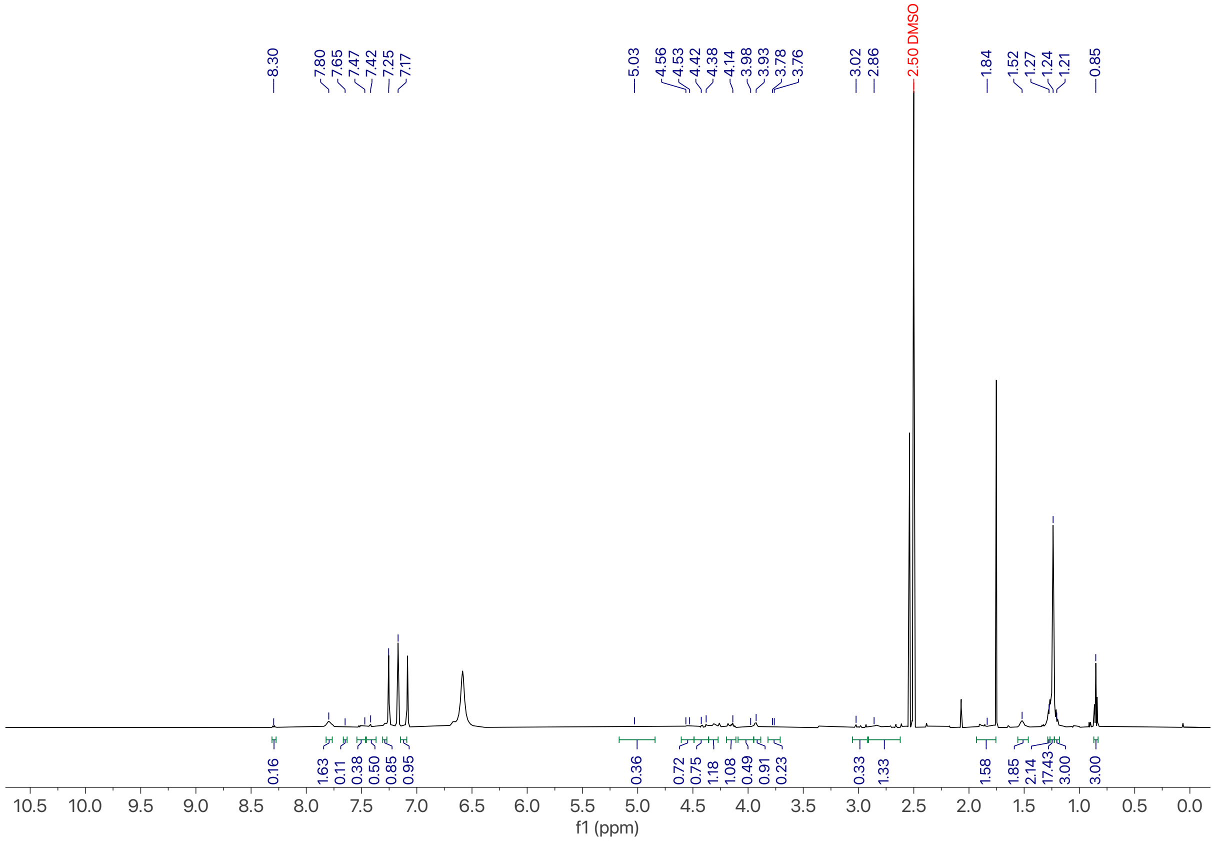

Assignment Table

Residue	Amide NH	Hα	Hβ	Hγ	other
Lipid		3.93	1.52	1.27	C4-15: 1.24, C16: 0.85
Dab1	7.47 (d, *J* = 8.4 Hz)	4.56	1.84	2.86	NH: 7.80, 7.17
Prs2		5.03	3.98, 3.76		δ: 4.14, NMe: 3.02
Ala3	7.65 (d, *J* = 6.7 Hz)	4.42	1.21		
Prs4	8.30 (d, *J* = 8.1 Hz)	4.53	3.78		δ: 4.38, NH: 7.42, 7.25

Compound 11: Alkynomycin A16

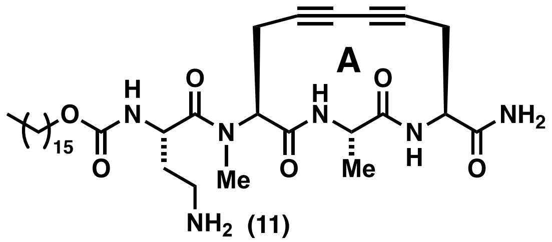

Chemical Formula: $C_{35}H_{58}N_6O_6$

Molecular Weight: 658.89

The product was purified by mass-directed prep LC. The crude material was purified on a Waters Autopurification LC with a Waters BEH C18 column (5 um, 19x160 mm) using a 0.1% aqueous trifluoroacetic acid:acetonitrile gradient (30 mL/min, main segment of gradient at 55-75% acetonitrile over 8 minutes) at ambient temperature. Fractionation was triggered by a Waters QDa single quadrupole mass spec (ESI+).
Retention time: 5.6 min

HRMS:

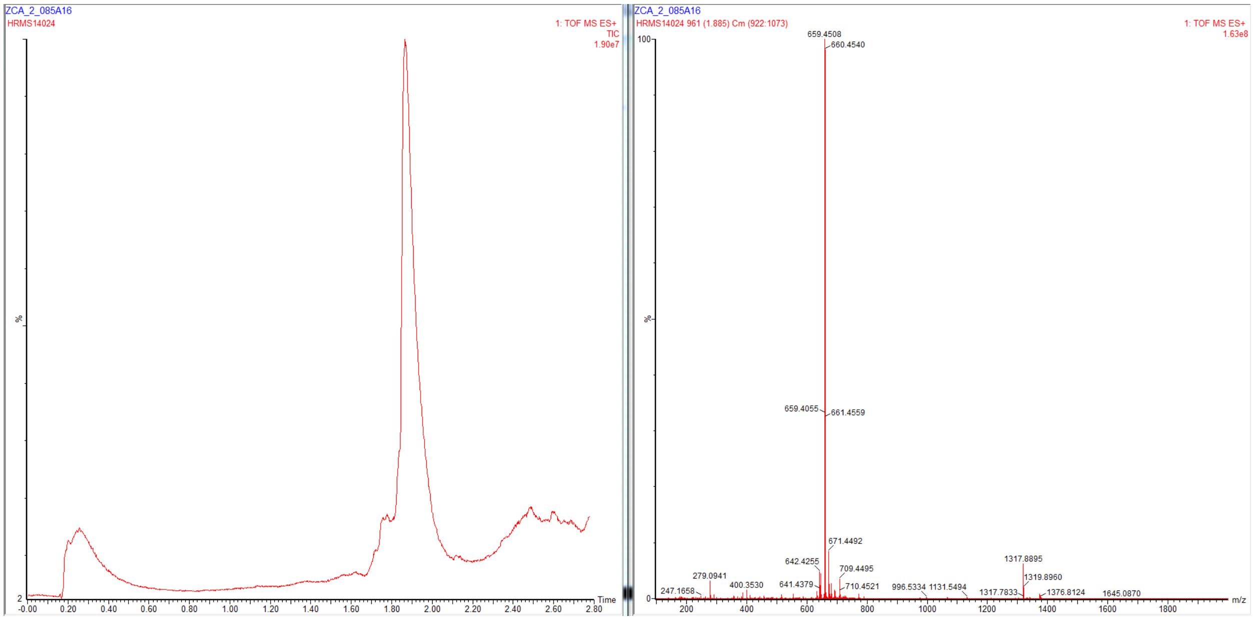

[1]H Spectrum

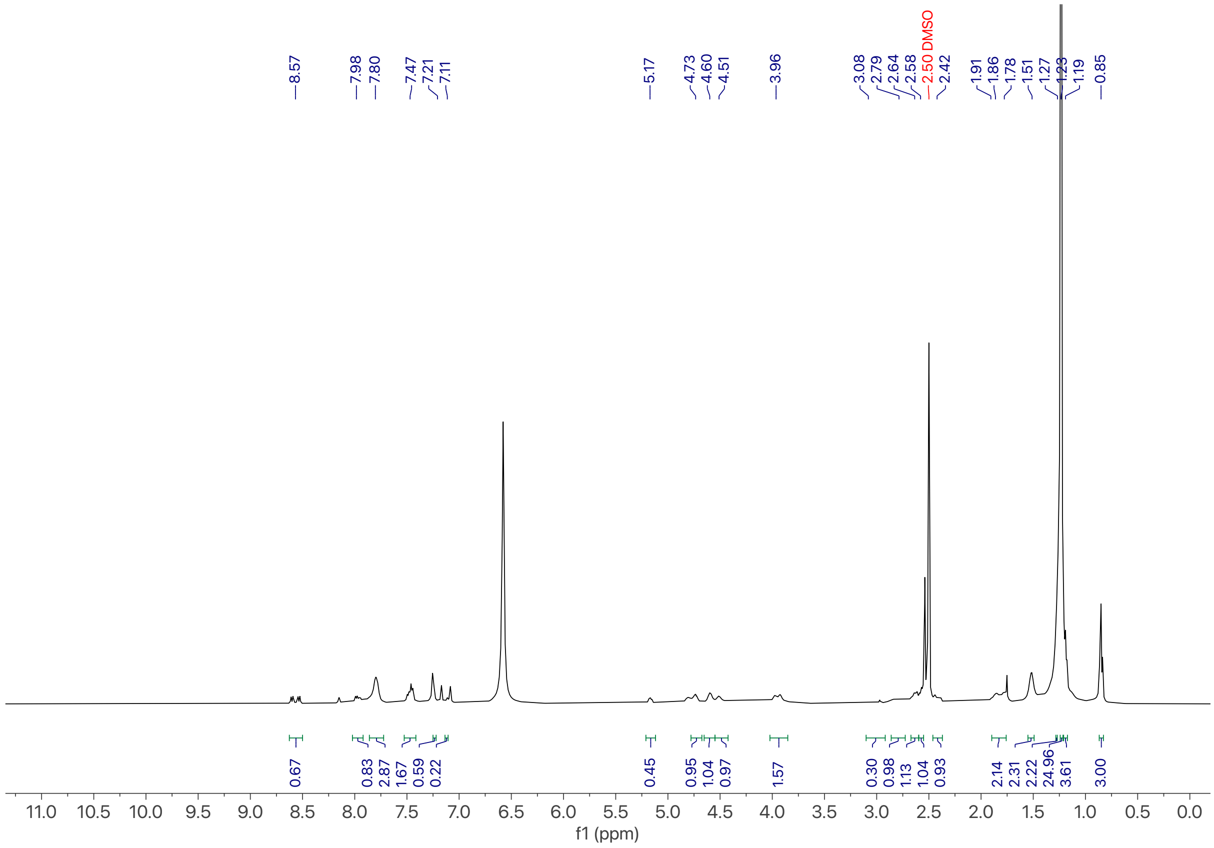

Assignment Table

Residue	Amide NH	Hα	Hβ	Hγ	other
**Lipid**		3.96	1.51	1.27	C4-15: 1.23, C16: 0.85
**Dab1**	7.47 (d, *J* = 8.5 Hz)	4.51	1.85, 1.78	2.85, 2.72	NH: 7.80, 7.11
**Pra2**		5.17	2.79, 2.42		NMe: 3.08
**Ala3**	7.98 (d, *J* = 8.7 Hz)	4.60	1.19		
**Pra4**	8.57 (d, *J* = 10.3 Hz)	4.73	2.64, 2.58		NH: 7.21

Compound S2: Alkynomycin B16

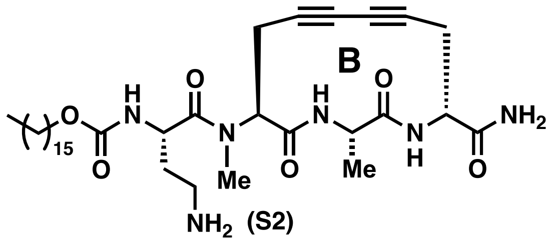

Chemical Formula: $C_{35}H_{58}N_6O_6$

Molecular Weight: 658.89

The product was purified by mass-directed prep LC. The crude material was purified on a Waters Autopurification LC with a Waters BEH C18 column (5 um, 19x160 mm) using under isocratic conditions (50% B - acetonitrile / 50% A - 0.1% aqueous trifluoroacetic acid; 30 mL/min for 50 minutes) at ambient temperature. Fractionation was triggered by a Waters QDa single quadrupole mass spec (ESI+).
Retention time: 10.2 min

HRMS:

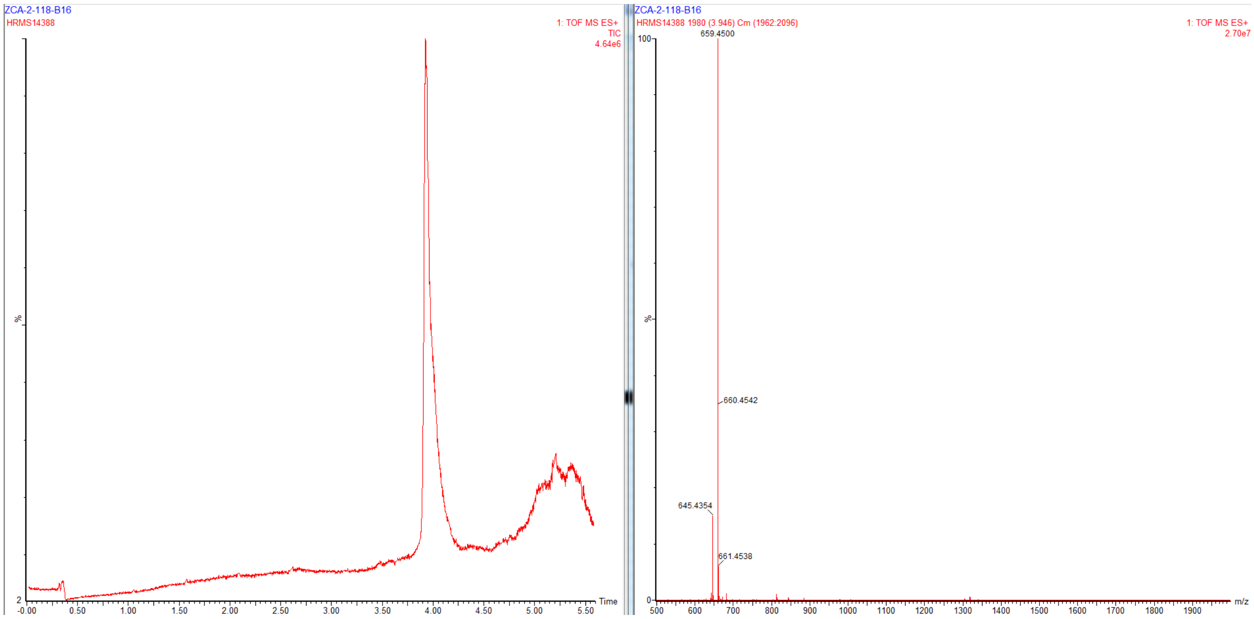

^{1}H Spectrum

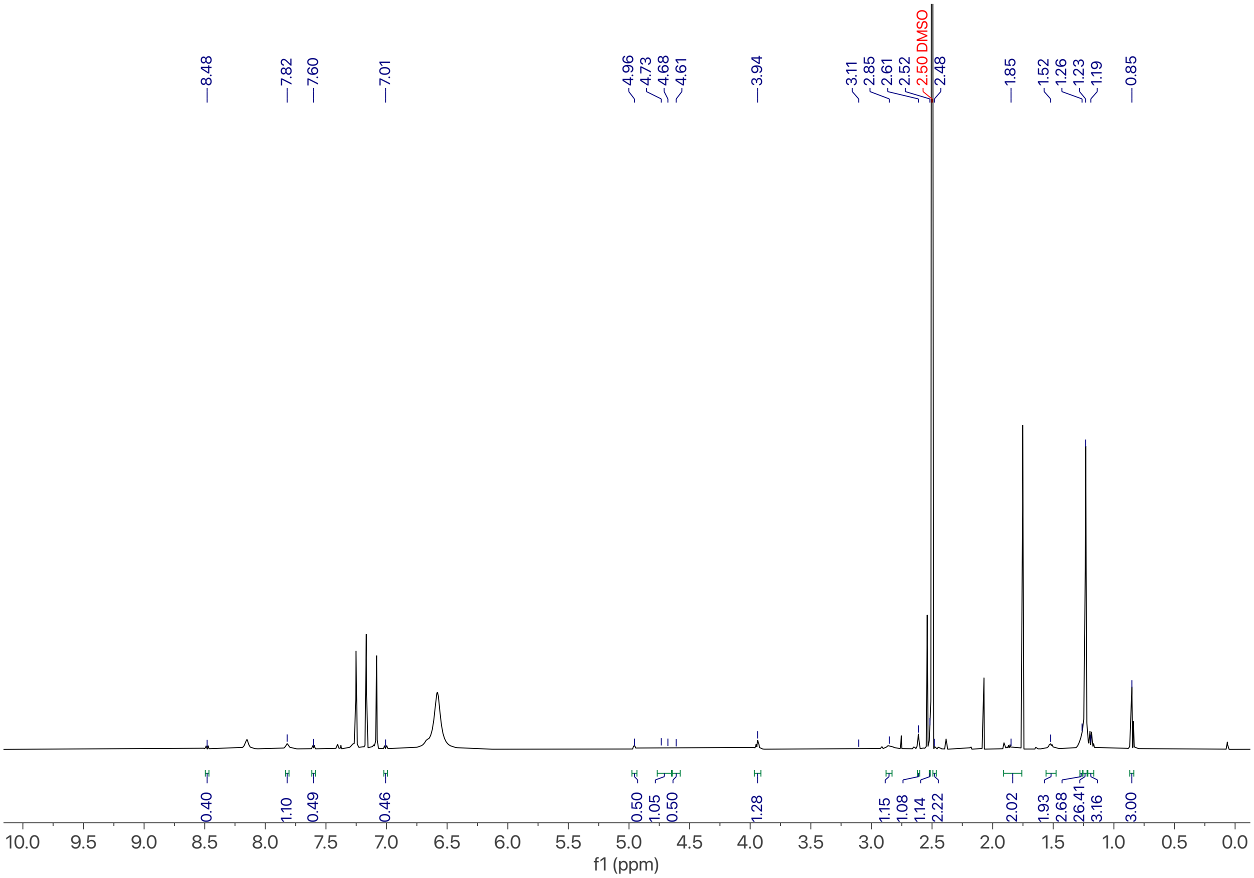

Assignment Table

Residue	Amide NH	Hα	Hβ	Hγ	other
**Lipid**		3.95	1.52	1.26	C4-15: 1.23, C16: 0.85
**Dab1**	7.60 (d, J = 6.9 Hz)	4.61	1.85	2.85	NH: 7.85
**Pra2**		4.96	2.47		NMe: 3.10
**Ala3**	7.01 (d, J = 9.9 Hz)	4.68	1.19		
**D-Pra4**	8.48 (d, J = 10.5 Hz)	4.73	2.61, 2.52		

Compound 13: Alkynomycin C16

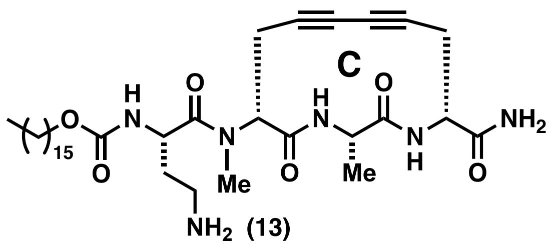

(13)

Chemical Formula: $C_{35}H_{58}N_6O_6$

Molecular Weight: 658.89

The product was purified by mass-directed prep LC. The crude material was purified on a Waters Autopurification LC with a Waters BEH C18 column (5 um, 19x160 mm) using under isocratic conditions (55% B - acetonitrile / 45% A - 0.1% aqueous trifluoroacetic acid; 30 mL/min for 13.5 minutes) at ambient temperature. Fractionation was triggered by a Waters QDa single quadrupole mass spec (ESI+).
Retention time: 7.2 min

HRMS:

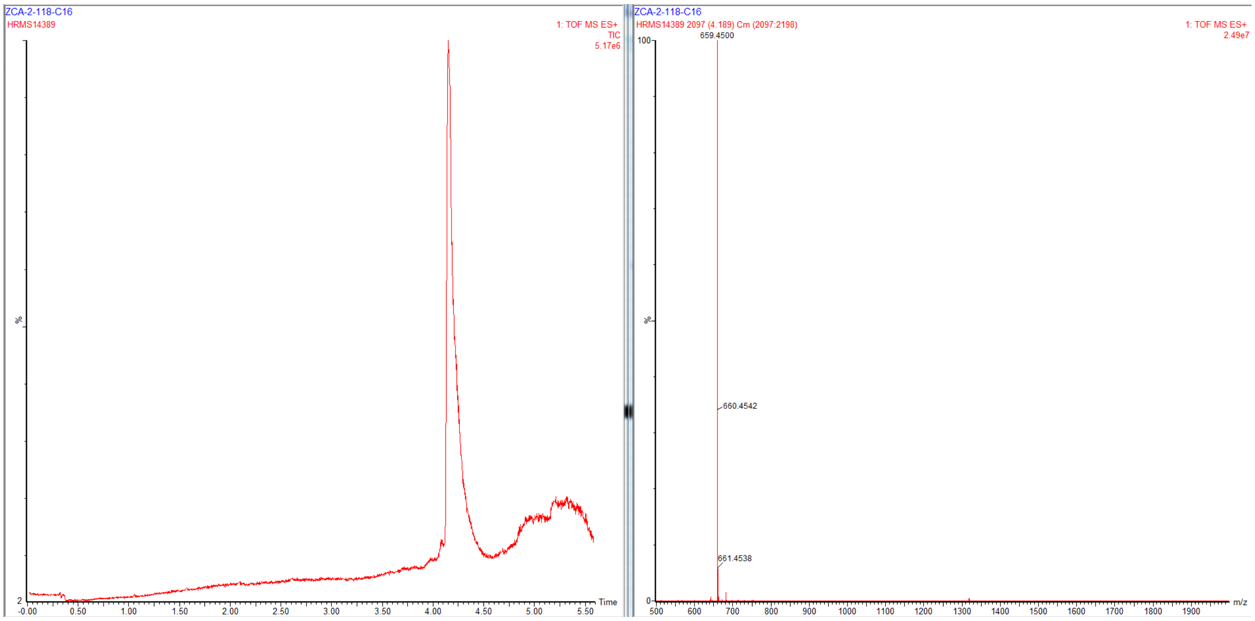

[1]H Spectrum

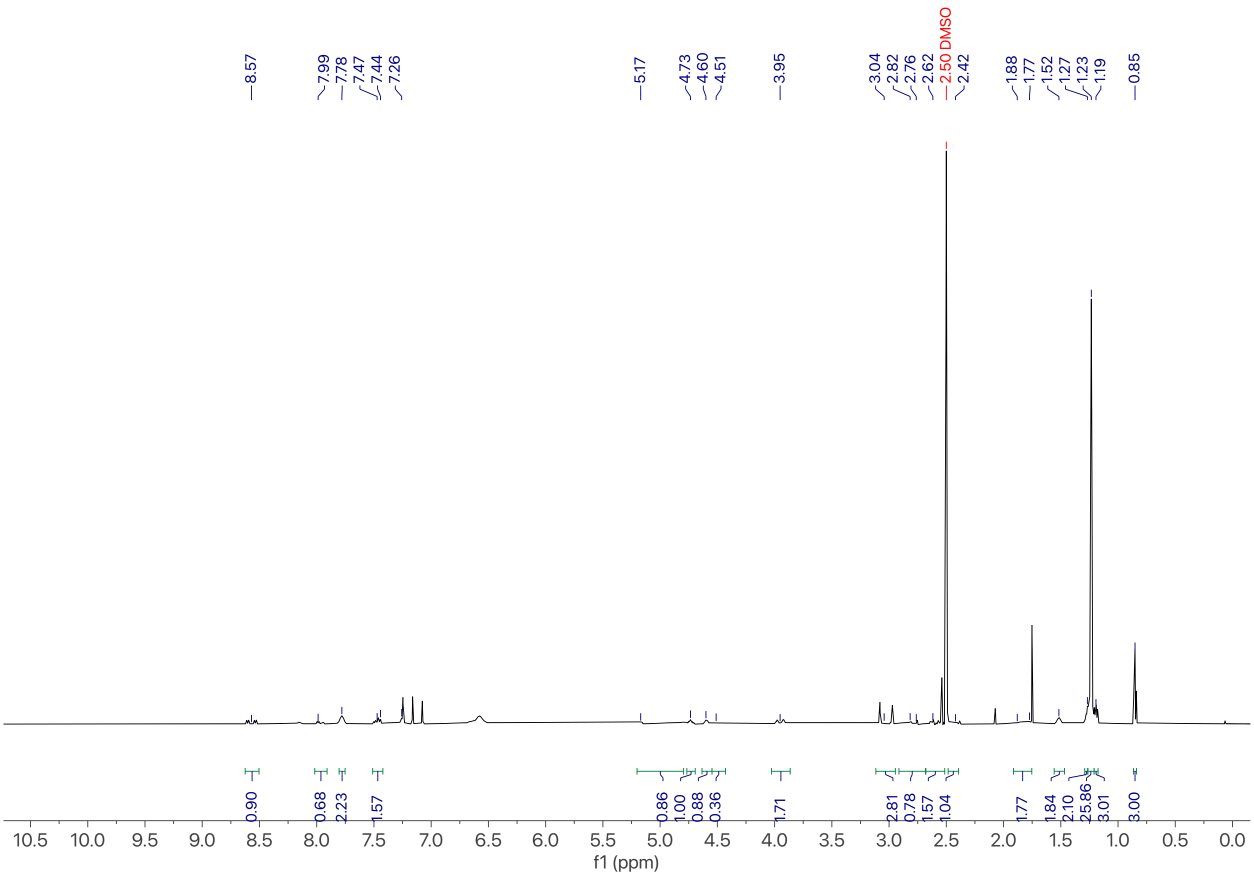

Assignment Table

Residue	Amide NH	Hα	Hβ	Hγ	other
**Lipid**		3.95	1.52	1.27	C4-15: 1.23, C16: 0.85
**Dab1**	7.47	4.51	1.88, 1.77	2.83	NH: 7.78, 7.12
**D-Pra2**		5.17	2.76, 2.42		NMe: 3.04
**Ala3**	7.99 (d, *J* = 8.4 Hz)	4.60	1.19		
**D-Pra4**	8.57 (d, *J* = 9.9 Hz)	4.73	2.62		NH: 7.44, 7.26

Compound S4: Alkynomycin D16

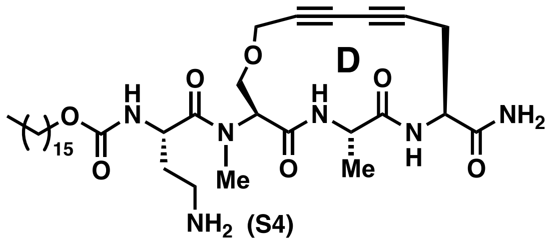

Chemical Formula: $C_{36}H_{60}N_6O_7$

Molecular Weight: 688.91

The product was purified by mass-directed prep LC. The crude material was purified on a Waters Autopurification LC with a Waters BEH C18 column (5 um, 19x160 mm) using under isocratic conditions (55% B - acetonitrile / 45% A - 0.1% aqueous trifluoroacetic acid; 30 mL/min for 15 minutes) at ambient temperature. Fractionation was triggered by a Waters QDa single quadrupole mass spec (ESI+).
Retention time: 6.5 min

HRMS:

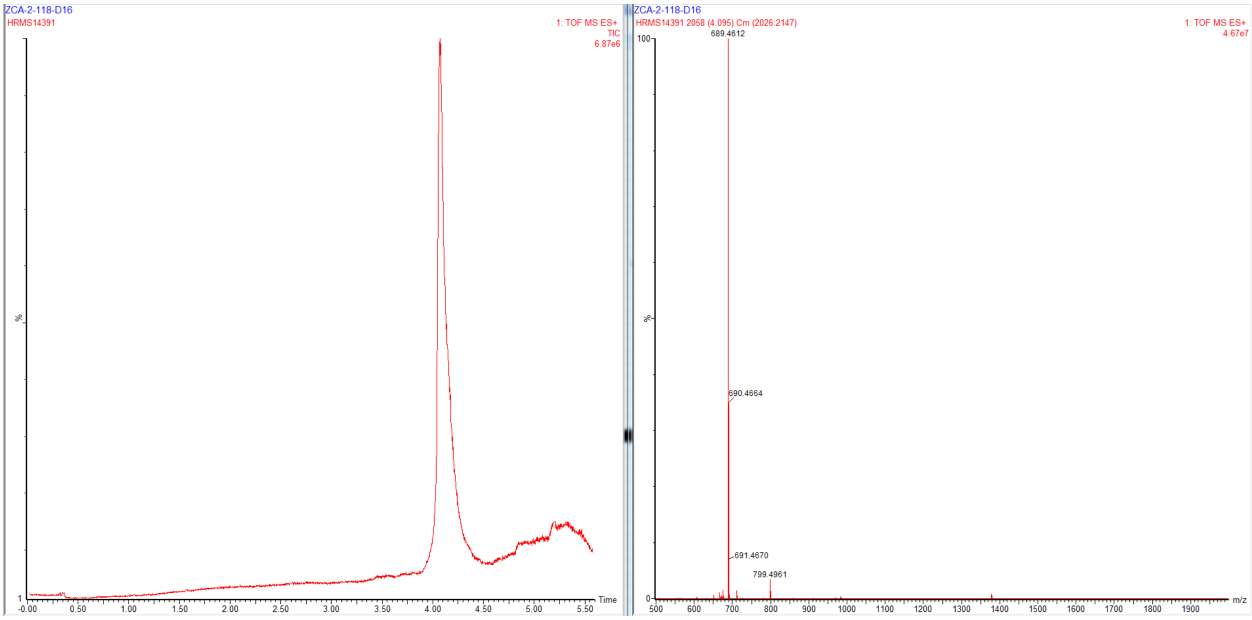

^{1}H Spectrum

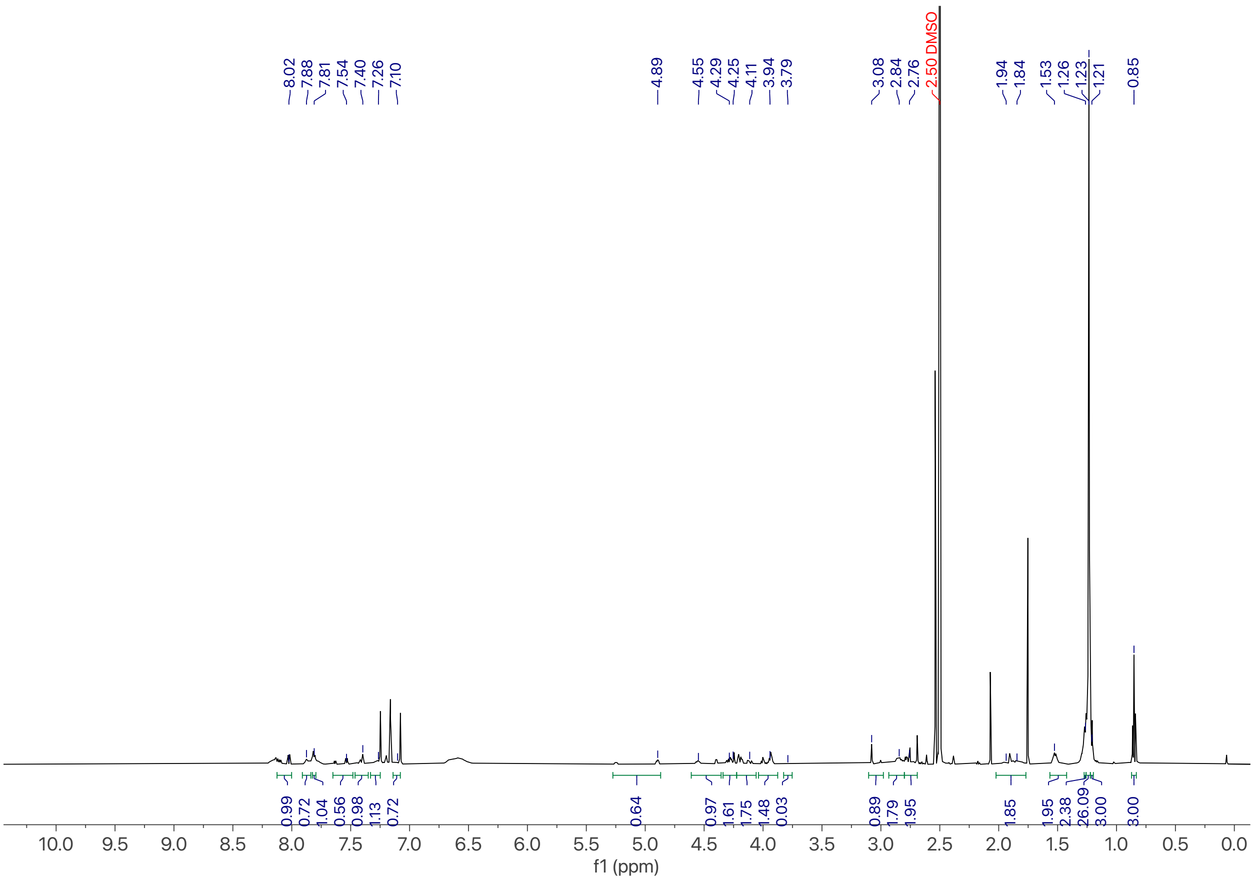

Assignment Table

Residue	Amide NH	Hα	Hβ	Hγ	other
**Lipid**		3.94	1.52	1.27	C4-15: 1.23, C16: 0.85
**Dab1**	7.53 (d, *J* = 8.2 Hz)	4.55	1.94, 1.84	2.84	NH: 7.85, 7.10
**Prs2**		4.89	3.79		δ: 4.11, NMe: 3.08
**Ala3**	7.81	4.29	1.21		
**Pra4**	8.03 (d, *J* = 8.1 Hz)	4.25	2.76		NH: 7.40, 7.26

Compound S6: Alkynomycin E16

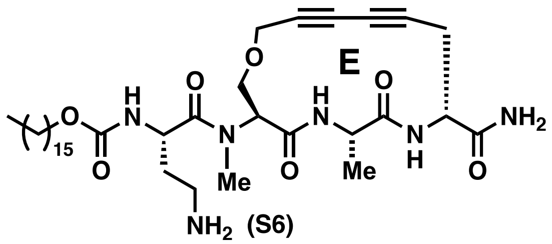

Chemical Formula: $C_{36}H_{60}N_6O_7$

Molecular Weight: 688.91

The product was purified by mass-directed prep LC. The crude material was purified on a Waters Autopurification LC with a Waters BEH C18 column (5 um, 19x160 mm) using under isocratic conditions (55% B - acetonitrile / 45% A - 0.1% aqueous trifluoroacetic acid; 30 mL/min for 15 minutes) at ambient temperature. Fractionation was triggered by a Waters QDa single quadrupole mass spec (ESI+).
Retention time: 7.7 min

HRMS:

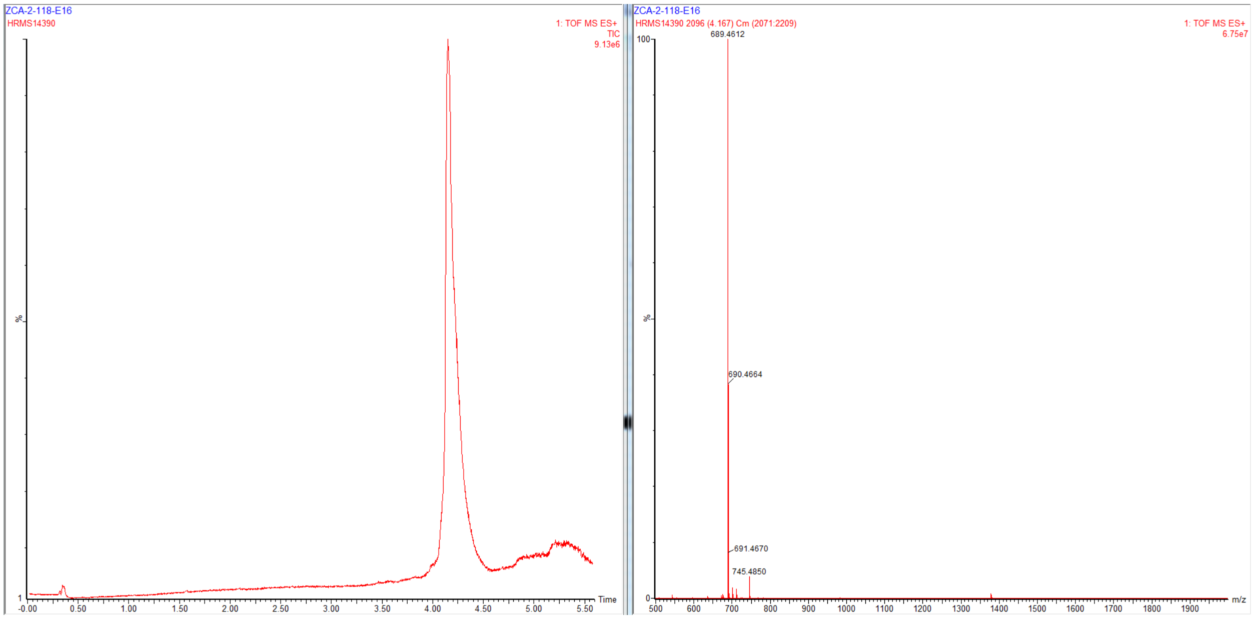

[1]H Spectrum

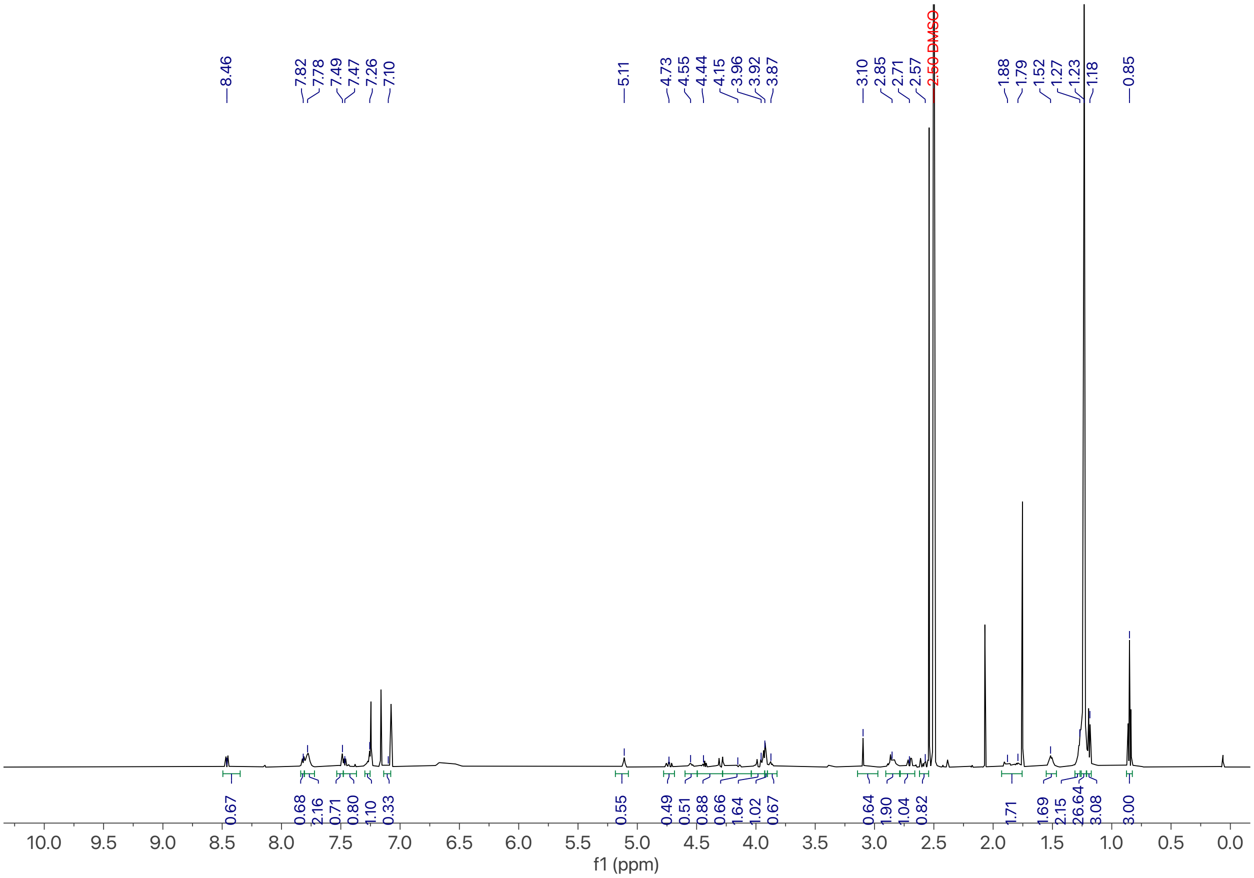

Assignment Table

Residue	Amide NH	Hα	Hβ	Hγ	other
**Lipid**		3.96	1.53	1.28	C4-15: 1.24, C16: 0.85
**Dab1**	7.47 (d, *J* = 8.3 Hz)	4.55	1.88, 1.79	2.85	NH: 7.78, 7.10
**Prs2**		5.11	3.87, 3.92		δ: 4.15, NMe: 3.10
**Ala3**	7.82 (d, *J* = 7.9 Hz)	4.44	1.20		
**D-Pra4**	8.46 (d, *J* = 10.2 Hz)	4.73	2.71, 2.57		NH: 7.49, 7.26

Compound 15: Alkynomycin F16

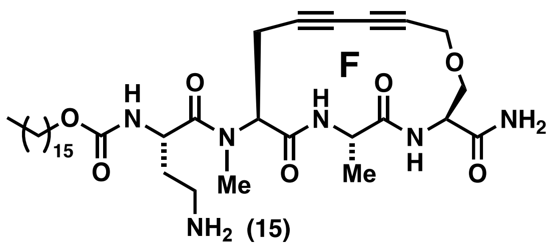

Chemical Formula: $C_{36}H_{60}N_6O_7$

Molecular Weight: 688.91

The product was purified by mass-directed prep LC. The crude material was purified on a Waters Autopurification LC with a Waters BEH C18 column (5 um, 19x160 mm) using under isocratic conditions (50% B - acetonitrile / 50% A - 0.1% aqueous trifluoroacetic acid; 30 mL/min for 30 minutes) at ambient temperature. Fractionation was triggered by a Waters QDa single quadrupole mass spec (ESI+).
Retention time: 17.4 min

HRMS:

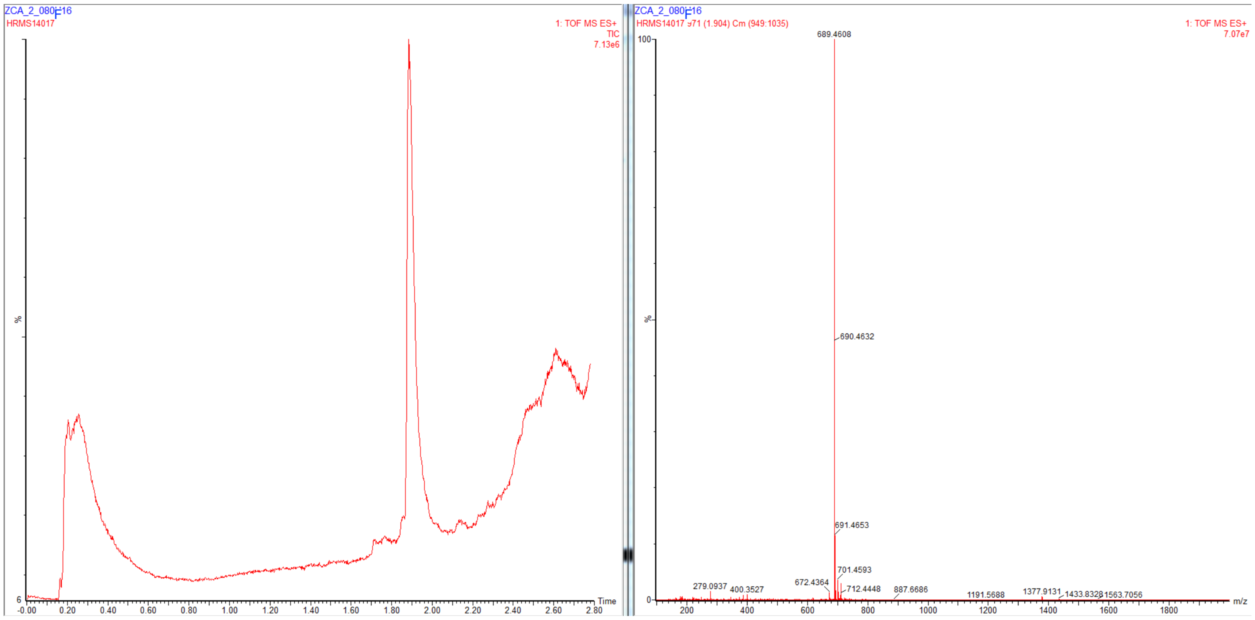

[1]H Spectrum

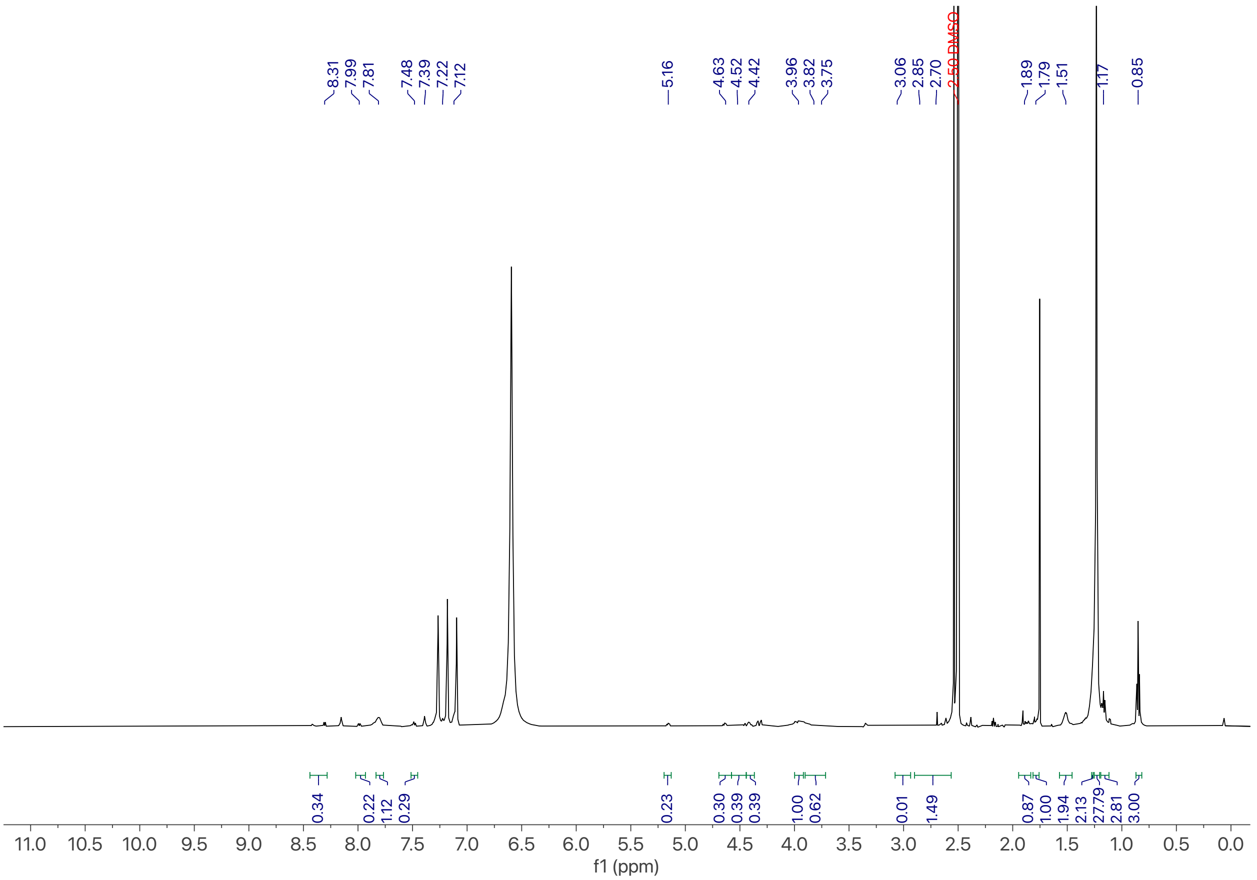

Assignment Table

Residue	Amide NH	Hα	Hβ	Hγ	other
**Lipid**		3.96	1.52	1.24	C4-11: 1.24, C12: 0.85 (t)
**Dab1**	7.48 (d, *J* = 8.5 Hz)	4.52	1.89, 1.79	2.85	NH: 7.81, 7.12
**Pra2**		5.16	2.70		NMe: 3.06
**Ala3**	7.99 (d, *J* = 7.9 Hz)	4.63	1.17		
**Prs4**	8.31 (d, *J* = 7.6 Hz)	4.42	3.82, 3.75		NH: 7.39, 7.22

Compound S8: Alkynomycin G16

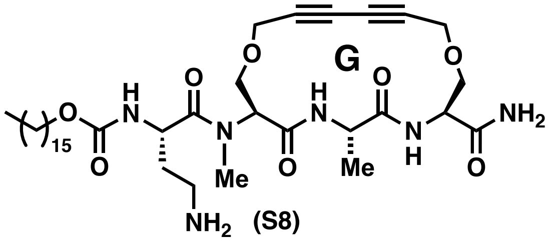

(S8)

Chemical Formula: $C_{37}H_{62}N_6O_8$

Molecular Weight: 718.94

The product was purified by mass-directed prep LC. The crude material was purified on a Waters Autopurification LC with a Waters BEH C18 column (5 um, 19x160 mm) using under isocratic conditions (55% B - acetonitrile / 45% A - 0.1% aqueous trifluoroacetic acid; 30 mL/min for 15 minutes) at ambient temperature. Fractionation was triggered by a Waters QDa single quadrupole mass spec (ESI+).
Retention time: 9.0 min

HRMS:

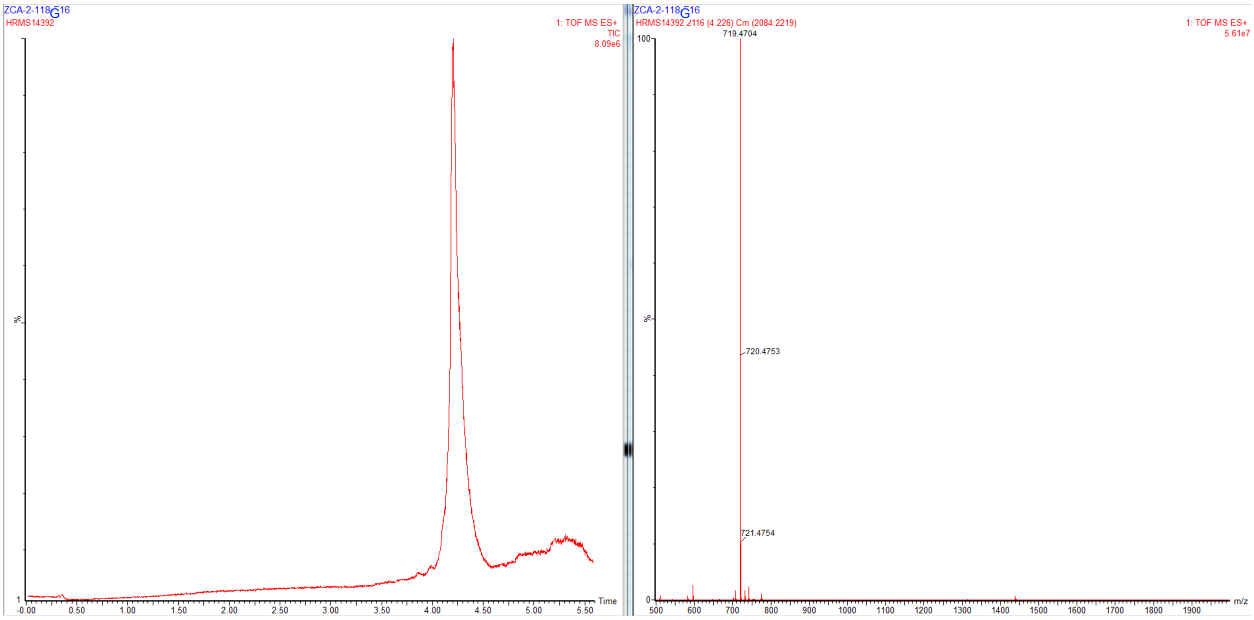

^{1}H Spectrum

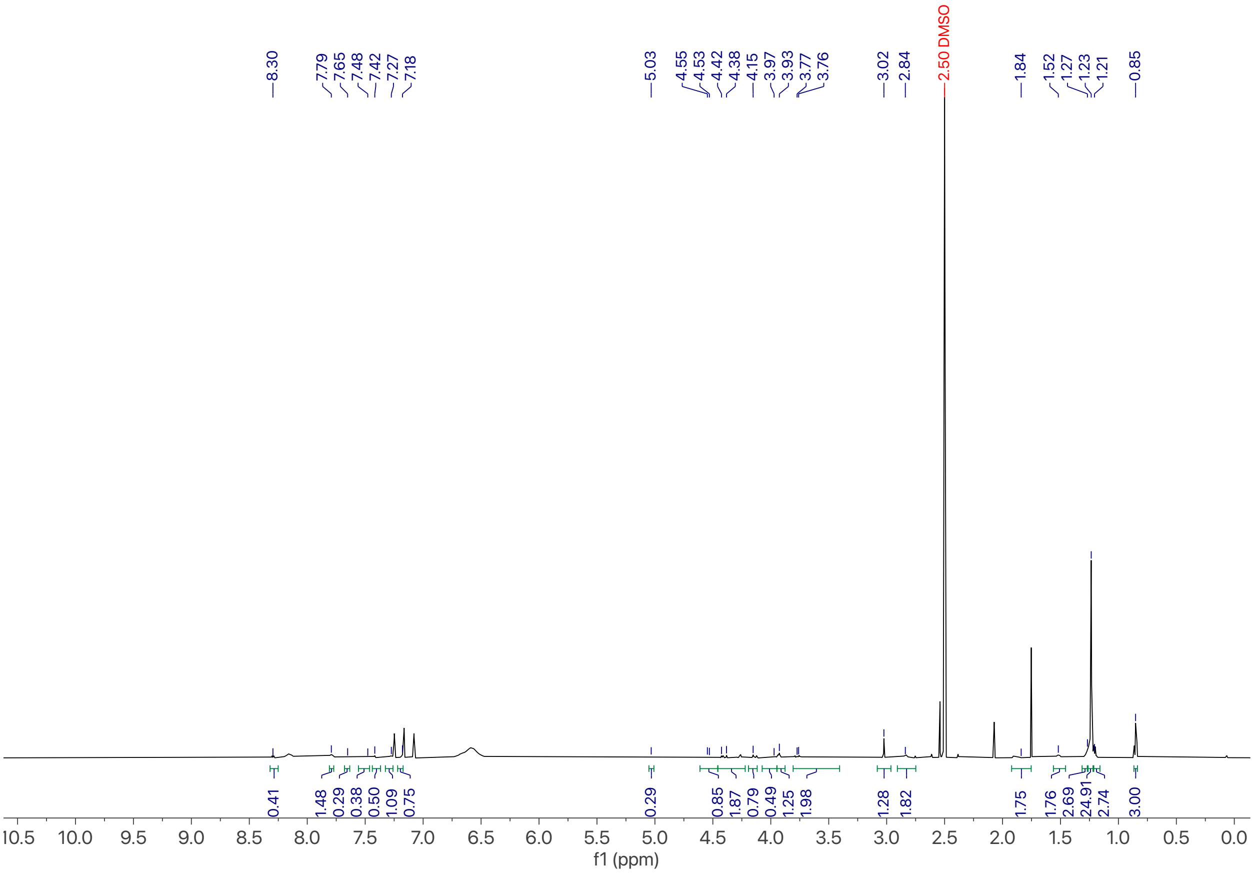

Assignment Table

Residue	Amide NH	Hα	Hβ	Hγ	other
**Lipid**		3.93	1.52	1.27	C4-15: 1.23, C16: 0.85
**Dab1**	7.48 (d, *J* = 8.0 Hz)	4.55	1.84	2.84	NH: 7.79, 7.18
**Prs2**		5.03	3.97, 3.76		δ: 4.15, NMe: 3.02
**Ala3**	7.65 (d, *J* = 6.7 Hz)	4.42	1.21		
**Prs4**	8.30 (d, *J* = 7.7 Hz)	4.53	3.77		δ: 4.38, NH: 7.42, 7.27

References

1. Schrödinger Release 2019-2: MacroModel. New York, NY: Schrödinger, LLC; 2019.

2. Frisch MJ, Trucks GW, Schlegel HB, Scuseria GE, Robb MA, Cheeseman JR*, et al.* Gaussian 16, Revision B.01. Wallingford CT; 2016.

3. Methods for dilution antimicrobial susceptiblity tests for bacteria that grow aerobically. In: Institute CaLS, editor. Wayne, PA; 2011.

4. Paetzel M, Goodall JJ, Kania M, Dalbey RE, Page MG. Crystallographic and biophysical analysis of a bacterial signal peptidase in complex with a lipopeptide-based inhibitor. *J Biol Chem* 2004, **279**(29)**:** 30781-30790.
